# Precision prognostics for cardiovascular disease in Type 2 diabetes: a systematic review and meta-analysis

**DOI:** 10.1038/s43856-023-00429-z

**Published:** 2024-01-22

**Authors:** Abrar Ahmad, Lee-Ling Lim, Mario Luca Morieri, Claudia Ha-ting Tam, Feifei Cheng, Tinashe Chikowore, Monika Dudenhöffer-Pfeifer, Hugo Fitipaldi, Chuiguo Huang, Sarah Kanbour, Sudipa Sarkar, Robert Wilhelm Koivula, Ayesha A. Motala, Sok Cin Tye, Gechang Yu, Yingchai Zhang, Michele Provenzano, Diana Sherifali, Russell J. de Souza, Deirdre Kay Tobias, ADA/EASD PMDI, Deirdre K. Tobias, Jordi Merino, Catherine Aiken, Jamie L. Benham, Dhanasekaran Bodhini, Amy L. Clark, Kevin Colclough, Rosa Corcoy, Sara J. Cromer, Daisy Duan, Jamie L. Felton, Ellen C. Francis, Pieter Gillard, Véronique Gingras, Romy Gaillard, Eram Haider, Alice Hughes, Jennifer M. Ikle, Laura M. Jacobsen, Anna R. Kahkoska, Jarno L. T. Kettunen, Raymond J. Kreienkamp, Lee-Ling Lim, Jonna M. E. Männistö, Robert Massey, Niamh-Maire Mclennan, Rachel G. Miller, Jasper Most, Rochelle N. Naylor, Bige Ozkan, Kashyap Amratlal Patel, Scott J. Pilla, Katsiaryna Prystupa, Sridharan Raghavan, Mary R. Rooney, Martin Schön, Zhila Semnani-Azad, Magdalena Sevilla-Gonzalez, Pernille Svalastoga, Wubet Worku Takele, Claudia Ha-ting Tam, Anne Cathrine B. Thuesen, Mustafa Tosur, Amelia S. Wallace, Caroline C. Wang, Jessie J. Wong, Jennifer M. Yamamoto, Katherine Young, Chloé Amouyal, Mette K. Andersen, Maxine P. Bonham, Mingling Chen, Tinashe Chikowore, Sian C. Chivers, Christoffer Clemmensen, Dana Dabelea, Adem Y. Dawed, Aaron J. Deutsch, Laura T. Dickens, Linda A. DiMeglio, Carmella Evans-Molina, María Mercè Fernández-Balsells, Stephanie L. Fitzpatrick, Stephen E. Gitelman, Mark O. Goodarzi, Jessica A. Grieger, Marta Guasch-Ferré, Nahal Habibi, Torben Hansen, Chuiguo Huang, Arianna Harris-Kawano, Heba M. Ismail, Benjamin Hoag, Randi K. Johnson, Angus G. Jones, Robert W. Koivula, Aaron Leong, Gloria K. W. Leung, Ingrid M. Libman, Kai Liu, S. Alice Long, William L. Lowe, Robert W. Morton, Suna Onengut-Gumuscu, James S. Pankow, Maleesa Pathirana, Sofia Pazmino, Dianna Perez, John R. Petrie, Camille E. Powe, Alejandra Quinteros, Rashmi Jain, Debashree Ray, Mathias Ried-Larsen, Zeb Saeed, Vanessa Santhakumar, Sarah Kanbour, Sudipa Sarkar, Gabriela S. F. Monaco, Denise M. Scholtens, Elizabeth Selvin, Wayne Huey-Herng Sheu, Cate Speake, Maggie A. Stanislawski, Nele Steenackers, Andrea K. Steck, Norbert Stefan, Julie Støy, Rachael Taylor, Gebresilasea Gendisha Ukke, Marzhan Urazbayeva, Bart Van der Schueren, Camille Vatier, John M. Wentworth, Wesley Hannah, Sara L. White, Gechang Yu, Yingchai Zhang, Shao J. Zhou, Jacques Beltrand, Michel Polak, Ingvild Aukrust, Elisa de Franco, Sarah E. Flanagan, Kristin A. Maloney, Andrew McGovern, Janne Molnes, Mariam Nakabuye, Pål Rasmus Njølstad, Hugo Pomares-Millan, Cécile Saint-Martin, Cuilin Zhang, Yeyi Zhu, Sungyoung Auh, Russell de Souza, Andrea J. Fawcett, Chandra Gruber, Eskedar Getie Mekonnen, Emily Mixter, Diana Sherifali, Robert H. Eckel, John J. Nolan, Louis H. Philipson, Rebecca J. Brown, Liana K. Billings, Kristen Boyle, Tina Costacou, John M. Dennis, Jose C. Florez, Anna L. Gloyn, Peter A. Gottlieb, Siri Atma W. Greeley, Kurt Griffin, Andrew T. Hattersley, Irl B. Hirsch, Marie-France Hivert, Korey K. Hood, Jami L. Josefson, Soo Heon Kwak, Lori M. Laffel, Siew S. Lim, Ruth J. F. Loos, Ronald C. W. Ma, Chantal Mathieu, Nestoras Mathioudakis, James B. Meigs, Shivani Misra, Viswanathan Mohan, Rinki Murphy, Richard Oram, Katharine R. Owen, Susan E. Ozanne, Ewan R. Pearson, Wei Perng, Toni I. Pollin, Rodica Pop-Busui, Richard E. Pratley, Leanne M. Redman, Maria J. Redondo, Rebecca M. Reynolds, Robert K. Semple, Jennifer L. Sherr, Emily K. Sims, Arianne Sweeting, Tiinamaija Tuomi, Miriam S. Udler, Kimberly K. Vesco, Tina Vilsbøll, Robert Wagner, Stephen S. Rich, Paul W. Franks, Maria F. Gomez, Ronald C. W. Ma, Nestoras Mathioudakis

**Affiliations:** 1 Department of Clinical Sciences, Lund University Diabetes Centre, Lund University, Malmö, Sweden; 2 Department of Medicine, Faculty of Medicine, University of Malaya, Kuala Lumpur, Malaysia; 3 Department of Medicine and Therapeutics, The Chinese University of Hong Kong, Hong Kong SAR, China; 4 Asia Diabetes Foundation, Hong Kong SAR, China; 5 Metabolic Disease Unit, University Hospital of Padova, Padova, Italy; 6 Department of Medicine, University of Padova, Padova, Italy; 7 Laboratory for Molecular Epidemiology in Diabetes, Li Ka Shing Institute of Health Sciences, The Chinese University of Hong Kong, Hong Kong SAR, China; 8 Hong Kong Institute of Diabetes and Obesity, The Chinese University of Hong Kong, Hong Kong SAR, China; 9 Health Management Center, The Second Affiliated Hospital of Chongqing Medical University, Chongqing Medical University, Chongqing, China; 10 MRC/Wits Developmental Pathways for Health Research Unit, Department of Paediatrics, Faculty of Health Sciences, University of the Witwatersrand, Johannesburg, South Africa; 11 Sydney Brenner Institute for Molecular Bioscience, Faculty of Health Sciences, University of the Witwatersrand, Johannesburg, South Africa; 12 AMAN Hospital, Doha, Qatar; 13 Department of Medicine, Johns Hopkins University School of Medicine, Baltimore, Maryland USA; 14 Oxford Centre for Diabetes, Endocrinology and Metabolism, University of Oxford, Oxford, United Kingdom; 15 Department of Diabetes and Endocrinology, Nelson R Mandela School of Medicine, University of KwaZulu-Natal, Durban, South Africa; 16 Department of Clinical Pharmacy and Pharmacology, University Medical Center Groningen, Groningen, the Netherlands; 17 Sections on Genetics and Epidemiology, Joslin Diabetes Center, Harvard Medical School, Boston, Massachusetts USA; 18 Nephrology, Dialysis and Renal Transplant Unit, IRCCS—Azienda Ospedaliero-Universitaria di Bologna, Alma Mater Studiorum University of Bologna, Bologna, Italy; 19 Heather M. Arthur Population Health Research Institute, McMaster University, Ontario, Canada; 20 Department of Health Research Methods, Evidence, and Impact, Faculty of Health Sciences, McMaster University, Hamilton, Ontario Canada; 21 Population Health Research Institute, Hamilton Health Sciences Corporation, Hamilton, Ontario Canada; 22 Harvard T.H. Chan School of Public Health, Boston, Massachusetts USA; 23 Faculty of Health, Aarhus University, Aarhus, Denmark; 24 Division of Preventative Medicine, Department of Medicine, Brigham and Women’s Hospital and Harvard Medical School, Boston, MA USA; 25 Department of Nutrition, Harvard T.H. Chan School of Public Health, Boston, MA USA; 26 Novo Nordisk Foundation Center for Basic Metabolic Research, Faculty of Health and Medical Sciences, University of Copenhagen, Copenhagen, Denmark; 27 Diabetes Unit, Endocrine Division, Massachusetts General Hospital, Boston, MA USA; 28 Center for Genomic Medicine, Massachusetts General Hospital, Boston, MA USA; 29 Department of Obstetrics and Gynaecology, the Rosie Hospital, Cambridge, UK; 30 NIHR Cambridge Biomedical Research Centre, University of Cambridge, Cambridge, UK; 31 Departments of Medicine and Community Health Sciences, Cumming School of Medicine, University of Calgary, Calgary, AB Canada; 32 Department of Molecular Genetics, Madras Diabetes Research Foundation, Chennai, India; 33 Division of Pediatric Endocrinology, Department of Pediatrics, Saint Louis University School of Medicine, SSM Health Cardinal Glennon Children’s Hospital, St. Louis, MO USA; 34 Department of Clinical and Biomedical Sciences, University of Exeter Medical School, Exeter, Devon, UK; 35 CIBER-BBN, ISCIII, Madrid, Spain; 36 Institut d’Investigació Biomèdica Sant Pau (IIB SANT PAU), Barcelona, Spain; 37 Departament de Medicina, Universitat Autònoma de Barcelona, Bellaterra, Spain; 38 Programs in Metabolism and Medical & Population Genetics, Broad Institute, Cambridge, MA USA; 39 Department of Medicine, Harvard Medical School, Boston, MA USA; 40 Division of Endocrinology, Diabetes and Metabolism, Johns Hopkins University School of Medicine, Baltimore, MD USA; 41 Department of Pediatrics, Indiana University School of Medicine, Indianapolis, IN USA; 42 Herman B Wells Center for Pediatric Research, Indiana University School of Medicine, Indianapolis, IN USA; 43 Center for Diabetes and Metabolic Diseases, Indiana University School of Medicine, Indianapolis, IN USA; 44 Department of Biostatistics and Epidemiology, Rutgers School of Public Health, Piscataway, NJ USA; 45 University Hospital Leuven, Leuven, Belgium; 46 Department of Nutrition, Université de Montréal, Montreal, Quebec Canada; 47 Research Center, Sainte-Justine University Hospital Center, Montreal, Quebec Canada; 48 Department of Pediatrics, Erasmus Medical Center, Rotterdam, The Netherlands; 49 Division of Population Health & Genomics, School of Medicine, University of Dundee, Dundee, UK; 50 Department of Pediatrics, Stanford School of Medicine, Stanford University, Stanford, CA USA; 51 Stanford Diabetes Research Center, Stanford School of Medicine, Stanford University, Stanford, CA USA; 52 University of Florida, Gainesville, FL USA; 53 Department of Nutrition, University of North Carolina at Chapel Hill, Chapel Hill, NC USA; 54 Helsinki University Hospital, Abdominal Centre/Endocrinology, Helsinki, Finland; 55 Folkhalsan Research Center, Helsinki, Finland; 56 Institute for Molecular Medicine Finland FIMM, University of Helsinki, Helsinki, Finland; 57 Department of Pediatrics, Division of Endocrinology, Boston Children’s Hospital, Boston, MA USA; 58 Department of Medicine & Therapeutics, Chinese University of Hong Kong, Hong Kong SAR, China; 59 Departments of Pediatrics and Clinical Genetics, Kuopio University Hospital, Kuopio, Finland; 60 Department of Medicine, University of Eastern Finland, Kuopio, Finland; 61 Centre for Cardiovascular Science, Queen’s Medical Research Institute, University of Edinburgh, Edinburgh, UK; 62 Department of Epidemiology, University of Pittsburgh, Pittsburgh, PA USA; 63 Department of Orthopedics, Zuyderland Medical Center, Sittard-Geleen, The Netherlands; 64 Departments of Pediatrics and Medicine, University of Chicago, Chicago, Illinois USA; 65 Welch Center for Prevention, Epidemiology, and Clinical Research, Johns Hopkins Bloomberg School of Public Health, Baltimore, Maryland USA; 66 Ciccarone Center for the Prevention of Cardiovascular Disease, Johns Hopkins School of Medicine, Baltimore, MD USA; 67 Department of Medicine, Johns Hopkins University, Baltimore, MD USA; 68 Department of Health Policy and Management, Johns Hopkins University Bloomberg School of Public Health, Baltimore, Maryland USA; 69 Institute for Clinical Diabetology, German Diabetes Center, Leibniz Center for Diabetes Research at Heinrich Heine University Düsseldorf, Auf’m Hennekamp 65, 40225 Düsseldorf, Germany; 70 German Center for Diabetes Research (DZD), Ingolstädter Landstraße 1, 85764 Neuherberg, Germany; 71 Section of Academic Primary Care, US Department of Veterans Affairs Eastern Colorado Health Care System, Aurora, CO USA; 72 Department of Medicine, University of Colorado School of Medicine, Aurora, CO USA; 73 Department of Epidemiology, Johns Hopkins Bloomberg School of Public Health, Baltimore, Maryland USA; 74 Institute of Experimental Endocrinology, Biomedical Research Center, Slovak Academy of Sciences, Bratislava, Slovakia; 75 Clinical and Translational Epidemiology Unit, Massachusetts General Hospital, Boston, MA USA; 76 Mohn Center for Diabetes Precision Medicine, Department of Clinical Science, University of Bergen, Bergen, Norway; 77 Children and Youth Clinic, Haukeland University Hospital, Bergen, Norway; 78 Eastern Health Clinical School, Monash University, Melbourne, Victoria Australia; 79 Department of Pediatrics, Baylor College of Medicine, Houston, TX USA; 80 Division of Pediatric Diabetes and Endocrinology, Texas Children’s Hospital, Houston, TX USA; 81 Children’s Nutrition Research Center, USDA/ARS, Houston, TX USA; 82 Stanford University School of Medicine, Stanford, CA USA; 83 Internal Medicine, University of Manitoba, Winnipeg, MB Canada; 84 Department of Diabetology, APHP, Paris, France; 85 Sorbonne Université, INSERM, NutriOmic team, Paris, France; 86 Department of Nutrition, Dietetics and Food, Monash University, Melbourne, Victoria Australia; 87 Monash Centre for Health Research and Implementation, Monash University, Clayton, VIC Australia; 88 Channing Division of Network Medicine, Brigham and Women’s Hospital, Boston, MA USA; 89 Department of Women and Children’s health, King’s College London, London, UK; 90 Lifecourse Epidemiology of Adiposity and Diabetes (LEAD) Center, University of Colorado Anschutz Medical Campus, Aurora, CO USA; 91 Section of Adult and Pediatric Endocrinology, Diabetes and Metabolism, Kovler Diabetes Center, University of Chicago, Chicago, USA; 92 Department of Pediatrics, Riley Hospital for Children, Indiana University School of Medicine, Indianapolis, IN USA; 93 Richard L. Roudebush VAMC, Indianapolis, IN USA; 94 Biomedical Research Institute Girona, IdIBGi, Girona, Spain; 95 Diabetes, Endocrinology and Nutrition Unit Girona, University Hospital Dr Josep Trueta, Girona, Spain; 96 Institute of Health System Science, Feinstein Institutes for Medical Research, Northwell Health, Manhasset, NY USA; 97 University of California at San Francisco, Department of Pediatrics, Diabetes Center, San Francisco, CA USA; 98 Division of Endocrinology, Diabetes and Metabolism, Cedars-Sinai Medical Center, Los Angeles, CA USA; 99 Department of Medicine, Cedars-Sinai Medical Center, Los Angeles, CA USA; 100 Adelaide Medical School, Faculty of Health and Medical Sciences, The University of Adelaide, Adelaide, Australia; 101 Robinson Research Institute, The University of Adelaide, Adelaide, Australia; 102 Department of Public Health and Novo Nordisk Foundation Center for Basic Metabolic Research, Faculty of Health and Medical Sciences, University of Copenhagen, 1014 Copenhagen, Denmark; 103 Division of Endocrinology and Diabetes, Department of Pediatrics, Sanford Children’s Hospital, Sioux Falls, SD USA; 104 University of South Dakota School of Medicine, E Clark St, Vermillion, SD USA; 105 Department of Biomedical Informatics, University of Colorado Anschutz Medical Campus, Aurora, CO USA; 106 Department of Epidemiology, Colorado School of Public Health, Aurora, CO USA; 107 Royal Devon University Healthcare NHS Foundation Trust, Exeter, UK; 108 Division of General Internal Medicine, Massachusetts General Hospital, Boston, MA USA; 109 UPMC Children’s Hospital of Pittsburgh, Pittsburgh, PA USA; 110 Center for Translational Immunology, Benaroya Research Institute, Seattle, WA USA; 111 Department of Medicine, Northwestern University Feinberg School of Medicine, Chicago, IL USA; 112 Department of Pathology & Molecular Medicine, McMaster University, Hamilton, Canada; 113 Population Health Research Institute, Hamilton, Canada; 114 Department of Translational Medicine, Medical Science, Novo Nordisk Foundation, Tuborg Havnevej 19, 2900 Hellerup, Denmark; 115 Center for Public Health Genomics, Department of Public Health Sciences, University of Virginia, Charlottesville, VA USA; 116 Division of Epidemiology and Community Health, School of Public Health, University of Minnesota, Minneapolis, MN USA; 117 Department of Chronic Diseases and Metabolism, Clinical and Experimental Endocrinology, KU Leuven, Leuven, Belgium; 118 School of Health and Wellbeing, College of Medical, Veterinary and Life Sciences, University of Glasgow, Glasgow, UK; 119 Department of Obstetrics, Gynecology, and Reproductive Biology, Massachusetts General Hospital and Harvard Medical School, Boston, MA USA; 120 Sanford Children’s Specialty Clinic, Sioux Falls, SD USA; 121 Department of Pediatrics, Sanford School of Medicine, University of South Dakota, Sioux Falls, SD USA; 122 Department of Biostatistics, Johns Hopkins Bloomberg School of Public Health, Baltimore, Maryland USA; 123 Centre for Physical Activity Research, Rigshospitalet, Copenhagen, Denmark; 124 Institute for Sports and Clinical Biomechanics, University of Southern Denmark, Odense, Denmark; 125 Department of Medicine, Division of Endocrinology, Diabetes and Metabolism, Indiana University School of Medicine, Indianapolis, IN USA; 126 Department of Preventive Medicine, Division of Biostatistics, Northwestern University Feinberg School of Medicine, Chicago, IL USA; 127 Institute of Molecular and Genomic Medicine, National Health Research Institutes, Taipei City, Taiwan; 128 Divsion of Endocrinology and Metabolism, Taichung Veterans General Hospital, Taichung, Taiwan; 129 Division of Endocrinology and Metabolism, Taipei Veterans General Hospital, Taipei, Taiwan; 130 Center for Interventional Immunology, Benaroya Research Institute, Seattle, WA USA; 131 Barbara Davis Center for Diabetes, University of Colorado Anschutz Medical Campus, Aurora, CO USA; 132 University Hospital of Tübingen, Tübingen, Germany; 133 Institute of Diabetes Research and Metabolic Diseases (IDM), Helmholtz Center Munich, Neuherberg, Germany; 134 Steno Diabetes Center Aarhus, Aarhus University Hospital, Aarhus, Denmark; 135 University of Newcastle, Newcastle upon Tyne, UK; 136 Gastroenterology, Baylor College of Medicine, Houston, TX USA; 137 Department of Endocrinology, University Hospitals Leuven, Leuven, Belgium; 138 Sorbonne University, Inserm U938, Saint-Antoine Research Centre, Institute of Cardiometabolism and Nutrition, Paris, 75012 France; 139 Department of Endocrinology, Diabetology and Reproductive Endocrinology, Assistance Publique-Hôpitaux de Paris, Saint-Antoine University Hospital, National Reference Center for Rare Diseases of Insulin Secretion and Insulin Sensitivity (PRISIS), Paris, France; 140 Royal Melbourne Hospital Department of Diabetes and Endocrinology, Parkville, Vic Australia; 141 Walter and Eliza Hall Institute, Parkville, Vic Australia; 142 University of Melbourne Department of Medicine, Parkville, Vic Australia; 143 Deakin University, Melbourne, Australia; 144 Department of Epidemiology, Madras Diabetes Research Foundation, Chennai, India; 145 Department of Diabetes and Endocrinology, Guy’s and St Thomas’ Hospitals NHS Foundation Trust, London, UK; 146 School of Agriculture, Food and Wine, University of Adelaide, Adelaide, Australia; 147 Institut Cochin, Inserm U 10116, Paris, France; 148 Pediatric endocrinology and diabetes, Hopital Necker Enfants Malades, APHP Centre, université de Paris, Paris, France; 149 Department of Medical Genetics, Haukeland University Hospital, Bergen, Norway; 150 Department of Medicine, University of Maryland School of Medicine, Baltimore, MD USA; 151 Department of Epidemiology, Geisel School of Medicine at Dartmouth, Hanover, NH USA; 152 Department of Medical Genetics, AP-HP Pitié-Salpêtrière Hospital, Sorbonne University, Paris, France; 153 Global Center for Asian Women’s Health, Yong Loo Lin School of Medicine, National University of Singapore, Singapore, Singapore; 154 Department of Obstetrics and Gynecology, Yong Loo Lin School of Medicine, National University of Singapore, Singapore, Singapore; 155 Kaiser Permanente Northern California Division of Research, Oakland, California USA; 156 Department of Epidemiology and Biostatistics, University of California San Francisco, California, USA; 157 National Institute of Diabetes and Digestive and Kidney Diseases, National Institutes of Health, Bethesda, MD USA; 158 Ann & Robert H. Lurie Children’s Hospital of Chicago, Department of Pediatrics, Northwestern University Feinberg School of Medicine, Chicago, IL USA; 159 Department of Clinical and Organizational Development, Chicago, IL USA; 160 American Diabetes Association, Arlington, Virginia USA; 161 College of Medicine and Health Sciences, University of Gondar, Gondar, Ethiopia; 162 Global Health Institute, Faculty of Medicine and Health Sciences, University of Antwerp, 2160 Antwerp, Belgium; 163 Department of Medicine and Kovler Diabetes Center, University of Chicago, Chicago, IL USA; 164 School of Nursing, Faculty of Health Sciences, McMaster University, Hamilton, Canada; 165 Division of Endocrinology, Metabolism, Diabetes, University of Colorado, Boulder, CO USA; 166 Department of Clinical Medicine, School of Medicine, Trinity College Dublin, Dublin, Ireland; 167 Department of Endocrinology, Wexford General Hospital, Wexford, Ireland; 168 Division of Endocrinology, NorthShore University HealthSystem, Skokie, IL USA; 169 Department of Medicine, Prtizker School of Medicine, University of Chicago, Chicago, IL USA; 170 Department of Genetics, Stanford School of Medicine, Stanford University, Stanford, CA USA; 171 Departments of Pediatrics and Medicine and Kovler Diabetes Center, University of Chicago, Chicago, USA; 172 Sanford Research, Sioux Falls, SD USA; 173 University of Washington School of Medicine, Seattle, WA USA; 174 Department of Population Medicine, Harvard Medical School, Harvard Pilgrim Health Care Institute, Boston, MA USA; 175 Department of Medicine, Universite de Sherbrooke, Sherbrooke, QC Canada; 176 Department of Internal Medicine, Seoul National University College of Medicine, Seoul National University Hospital, Seoul, Republic of Korea; 177 Joslin Diabetes Center, Harvard Medical School, Boston, MA USA; 178 Charles Bronfman Institute for Personalized Medicine, Icahn School of Medicine at Mount Sinai, New York, NY USA; 179 Broad Institute, Cambridge, MA USA; 180 Division of Metabolism, Digestion and Reproduction, Imperial College London, London, UK; 181 Department of Diabetes & Endocrinology, Imperial College Healthcare NHS Trust, London, UK; 182 Department of Diabetology, Madras Diabetes Research Foundation & Dr. Mohan’s Diabetes Specialities Centre, Chennai, India; 183 Department of Medicine, Faculty of Medicine and Health Sciences, University of Auckland, Auckland, New Zealand; 184 Auckland Diabetes Centre, Te Whatu Ora Health New Zealand, Auckland, New Zealand; 185 Medical Bariatric Service, Te Whatu Ora Counties, Health New Zealand, Auckland, New Zealand; 186 Oxford NIHR Biomedical Research Centre, University of Oxford, Oxford, UK; 187 University of Cambridge, Metabolic Research Laboratories and MRC Metabolic Diseases Unit, Wellcome-MRC Institute of Metabolic Science, Cambridge, UK; 188 Department of Epidemiology & Public Health, University of Maryland School of Medicine, Baltimore, MD USA; 189 Department of Internal Medicine, Division of Metabolism, Endocrinology and Diabetes, University of Michigan, Ann Arbor, MI USA; 190 AdventHealth Translational Research Institute, Orlando, FL USA; 191 Pennington Biomedical Research Center, Baton Rouge, LA USA; 192 MRC Human Genetics Unit, Institute of Genetics and Cancer, University of Edinburgh, Edinburgh, UK; 193 Yale School of Medicine, New Haven, CT USA; 194 Faculty of Medicine and Health, University of Sydney, Sydney, NSW Australia; 195 Department of Endocrinology, Royal Prince Alfred Hospital, Sydney, NSW Australia; 196 Kaiser Permanente Northwest, Kaiser Permanente Center for Health Research, Portland, OR USA; 197 Clinial Research, Steno Diabetes Center Copenhagen, Herlev, Denmark; 198 Department of Clinical Medicine, Faculty of Health and Medical Sciences, University of Copenhagen, Copenhagen, Denmark; 199 Department of Endocrinology and Diabetology, University Hospital Düsseldorf, Heinrich Heine University Düsseldorf, Moorenstr. 5, 40225 Düsseldorf, Germany

**Keywords:** Prognostic markers, Diabetes complications

## Abstract

**Background:**

Precision medicine has the potential to improve cardiovascular disease (CVD) risk prediction in individuals with Type 2 diabetes (T2D).

**Methods:**

We conducted a systematic review and meta-analysis of longitudinal studies to identify potentially novel prognostic factors that may improve CVD risk prediction in T2D. Out of 9380 studies identified, 416 studies met inclusion criteria. Outcomes were reported for 321 biomarker studies, 48 genetic marker studies, and 47 risk score/model studies.

**Results:**

Out of all evaluated biomarkers, only 13 showed improvement in prediction performance. Results of pooled meta-analyses, non-pooled analyses, and assessments of improvement in prediction performance and risk of bias, yielded the highest predictive utility for N-terminal pro b-type natriuretic peptide (NT-proBNP) (high-evidence), troponin-T (TnT) (moderate-evidence), triglyceride-glucose (TyG) index (moderate-evidence), Genetic Risk Score for Coronary Heart Disease (GRS-CHD) (moderate-evidence); moderate predictive utility for coronary computed tomography angiography (low-evidence), single-photon emission computed tomography (low-evidence), pulse wave velocity (moderate-evidence); and low predictive utility for C-reactive protein (moderate-evidence), coronary artery calcium score (low-evidence), galectin-3 (low-evidence), troponin-I (low-evidence), carotid plaque (low-evidence), and growth differentiation factor-15 (low-evidence). Risk scores showed modest discrimination, with lower performance in populations different from the original development cohort.

**Conclusions:**

Despite high interest in this topic, very few studies conducted rigorous analyses to demonstrate incremental predictive utility beyond established CVD risk factors for T2D. The most promising markers identified were NT-proBNP, TnT, TyG and GRS-CHD, with the highest strength of evidence for NT-proBNP. Further research is needed to determine their clinical utility in risk stratification and management of CVD in T2D.

## Plain language summary

People living with type 2 diabetes (T2D) are more likely to develop problems with their heart or blood circulation, known as cardiovascular disease (CVD), than people who do not have T2D. However, it can be difficult to predict which people with T2D are most likely to develop CVD. This is because current approaches, such as blood tests, do not identify all people with T2D who are at an increased risk of CVD. In this study we reviewed published papers that investigated the differences between people with T2D who experienced CVD compared to those who did not. We found some indicators that could potentially be used to determine which people with T2D are most likely to develop CVD. More studies are needed to determine how useful these are. However, they could potentially be used to enable clinicians to provide targeted advice and treatment to those people with T2D at most risk of developing CVD.

## Abstract (web summary)

Ahmad, Lim, Morieri, Tam et al perform a systematic review and meta-analysis of biomarkers, genetic markers, and risk scores for prediction of cardiovascular outcomes in Type 2 diabetes. A few prognostic markers are identified that provide incremental predictive utility beyond established cardiovascular risk factors.

## Introduction

Individuals with type 2 diabetes (T2D) have a 1.5 to 2-fold higher risk of developing cardiovascular disease (CVD) compared to those without T2D^1,2^. This is particularly concerning given the high global prevalence of diabetes and the aging population. More than 500 million individuals worldwide are affected by this chronic disease, resulting in substantial human and economic costs^3,4^. However, predicting CVD risk in T2D remains a challenge, and existing risk algorithms, such as the UK Prospective Diabetes Study (UKPDS) Risk Engine and Framingham Risk Score (FRS), have shown only modest predictive value in external validation studies^5–7^. Thus, it is essential to identify or develop readily available and cost-effective measures that can accurately identify individuals with a higher absolute risk of developing CVD beyond the risk estimated from established risk factors.

Precision medicine provides a promising approach to optimize risk prediction by integrating multidimensional data (i.e., genetic, clinical, sociodemographic), accounting for individual differences^8^. Recognizing the potential value of precision medicine in improving diabetes prevention and care, the Precision Medicine in Diabetes Initiative (PMDI) was established in 2018 by the American Diabetes Association (ADA) in partnership with the European Association for the Study of Diabetes (EASD) and is led by global leaders in precision diabetes medicine^9^. This systematic review is written on behalf of the ADA/EASD PMDI as part of a comprehensive evidence evaluation in support of the 2nd International Consensus Report on Precision Diabetes Medicine^10^. As part of this broader initiative, we conducted a systematic review and meta-analyses addressing precision prognosis for CVD outcomes.

While previous systematic reviews of biomarkers for prediction of CVD have been conducted in the general population^11–25^, this review focused on patients with T2D. We sought to answer two questions: (1) Which novel markers predict CVD in people with T2D? (2) Is there any evidence that these markers enhance risk prediction beyond current practice? Addressing these questions may inform the development of more effective strategies for detecting and predicting CVD in individuals with T2D, ultimately leading to improved management and prevention of this complication.

Therefore, to identify those biomarkers with most promising clinical utility for CV risk assessment, we followed a rigorous stepwise approach, including evaluation of the incremental value of each biomarker beyond traditional risk factors (i.e. with evaluation of improvement in different metrics such as c-statistic and net reclassification improvement – NRI), as recommended by the statement from the American Heart Association for identification of novel markers for CV disease^26^.

In summary, employing a stringent study selection process, this systematic review and meta-analysis identified four prognostic factors with high predictive utility, supported by moderate to high-strength evidence. Furthermore, three prognostic factors demonstrated moderate predictive utility, backed by low to moderate-strength evidence, and six prognostic factors showed low predictive utility, with evidence levels ranging from low to moderate. Risk scores demonstrated modest discrimination on internal validation, with diminished performance in external validation, particularly in cohorts diverging from the original population.

## Methods

**Fig. 1:**
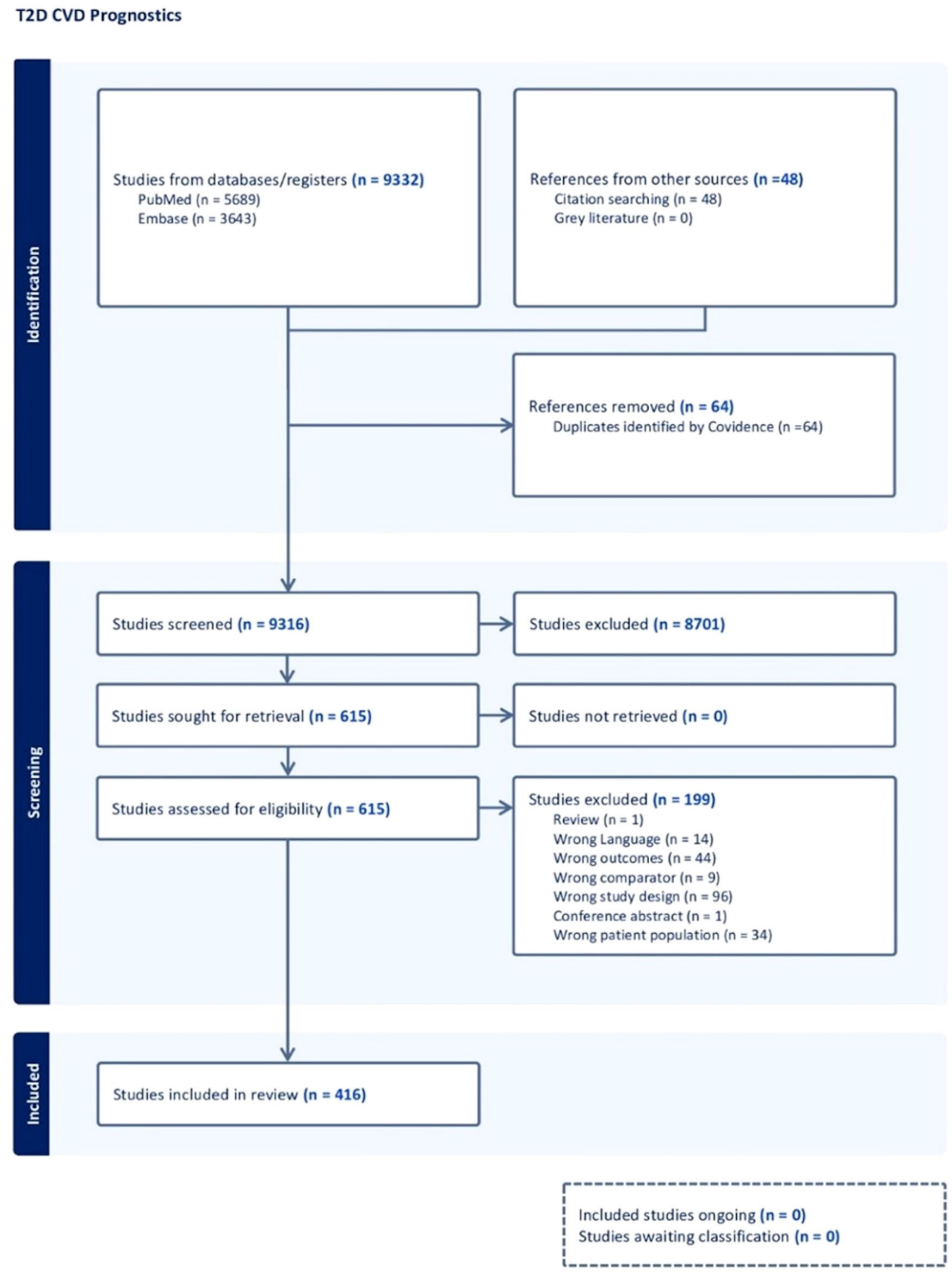
PRISMA flow diagram detailing the process that led to final study inclusion for review. Flowchart illustrating the screening of studies through title and abstract review, screening, and inclusion. *n* number of studies.

As a reporting guidance, we followed the Preferred Reporting Items for Systematic reviews and Meta-Analyses (PRISMA) statement^27^. Figure 1 presents the PRISMA flow diagram, illustrating the process that led to the final selection of studies for review. Prior to data collection, the proposed systematic review and meta-analysis was registered on PROSPERO (Registration number: CRD42021262843).

### Inclusion and exclusion criteria

This review included longitudinal studies (prospective or retrospective cohorts, including secondary analyses of cohorts from randomized controlled trials) of participants with T2D (youth-onset and adult-onset). Inclusion criteria included observational studies published from 1990–2021 that reported on the association between a prognostic factor or risk score and one or more CVD outcomes among participants with T2D. During the period of our review, the diagnostic criteria for T2D underwent some modifications (e.g. change in fasting glucose threshold, addition of hemoglobin A1C). We accepted studies that reported the inclusion of participants with T2D as defined in each individual study. Exclusion criteria included cross-sectional studies, studies utilizing surrogate endpoints for cardiovascular (CVD) outcomes such as carotid intima-media thickness, endothelial dysfunction, and arterial stiffness, and studies including only participants with pre-diabetes or only participants with type 1 diabetes. Studies with mixed populations of diabetes were included only if results were reported separately for participants with T2D. Supplemental Table 1 summarizes the Participant Intervention Comparison Outcomes and Studies (PICOS) framework.

### Outcomes

Only studies reporting outcomes on fatal or non-fatal coronary heart disease (CHD) or cardiovascular mortality (either alone or as individual component of composite outcomes) were included. A broad definition of CHD, including any outcomes defined by terms such as myocardial infarction, ischemic heart events, cardiac events, coronary artery disease, and major cardiovascular events was used.

### Search strategy

We conducted a comprehensive search on Medline and Embase of studies published from January 1990 to March 2021 using keywords and MeSH (Medical Subject Headings) terms relevant to T2D and CVD (see Supplemental Note 1). In addition, we searched the reference lists of eligible studies and systematic reviews to identify any further relevant studies. The search strategy was designed by a multi-professional team of researchers with expertise in precision medicine, clinical diabetes, cardiovascular disease, biomarker development and evaluation, genetic markers, and predictive analytics, supported by two librarians with expertise in conducting systematic reviews and meta-analyses. References identified were exported to EndNote (Clarivate Analytics) and imported to Covidence, where studies were assessed for eligibility. After the removal of duplicates, 14 authors participated in screening each title/abstract, and full-text articles were obtained if abstracts were considered eligible by at least one author. Each full-text article was assessed for inclusion independently by two authors (among 12 total authors), and disagreements were resolved by consensus.

### Data extraction

All data were extracted and coded by one author and reviewed by a second author to ensure data accuracy. After undergoing training to ensure consistency in the process, thirteen authors participated in the data extraction process (A.A., C.T., L.L., M.F.G., M.L.M., N.M., R.C.W.M., S.K., C.H., G.Y., Y.Z., M.D.P., S.C.T.). To minimize inter-reviewer variability and ensure consistency in data extraction, all authors underwent training sessions via video conferences and participated in mock assessments.

During data extraction, studies were classified into three categories based on the primary type of prognostic factors reported, namely biomarkers, genetic markers, and risk scores. Biomarkers were broadly defined as non-genetic laboratory tests, clinical conditions, socio-demographics, vital signs, diagnostic procedures, and imaging tests. Genetic markers included specific DNA sequences or variations, such as single nucleotide polymorphisms (SNPs), restriction fragment length polymorphisms (RFLP), or short tandem repeats (STR). Risk scores were defined as predictive models, algorithms, or risk calculation tools that estimated the overall likelihood or category of cardiovascular disease (CVD) based on a set of risk factors. When multiple genetic variants were combined to predict risk (using SNPs), the study was classified as a genetic marker (i.e., genetic risk score) rather than a risk score. Additional details about the included studies can be found in Supplemental Note 2.

The following data were extracted from each article using a standardized data form in Covidence and Excel data tables: study characteristics (country or countries of the study population, study start and end year, study design, inclusion/exclusion criteria, study setting, data sources), participant characteristics (years of follow-up, follow-up duration, total number of participants, race/ethnicity/ancestry, and baseline characteristics), prognostic factor(s) characteristics (name, prognostic factor type, units of measurement, units and cut-offs in regression analyses, transformation methods, effect measures [hazard ratio, odds ratio, c-statistic, net reclassification improvement (NRI), integrated discrimination index (IDI), etc.] and 95% confidence intervals, adjusted covariates), and outcomes (CVD outcome definition, number of events and non-events), and validation methods. For genetic markers, we collected risk variants, risk alleles, and closest gene (locus).

For continuous variables, we collected mean and standard deviation or median and interquartile range as reported in the study. We collected fully adjusted effect measures (HR, RR, OR, c-statistic) and their corresponding 95% CIs reported in the original articles. When studies reported multiple multivariate-adjusted effect measures, we collected the estimate from the most fully adjusted model. We did not contact primary authors to obtain data that were not reported. Furthermore, data were collected to evaluate the risk of biases in each study as summarized in Supplemental Table 2 and described in the quality assessment paragraph.

### Quality assessment

We used a modified Newcastle-Ottawa Scale (NOS) to assess quality and risk of biases. The scale assesses studies based on six common domains, including representativeness of the exposed and non-exposed cohorts, ascertainment of exposure and outcome, and adequacy of study follow-up for primary and secondary CVD events, as well as the adequacy of cohort follow-up^28^. For biomarker studies, we added two additional domains to the NOS to address bias due to confounding by evaluating the number of covariates and established CVD risk factors included in the adjusted models. Each study was given a score for each domain and an overall quality evaluation was determined by adding up these scores. The possible range of scores for non-genetic biomarkers, based on 8 domains, was 2 to 28, while for genetic biomarkers and risk scores, scores ranged from 2 to 18 based on 6 domains. Two authors assessed study quality independently, and a third author resolved any disagreements.

We reported the overall risk of bias based on the distribution of scores in each prognostic factor category, with higher scores representing lower risk of bias. Studies in the top, second, and lowest tertiles (according to the distribution specific for each type of study, i.e. non-genetic biomarkers, genetic biomarkers and risk scores) were considered to have low, medium, and high risk of bias, respectively. The score of each domain was also classified as low, medium, or high risk of bias for graphical purposes, as clarified in Supplemental Table 2.

### Statistical analysis

A random-effects model was used to pool the overall effect estimates in all meta-analyses, only if the heterogeneity test was statistically significant. For studies reporting the same effect measure (e.g. HR), we calculated the pooled effect estimate with 95% CIs for each biomarker or genetic marker and assessed heterogeneity between studies using the Cochran’s *Q* statistic (*p*  < 0.1), the *I*^2^ index >75%, and *τ*^2^. Due to the limited number of studies per prognostic factor, subgroup analyses by population characteristics or outcomes were not performed. We performed sensitivity analyses by excluding studies with high risk of bias. As the number of studies per prognostic factor was always less than 10, we were unable to assess publication bias using funnel plots. We used R, version 4.2.3 (R Project for Statistical Computing), with the “meta”, “metafor”, and “forestplot” packages for all analyses^29^. Two-sided statistical tests were used with a significance threshold of <0.05.

### Strength of the evidence

We considered aspects of the GRADE approach^30^ and the JBI critical appraisal tools^31^ in grading the strength of evidence for individual biomarkers and genetic markers/risk scores. We applied relevant GRADE criteria, including indirectness, inconsistency, and imprecision, throughout the study. Since we only included studies that involved patients with T2D and a “hard” clinical CVD outcome, the evidence is considered direct by definition. We analyzed the results from T2D patients with and without baseline CVD and specified all relevant CVD outcomes to assess the applicability of individual biomarkers in specific populations and outcomes. To ensure robustness and validity of our findings, we established strict eligibility criteria, excluding studies that did not adjust for established CVD risk factors (listed in Supplemental Table 3). Furthermore, we scored studies based on the adequacy of adjustment for covariates, including the total number of covariates and established CVD risk factors, in accordance with the JBI criterion for statistical adjustment of confounders.

We used the American Heart Association scientific consensus report for stepwise evaluation of novel markers for CVD risk^26^ to identify promising biomarkers and genetic markers based on their strength of evidence progressing from measures of association, discrimination, improvement in discrimination, net reclassification index (NRI) or integrated discrimination index (IDI). This approach is summarized in Supplemental Table 4. For biomarkers and genetic markers, we progressed from those with significant adjusted association in at least one study to those with net positive number of studies showing significant association in a consistent direction. The net positive number of studies was calculated by summing up all studies with positive association and subtracting studies with no association (e.g., three studies showing positive association and two studies with no association yielded a net positive number of one). We identified biomarkers that improved prediction performance when added to established models, based on improvement in at least one of c-statistic, NRI (the probability that a person is appropriately classified into either high- or low-risk), or IDI (quantification of predicted probabilities of events and non-events based on inclusion of the biomarker in the model), and further narrowed down the list to those with improvement in all three indicators.

Accordingly, for each of the prognostic factors that passed our evidence-based screening criteria, predictive utility was classified as high (3 points), moderate (2 points), or low (<2 points) based on three criteria: number of studies with all three performance indicators satisfied (1 point if >0 studies, 0 points if 0 studies), number of pooled meta-analyses showing significant association (1 point if >0, 0 points if 0 studies), non-pooled analysis showing $\ge$75% of studies had a significant association (1 point if yes, 0 points if no). Strength of Evidence was classified as high (4 points), moderate (2 or 3 points), or low (<2 points) based on four criteria: at least one meta-analysis was conducted regardless of outcome (1 point if yes, 0 points if no), exclusion of high risk of bias studies did not alter inferences from meta-analyses (1 point if unaltered, 0 points if altered), exclusion of high risk of bias studies did not alter inferences from non-pooled analyses (1 point if unaltered, 0 points if altered), and consistencies in the definition of the prognostic marker used in analyses (1 point if yes, 0 points if no).

For the risk scores, we provide a complete assessment of risk of bias and pooled c-statistics; however, we decided not to conduct a corresponding stepwise approach to evidence grading as explained above for biomarkers/genetic markers due to the complexity in verifying specifications of each model over time and across comparisons. Inferences from the risk score results are here meant to guide future work that would permit analyses to handle this complexity.

### Inclusion and ethics statement

This research is a part of a broader initiative, Precision Medicine in Diabetes Initiative (PMDI), that was established by the American Diabetes Association (ADA) in partnership with the European Association for the Study of Diabetes (EASD) and is led by global leaders in precision diabetes medicine. Therefore, researchers from multiple countries and continents have contributed to this study. The roles and responsibilities of co-authors were collaboratively agreed upon before the start of the review process. This study is exempt from ethical review due to the use of publicly available data.

### Reporting summary

Further information on research design is available in the Nature Portfolio Reporting Summary linked to this article.

## Results

### Study selection and characteristics

**Fig. 2:**
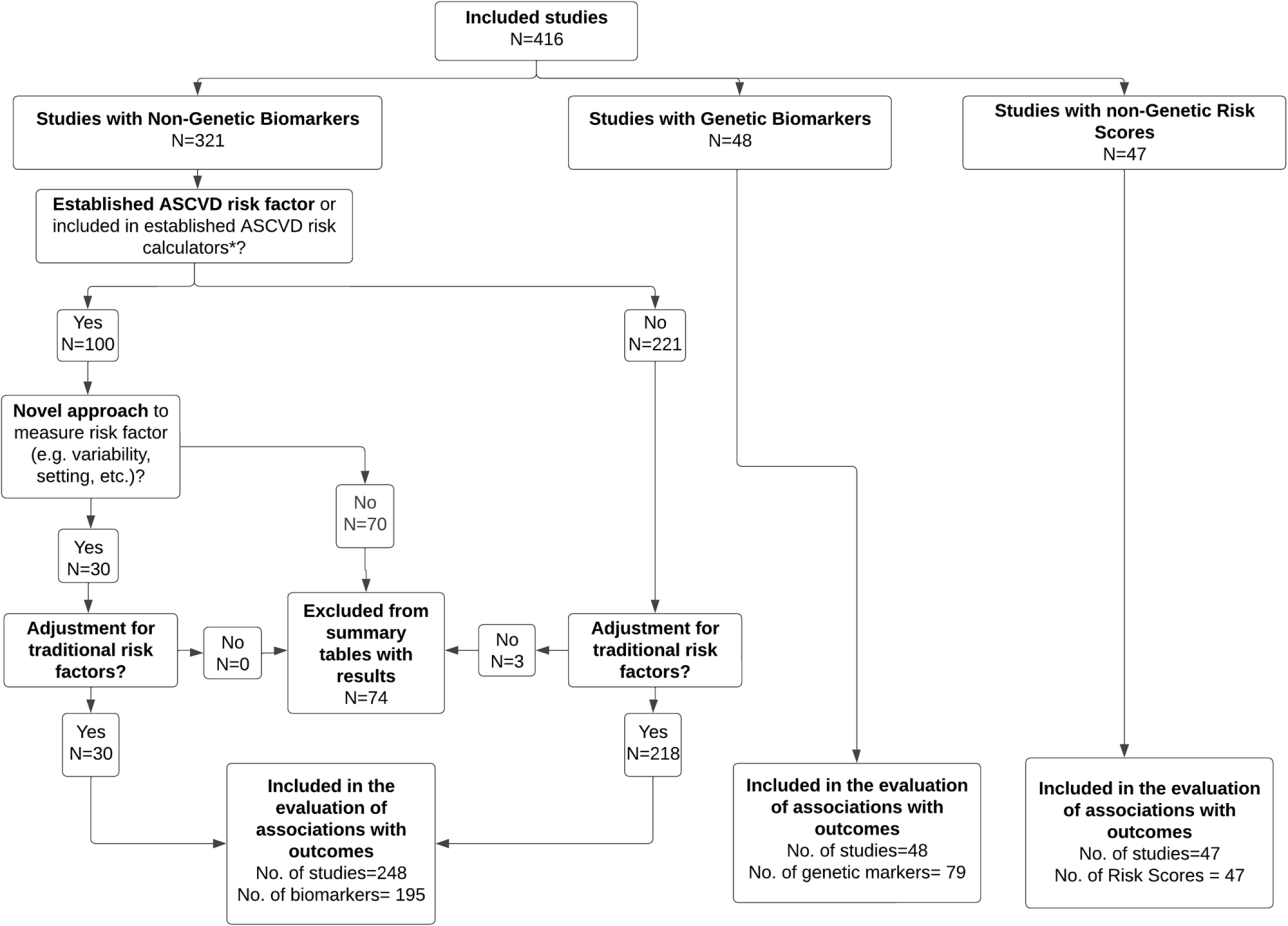
Selection of studies to be included for evaluating the associations of biomarkers, genetic markers and non-genetic risk scores with cardiovascular outcomes. This figure shows the selection criteria used to identify included biomarkers, genetic biomarkers, and non-genetic risk scores. No. number. *See Supplemental Table 3.

Out of 9380 studies identified from databases/registries (*N* = 9332) and other sources (*N* = 48), there were 9316 unique studies after removing 64 duplicates. Of these, 615 articles were selected for full-text review, and finally, 416 articles were considered appropriate for inclusion in the analysis^5,32–446^. Outcomes were reported for 321 biomarker studies, 48 genetic marker studies, and 47 risk score/model studies, as shown in Supplemental Data 1, 2, and 3. Figures 1 and 2 provide an overview of the screening and selection process.

**Fig. 3:**
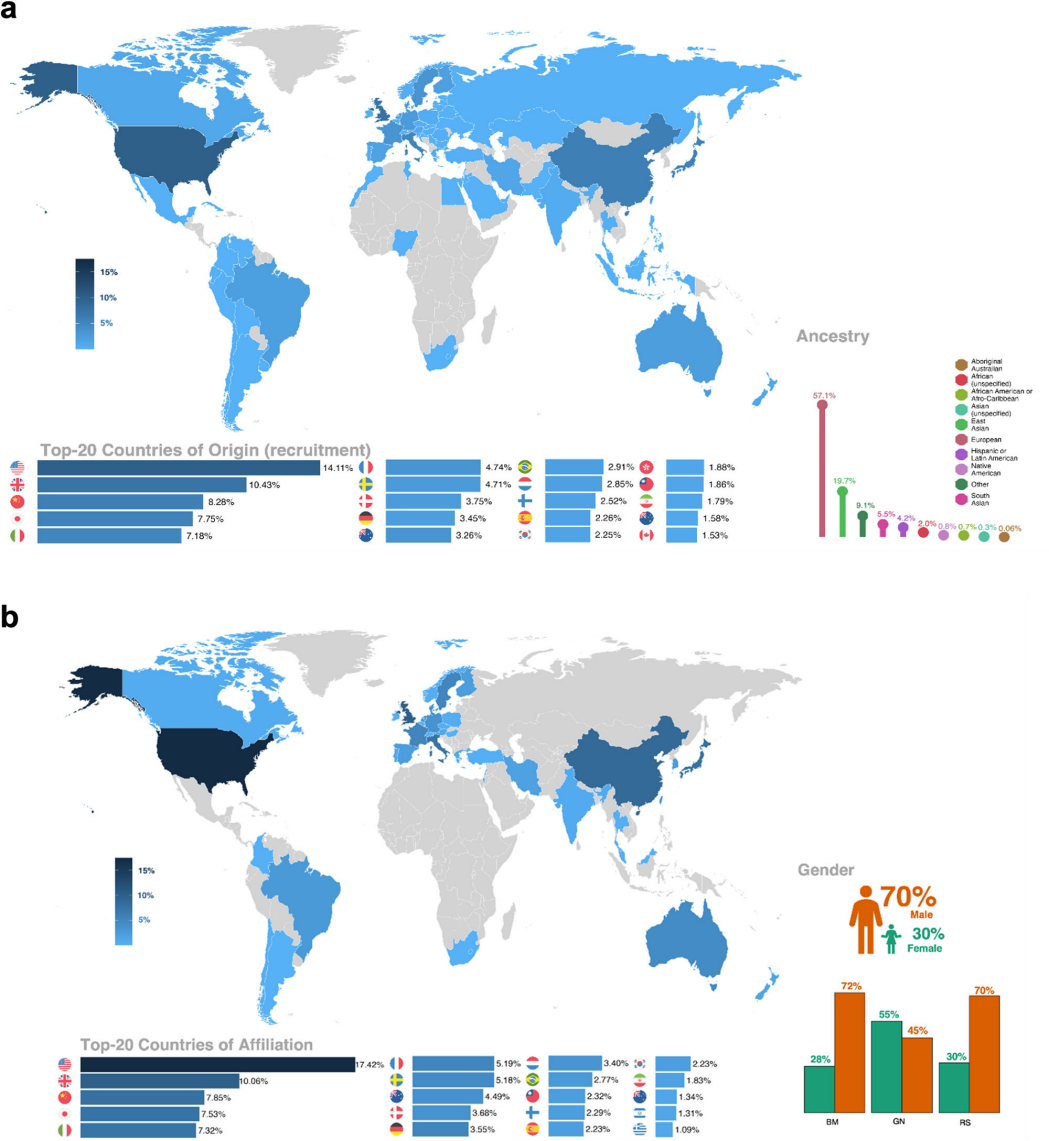
Global distribution of origin and ancestry of the study populations and countries of affiliation and gender distribution of authors of the included studies. Panel **A** shows the top 20 countries of origin and ancestry of the study populations evaluated in the included studies. Panel **B** shows the top 20 countries of affiliation and gender distribution of authors of the included studies. The data used for this visualization was obtained from PubMed and PubMed Central through manual curation and by applying text mining functions developed using R software version 4.1.2. The final proportions of ancestries were calculated for each unique study and then aggregated as described in detail here^448^.

Predominant ancestry in the studied populations were European (57.1%), East Asian (19.7%), South Asian (5.5%) and Hispanic or Latin American (4.2%). Geographically, the United States, United Kingdom, China, Japan, and Italy were the top five represented countries with regards to origin of study participants and author affiliation in the included studies. Figure 3 and online interactive figures (https://hugofitipaldi.shinyapps.io/T2D_prognostic/) offer a detailed breakdown of ethnic and geographic distributions^447^.

### CVD outcomes

There was heterogeneity in the CVD outcomes evaluated across the analyzed studies (see Supplemental Fig. 1). The median duration of follow-up reported across studies was 5 years (IQR 3.1 to 7.8 years). The most frequently reported outcomes were coronary heart disease, cardiovascular mortality, and stroke, either individually or combined. The vast majority (87%) of studies had a clearly defined outcome based on ICD-10 codes, clinical documentation, or adjudication, with 9% relying on registry or record linkage, and 4% using either patient self-report or having an unclear definition. We classified primary prevention as the prediction of CVD in individuals without a history of the disease, secondary prevention as the prediction of recurrent CVD events or CVD progression in those already diagnosed with the disease, and mixed populations as a combination of both primary and secondary prevention.

### Biomarkers

Among 416 included studies, 321 (77.2%), 48 (11.5%), and 47 (11.2%) were studies of non-genetic biomarkers, genetic biomarkers, and non-genetic risk scores, respectively. Among the 321 studies of non-genetic biomarkers, 70 (21.8%) evaluated established CVD risk factors and were excluded, while 30 studies (9.3%) were included because they used a novel approach (e.g., variability, setting) for an established risk factor (Fig. 2). Further, three studies did not adjust for any CVD risk factors and were excluded, leaving 218 studies consisting of 195 unique biomarkers in the analysis.

**Fig. 4:**
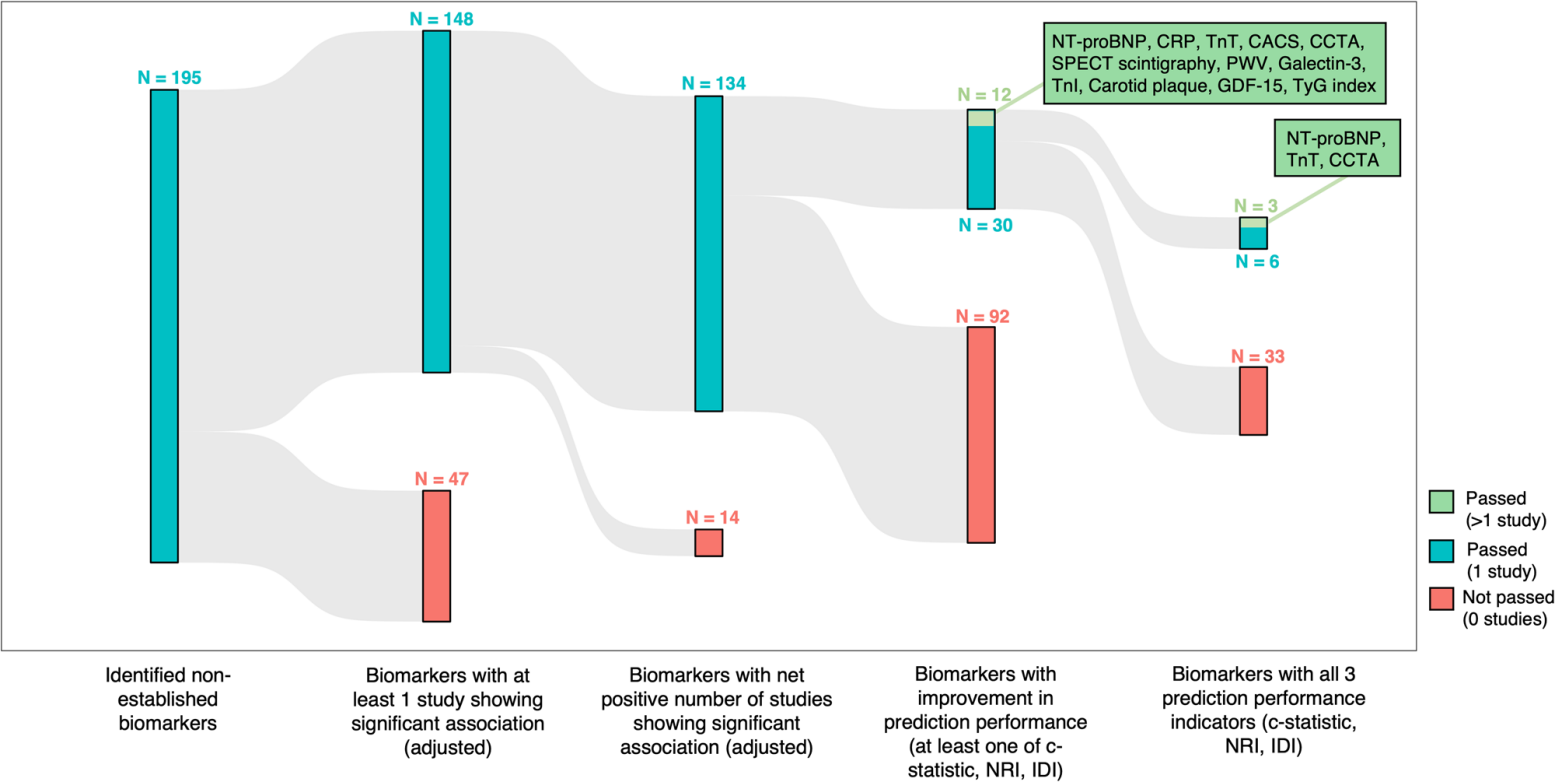
Sankey diagram showing the funneling of identified non-genetic biomarkers through sequential filtering steps. The number of biomarkers passing or not passing each step (based on the criteria specified at the bottom of the diagram) is depicted at the top of the colored bars, with biomarkers passing all steps having the strongest predictive performance value.

Among these 195 biomarkers analyzed, 134 (69%) had a significant adjusted association for predicting CVD, based on a net positive number of studies (Fig. 4 and Supplemental Data 4). Out of these, 12 (9%) showed improvement in c-statistic, NRI, or IDI in more than one study: N-terminal pro b-type natriuretic peptide (NT-proBNP), C-reactive protein (CRP), troponin T (TnT), coronary artery calcium score (CACS), coronary computed tomography angiography (CCTA), single-photon emission computed tomography (SPECT) scintigraphy, pulse wave velocity (PWV), galectin-3 (Gal-3), troponin I (TnI), carotid plaque, growth differentiation factor-15 (GDF-15), and triglyceride-glucose (TyG) index. The following biomarkers showed prediction performance but in only one study: SPECT, TnI, TyG, 25-hydroxyvitamin D, poly (ADP-ribose) polymerase (PARP), and interleukin-6 (IL-6).

Biomarkers with all three prediction performance indicators satisfied in more than one study were NT-proBNP, TnT, and CCTA, with results summarized in Table 1. For NT-proBNP, 5 studies reported improvement in c-statistics ranging from 0.01 to 0.07, significant increase in NRI ranging from 0.04 to 0.50, and significant rIDI ranging from 0.012 to 0.48 (in four studies). For TnT, 3 studies reported improvement in c-statistics ranging from 0.02 to 0.10, significant NRI ranging from 0.150 to 0.44, and rIDI ranging from 0.03 to 0.05. For CCTA, 3 studies reported improvement in c-statistics ranging from 0.08 to 0.35, with one study reporting statistically significant improvements in NRI of 0.55 and rIDI of 0.046. Of these three biomarkers, NT-proBNP showed the strongest incremental predictive value based on the magnitude of these indicators. Supplemental Data 5 shows the degree of variation in measurement methods used for each of these biomarkers.

**Table 1:** Performance of the prediction of 3 biomarkers with the most evidence.

Biomarker	Study	Clinical factors / biomarkers to be compared	Improvement in C-statistics		NRI		IDI	
			Yes /No	Estimate (95% CI) and *P*-value	NRI (95% CI)	*P*-value	IDI (95% CI)	*P*-value
NT-proBNP	Sharma 2020	Age, sex, SBP, history of HF, duration of diabetes, prior MI, hypertension, hyperlipidemia, smoking, eGFR.	Yes	0.05 (NR)	0.39 (0.30–0.47)	NR	0.09 (0.08–0.10)	<0.05
	Wolsk 2017	Prior MI, BMI, NSTEMI (index event), heart rate, HbA_1c_, percutaneous coronary intervention at the index event, cerebrovascular disease, AF, prior HF, sodium concentration, macroalbuminuria, PAD, age, and LDL concentration.	Yes	0.01 (CI NR), *P* < 0.05	0.11 (5.7–16.6)	<0.05	0.08 (0.03–1.6)	<0.05
	Wong 2019	Age ≥ 65 years, Male, T2D, Hypertension	Yes	0.03 (CI NR), *P* = 0.001	0.35 (0.24–0.45)	<0.001	0.01 (0.01–0.02)	<0.001
	Scirica 2016	Treatment arms, age, SBP, sex, history of HF-, duration of diabetes, prior MI, history of hypertension, history of hyperlipidemia, smoking, and eGFR	Yes	0.07 (CI NR), *P* < 0.001	0.040 (0.03–0.04)	<0.05	0.48 (0.41–0.55)	<0.05
	Van der Leeuw 2016	Female sex, age at diabetes diagnosis, duration of diabetes, HbA_1c_, square of HbA_1c_, SBP, square of SBP, TC/HDL ratio, urinary ACR, current smoking status, history of major macrovascular disease	Yes	0.02 (0.00–0.04), *P*-value NR	0.2 (0.10–0.44)	<0.05	NR	NR
	Van der Leeuw 2016	Female sex, age at diabetes diagnosis, duration of diabetes, HbA_1c_, systolic blood pressure, TC/HDL ratio, eGFR, current smoking status, history of major macrovascular disease	Yes	0.02 (0.00–0.05), *P*-value NR	0.50 (0.26–0.73)	<0.05	NR	NR
TnT	Lepojarvi 2016	Age, sex, history of acute MI, BMI, Canadian Cardiovascular Society grading of angina pectoris, left ventricular ejection fraction and mass index, HDL cholesterol, ACR, HbA_1c_, and type of glucose metabolism disorder	Yes	0.10 (CI and *P-*value NR),	0.231 (0.067–0.394)	<0.01	0.05 (0.03–0.08)	<0.001
	Scirica 2016	Treatment arms, age, SBP, sex, history of HF, duration of diabetes, prior MI, history of hypertension, history of hyperlipidemia, smoking, and eGFR	Yes	0.07 (CI NR), *P* < 0.001	0.440 (0.380–0.510)	<0.05	0.03 (0.02–0.03)	<0.05
	Rørth 2019	Natural logarithm of NT-proBNP, age, sex, treatment effect, ejection fraction, NYHA class, BMI, heart rate, SBP, creatinine, LDL, prior angina pectoris, AF and pacemaker implantation.	Yes	0.02 (CI NR), *P* = 0.02	0.150 (0.051–0.261)	0.007	0.03 (0.01–0.06)	<0.001
CCTA	Lee 2017	Age, male sex, HTN, smokers, hyperlipidemia, eGFR, and HbA_1c_	Yes	0.07 (CI NR), *P* = 0.03	0.550 (0.343–0.757)	<0.0001	0.05 (0.02–0.07)	0.0006
	Halon 2016	UKPDS and log CAC Score	Yes	0.35 (CI NR), *P* = 0.021	0.63 (CI NR)	NR	0.65 (CI NR)	NR

Data on improvement in C-statistics was collected from the study, either as reported or derived by comparing the C-statistic from the reference model with the C-statistic obtained from the combination of the reference model and the novel biomarker. *NR* Not Reported, *SBP* systolic blood pressure, *HF* heart failure, *MI* myocardial infarction, *BMI* body mass index, *AF* atrial fibrillation, *PAD* peripheral artery disease, *T2D* Type 2 diabetes, *eGFR* estimated glomerular filtration rate, *ACR* albumin-creatinine ratio. *P*-values were extracted from studies as reported, therefore some *p*-values are given as <0.05 or <0.001 because exact *p*-values were unavailable.

**Fig. 5:**
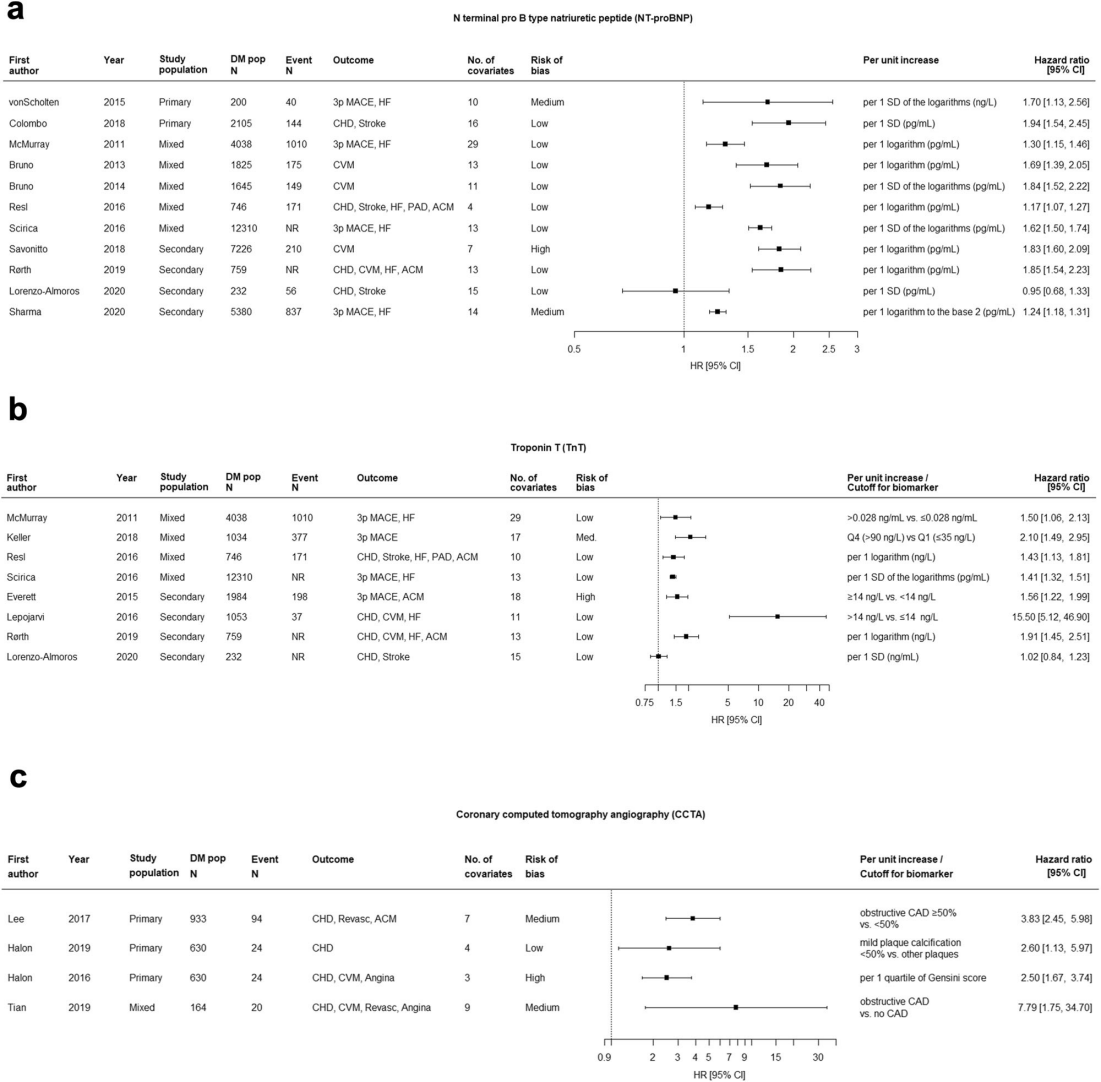
Forest plots for three biomarkers (NT-proBNP, TnT, and CCTA) with the most evidence for prediction of CVD outcomes. Panel **a** (NT-proBNP); Panel **b** (TnT); Panel **c** (CCTA). HR hazard ratio, CI confidence interval, DM pop N, sample size for diabetes population; Event N, number of individuals developed CVD outcomes; 3p MACE, 3-point major adverse cardiovascular events; HF heart failure, CHD coronary heart disease, CVM cardiovascular mortality, PAD peripheral artery disease, ACM all-cause mortality.

**Fig. 6:**
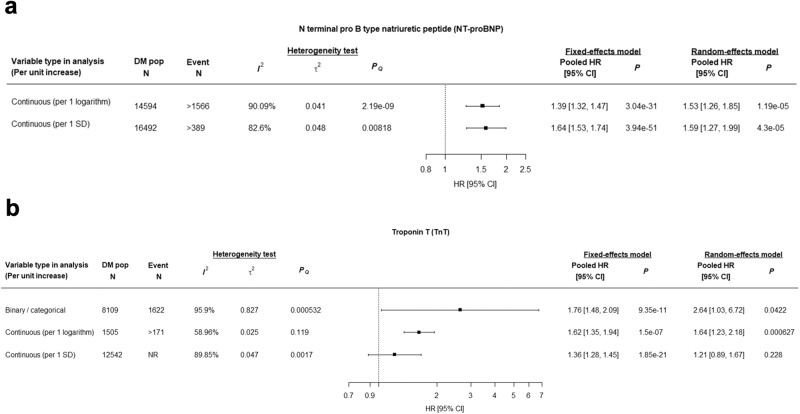
Meta-analysis of NT-proBNP and TnT for predicting cardiovascular outcomes. Panel **a** (NT-proBNP); Panel **b**: TnT; *PQ* is the *p*-value obtained from the Cochran’s Q test. HR, hazard ratio; CI, confidence interval; DM pop *N*, sample size for diabetes population; Event *N*, number of individuals developed CVD outcomes.

Forest plots in Fig. 5a show the HRs for 11 studies evaluating NT-proBNP, conducted in heterogeneous populations (2 primary, 5 mixed, and 4 secondary), outcomes, units in regression analyses (i.e., SD, SD of log), and laboratory units (ng/L, pg/mL). Nonetheless, all studies except one showed a significant association with a CVD outcome. Eight out of 11 (73%) studies were assessed to be at low risk of bias. Figure 6a and Supplemental Figs. 2a, b show the meta-analysis of NT-proBNP as a continuous variable per logarithmic and per 1 SD unit increase, confirming the highly significant association with CVD (pooled HR 1.53, 95% CI 1.26-1.85 per log increase; pooled HR 1.59, 95% CI 1.27–1.99 per SD increase) after accounting for heterogeneity with the random effects models (I^2^ 90% and I^2^ 83%, respectively). Interestingly, although our review excluded studies focusing exclusively on heart failure patients, among three studies that incorporated EF as a covariate in their models, NT-proBNP was shown to have predictive value for cardiovascular outcomes independent of EF^241,326,397^ (Supplemental Data 5).

Forest plots in Fig. 5b show the HRs for 8 studies evaluating TnT, conducted primarily for mixed or secondary populations with variable CVD outcomes. Studies differed with respect to cut-offs and categories for TnT, units of measurement (ng/ml, ng/L) and analysis (per log, per 1 SD log). Among these studies, all but one showed a positive association. Notably, the study by Lepojarvi 2016 was an outlier in its magnitude of effect and confidence intervals. Overall, for TnT, study quality was good with 6 out of 8 (75%) assessed to be at low risk of bias^227^. A significant association for TnT was observed in studies where the biomarker was evaluated as a continuous variable per 1 log increase with pooled HR 1.64 (95% CI 1.23, 2.18) and I^2^ 59% (Fig. 6b and Supplemental Fig. 3a); similarly, when treated as a binary or categorical variable, the pooled HR was 2.64 (95% CI 1.03, 6.72) with I^2^ = 95.9% (Fig. 6b and Supplemental Fig. 3b). However, when treated as a continuous variable per 1 SD, there was no longer a significant association in a random effects model (Fig. 6b and Supplemental Fig. 3c).

Forest plots in Fig. 5c show the HRs for 5 studies evaluating CCTA conducted primarily for primary CVD prevention with variable CVD outcomes. Studies differed significantly with respect to CCTA definition of subclinical or clinical CHD. All 5 studies showed a significant association; however, 2 of the 5 studies (40%) were assessed to be at a high risk of bias.

Apart from these three biomarkers, SPECT, TnI, TyG, 25-hydroxyvitamin D, poly(ADP-ribose) polymerase (PARP), and interleukin-6 (IL-6) showed prediction performance in all three performance indicators but in only one study. Forest plots for the remaining 9 biomarkers that showed improvement in at least one performance indicator in more than one study (CACS, carotid plaque, CRP, gal-3, GDF-15, PWV, SPECT scintigraphy, TnI, and TyG) are shown in Supplemental Figs. 4–6. Again, there was substantial heterogeneity with respect to study populations, outcomes, and units of analysis for these biomarkers. Biomarkers showing positive association in at least 75% of studies included CACS, carotid plaque, gal-3, PWV, SPECT scintigraphy, TnI, and TyG. While CRP did not meet the threshold of 75% of studies showing an association, when meta-analyzed as a binary or categorical variable, it showed a significant pooled association; PWV and TyG also demonstrated significant association in pooled analysis (Supplemental Fig. 7).

### Genetic markers

**Fig. 7:**
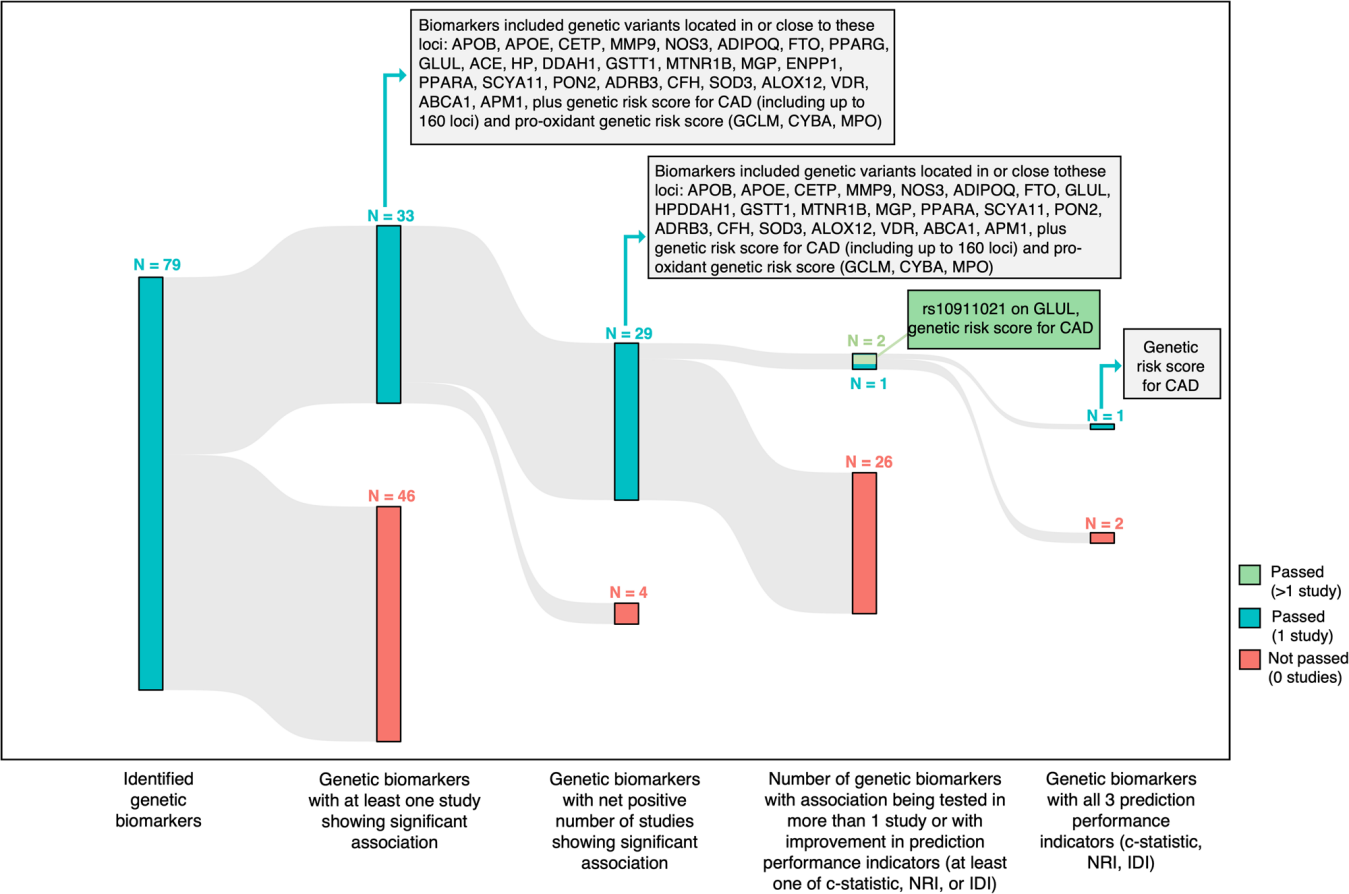
Sankey diagram showing the funneling of identified genetic biomarkers through sequential filtering steps. The number of biomarkers passing or not passing each step (based on the criteria specified at the bottom of the diagram) is depicted at the top of the colored bars, with biomarkers passing all steps having the strongest predictive performance value.

Among the 48 genetic studies analyzed (Supplemental Data 2), 79 genetic biomarkers were examined for their association with incident CVD events (Supplemental Data 6), mainly in populations of European (65%) or Asian (26%) ancestries, with sparse representation of populations of other ancestries (e.g., African 12% or Hispanic 3%), with 12% of associations being tested in mixed populations. Most of the studies (70 out of 79) used single variants as distinct genetic biomarkers (exposure), while 9 studies used a combination of different SNPs into genetic risk scores (GRS) as the exposure. Remarkably, most of these exposures were tested only in one study, and external validation was performed in only 4 out of 48 studies, with only one study using a longitudinal cohort as a validation set, i.e., GRS for CHD. Overall, among the 79 genetic biomarkers, 33 (41.8%) had at least one study showing significant association, out of which 29 had a net positive number of studies showing significant association. Out of these 29 genetic biomarkers, two were tested in more than one study (rs10911021 on *GLUL*, GRS for CHD [GRS-CHD]), one had improvement in any performance indicator in a single study (isoform e4 in *APOE*), and one had improvement in all three performance indicators in a single study (GRS-CHD) (Fig. 7).

**Fig. 8:**
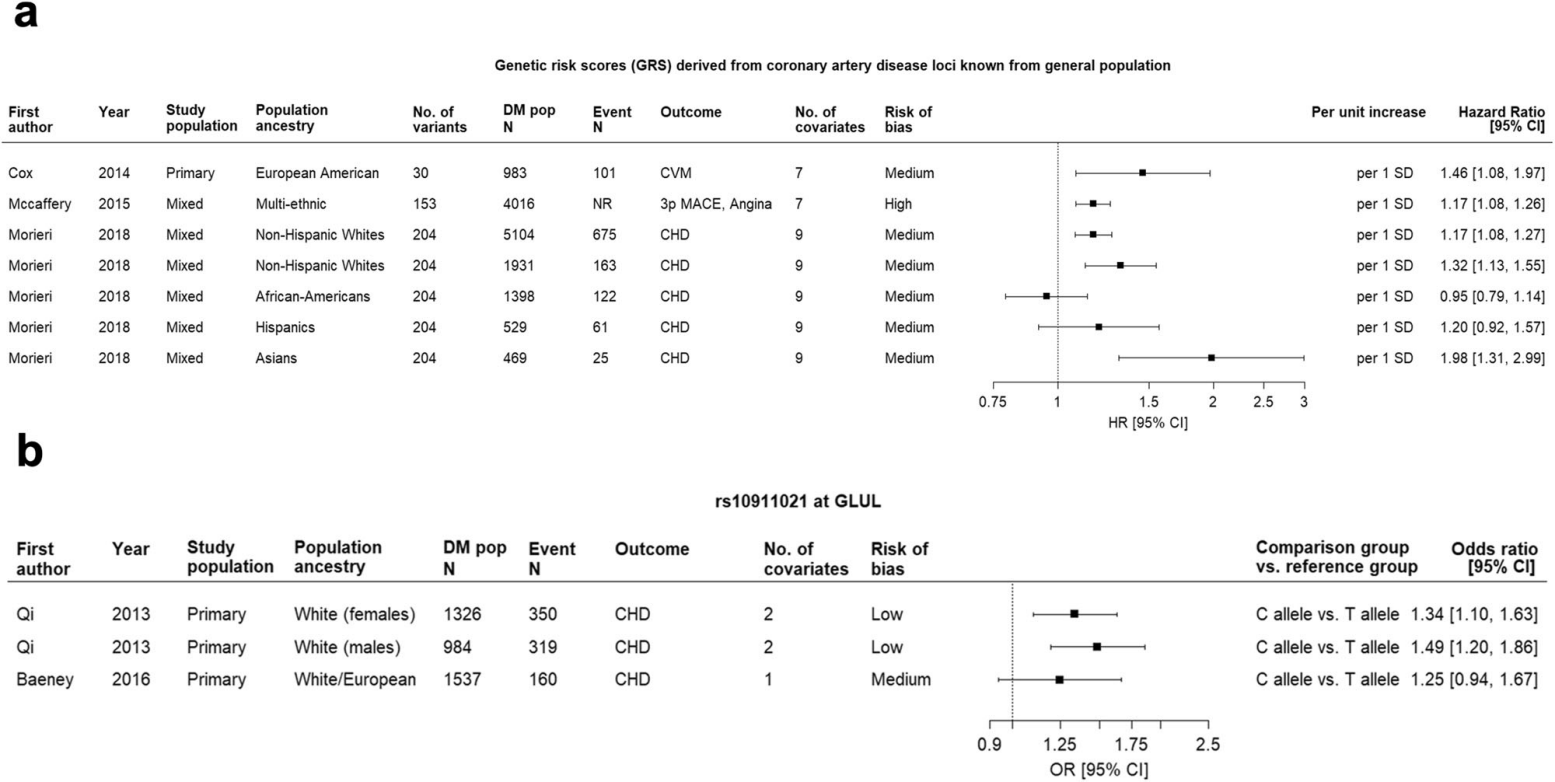
Forest plots of genetic risk scores and *GLUL* variant rs10911021 for predicting cardiovascular outcomes. Panel **a**: Genetic risk scores; Panel **b**: *GLUL* variant rs10911021; HR, hazard ratio; CI, confidence interval; DM pop *N*, sample size for diabetes population; Event N, number of individuals developed CVD outcomes; 3p MACE, 3-point major adverse cardiovascular events.

Notably, the rs10911021 variant in *GLUL* was the only single variant that showed an association with CVD in several studies. This variant was initially identified in T2D patients using a genome-wide approach and subsequently confirmed for its association with CVD in selected populations from two additional studies. For GRS-CHD, four separate studies investigated the combination of up to 204 CHD variants from 160 distinct loci derived from the general population. These studies had distinct but overlapping and increasing numbers of loci and variants tested in more recent investigations. The most recently performed GRSs were externally validated and demonstrated significant improvements in CVD risk reclassification (cNRI) as well as notable enhancements of 8% in relative IDI (rIDI). However, these findings were identified in subjects of European ancestry and ancestry-specific analyses showed consistency in Asian subjects but not in other ancestral backgrounds. Forest plots for variants located on the GRS-CHD and *GLUL* are shown in Fig. 8, while their meta-analyses can be found in Supplemental Fig. 8.

### Risk scores/models

Forty-seven studies reported results of 27 unique CVD risk scores (Supplemental Data 3 and 7). Supplemental Figs. 9 and 10 provides the c-statistics from internal and external validation analyses, respectively. On both internal and external validation, discrimination was modest. Most risk scores were developed in the United States, Europe, and East Asia and 61.1% of the internal validation studies were assessed to be at a high risk of bias. Model performance tended to decline when validated in countries that differed from the development cohort (Supplemental Fig. 11). For example, the FDS study achieved high c-statistics (>0.80) when validated in an Australian cohort, but lower ones (0.58-0.69) when tested in European countries. In line with previous studies^5–7^, discrimination for the UKPDS and FRS was generally poor on external validation. Most prediction models focused on baseline characteristics and did not account for time-varying factors that may modify CVD risk (e.g., statin, SGLT-2i, GLP-1 RA). An exception was the BRAVO risk engine, published in 2020 and validated in trials of SGLT-2i patients, showing that this risk engine effectively predicted CV health benefits through improvements in common clinical measures (e.g., A1C, SBP, and BMI)^343^.

Supplemental Figs. 12, 13 provide the pooled c-statistics from external validation studies on those risk scores for which the analysis was possible: ADVANCE, CHS, CVD-EDIC, NDR, NZ DCS and UKPDS risk scores. All risk scores exhibited modest discrimination (pooled c-statistics ranging from 0.63 to 0.68), with no individual risk score substantially outperforming the others.

Supplemental Fig. 14a, b provide a histogram of the total number of adjusted covariates and number of adjusted traditional CVD risk factors in each of the studies, respectively. Supplemental Fig. 15 is a network figure representing the connections of the adjusted covariates in the 416 included studies.

### Sensitivity analyses

The results of sensitivity analyses excluding studies with high risk of bias from meta-analyses of biomarkers, genetic risk score, and for risk scores where pooled analyses were possible, respectively, are shown in Supplemental Figs. 16–18.

### Synthesis

Table 2 provides a summary of findings of studies assessing the most promising biomarkers and genetic markers/scores for precision prognosis of CVD in T2D, along with our conclusions regarding their predictive utility and strength of evidence. In our synthesis of the evidence, we took into account the results from the sensitivity analyses described in the previous paragraph. The highest predictive utility was observed for NT-proBNP (high-evidence), TnT (moderate-evidence), TyG (high-evidence), and GRS-CHD (moderate-evidence). Prognostic factors with moderate predictive utility were CCTA (low-evidence), SPECT scintigraphy (low-evidence), and PWV (moderate-evidence). Prognostic factors with low predictive utility included CRP (moderate-evidence), CACS (low-evidence), Gal-3 (low-evidence), TnI (low-evidence), carotid plaque (low-evidence), and GDF-15 (low-evidence). Supplemental Figs. 19–22, 23, 24 provide the quality assessment for the included biomarker, genetic marker, and risk score studies, respectively.

**Table 2:** Conclusion and strength of the evidence.

	A	B	C	D	E	F	G	H
Prognostic Biomarker	No. of studies with all 3 performance indicators satisfied	No. of pooled meta-analyses showing significant association	No. of pooled meta-analyses showing significant association (excluding high risk of bias)	Non-pooled analyses showed that >=75% of studies had significant association	Persistent association on sensitivity analysis for non-pooled analyses (excluding high risk of bias)	Consistency in definition of prognostic biomarker used in analysis	Conclusion (predictive utility)	Strength of Evidence
NT-proBNP	3	2/2	2/2	Yes	Yes	Yes	High	High
CRP	0	1/2	1/2	No	No	No	Low	Moderate
TnT	4	2/3	1/3	Yes	Yes	No	High	Moderate
CACS	0	0	0	Yes	No	No	Low	Low
CCTA	2	0	0	Yes	No	No	Moderate	Low
SPECT	1	0	0	Yes	No	No	Moderate	Low
PWV	0	1/1	1/1	Yes	Yes	No	Moderate	Moderate
Gal-3	0	0	0	Yes	Yes	No	Low	Low
TnI	1	0	0	No	No	No	Low	Low
Carotid plaque	0	0	0	Yes	Yes	No	Low	Low
GDF-15	0	0	0	No	No	No	Low	Low
TyG	1	1/1	NA	Yes	No	Yes	High	Moderate
GRS-CHD	1	1/1	1/1	Yes	No	No	High	Moderate

Table includes genetic and non-genetic biomarkers showing improvement in prediction performance (i.e. at least one of c-statistic, NRI, IDI) and in more than 1 study (corresponding to all markers in green boxes in Fig. 4 and Fig. 5). Notes: Predictive utility was classified as high (3 point) moderate (2 points) or low (<2 points) based on the criteria defined in columns A, B and D (Column A: >0 = 1 point; Column B: >0 = 1 point, Column D: “Yes” = 1 point). Strength of Evidence was classified as High (4 points), moderate (2 or 3 points) and low (<2 points) based on criteria defined in columns B, C, E and F (Column B: at least one meta-analysis conducted [regardless of outcome] = 1 point; Column C: exclusion of high risk of bias studies did not alter inferences from meta-analyses [same number as in column B] = 1 point; Column E: exclusion of high risk of bias studies did not alter inferences from non-pooled analyses [YES] = 1 point; Column F: “yes” = 1 point).

## Discussion

Our systematic review of prognostic markers for CVD in individuals with T2D has revealed several notable findings. First, among the numerous studies that investigated the prognostic significance of CVD risk markers, only a few have been consistently found to be significantly associated with cardiovascular risk. Namely, NT-proBNP, TnT, TyG, and GRS-CHD demonstrated the highest predictive utility, with NT-proBNP having the strongest evidence. However, most of the remaining markers have not been adequately tested or compared against established CVD risk factors. Finally, even though some markers have demonstrated the capability of predicting cardiovascular events beyond what current risk factor-based models can offer, their application in clinical practice remains limited, as there is inadequate evidence of their contemporary clinical utility.

During the search process, a considerable number of studies were found ineligible for inclusion in our systematic review. Available studies were primarily cross-sectional in design, and only a limited number of them focused specifically on individuals with T2D and examined the early utility of risk factors and biomarkers in predicting future cardiovascular events. A major limitation in many studies was inadequate adjustment for established CVD risk factors; and even if studies considered adjustments, only a small fraction evaluated clinical utility beyond the use of established risk factors. These findings emphasize the need for better-designed studies to improve our understanding of the prognostic value of markers for CVD in T2D.

Most studies included in the final analysis were conducted in people of European, East or South Asian ancestry, with the top-5 countries of recruitment being the United States, UK, China, Japan and Italy. African ancestry and countries were underrepresented. A skewed geographical distribution was also evident regarding countries of author affiliation, with the same top-5 countries dominating the volume of publications. Although the geographical and ancestral imbalance reported here for biomarker studies is less pronounced than what was recently reported for GWAS studies^448^, it highlights the pressing need to enhance data collection, biomarker discovery and validation, as well as the development of population-specific cardiovascular risk prediction models in underrepresented populations and ancestries to hopefully help reduce healthcare disparities^449^.

In our analyses, the novel biomarker emerging as the best predictor was NT-proBNP; indeed, it fulfilled all criteria of predictive and clinical utility with multiple studies showing improvement in all prediction performance indicators, with consistency of results across studies and meta-analyses. Notably, this biomarker had also been found to be useful as a prognostic marker for incident CVD in the general population^450^. Our findings suggest that NT-proBNP, beyond its established role in the diagnosis and management of patients with heart failure, might also be used as a marker to predict CVD. Another biomarker found in the general population to improve primary CVD risk prediction among asymptomatic middle-aged adults is high-sensitivity CRP (hs-CRP). In our review, CRP was found to have low predictive utility with moderate strength of evidence, which may be due to variability in cut-offs used for this marker, the relatively small numbers of studies, differential effects in diabetes, or less sensitive to detect low-grade vascular inflammation (compared with hs-CRP).

Despite numerous genetic studies probing the link between polymorphisms and cardiovascular outcomes in diabetes, few genetic markers have been consistently examined in longitudinal studies or reliably found to be associated with these outcomes. Only one study from the systematic review utilized a genome-wide association study (GWAS) approach, identifying the rs10911021 variant near *GLUL* to be associated with CV outcome in diabetes, at genome-wide significance. The variant at *GLUL* was subsequently confirmed in two independent studies^172^. A more recent GWAS conducted among Chinese patients with T2D identified a variant at *PDE1A* for CHD in T2D, which was not included in our systematic review as it fell beyond our study inclusion period^451^. Polygenic risk scores also appear to emerge as promising tools, and GRS constructed from variants associated with CHD in the general population seem helpful for cardiovascular risk stratification in diabetes^257^.

Based on these limited findings, it becomes clear that we need a greater number of adequately powered GWAS to identify genetic markers associated with CVD in T2D. Nevertheless, we found several examples of studies that evaluated the utility of applying polygenic risk scores, or genome-wide polygenic risk scores, derived from the general population, for CVD risk stratification in T2D. In general, these have fair performance and a similar ability to stratify as in patients without diabetes. Considering the substantially larger sample sizes in currently published meta-analyses of GWAS for CHD in the general population, this approach will probably be more fruitful for the integration of genetic markers into risk stratification of cardiovascular complications. In the limited studies that have evaluated the added benefit of polygenic risk scores above clinical markers, there is, in general, a modest but significant improvement in prediction. Whether polygenic risk scores will become viable options for future risk stratification would partly depend on the availability of these tools, and the cost-effectiveness of adding these measures into clinical practice.

Beyond individual prognostic markers, our review identified several studies that evaluated CVD risk prediction models. While the UKPDS risk engine (developed among subjects with newly diagnosed T2D the UK) and the Framingham risk equation (developed from the general population in the US) were the most widely studied, they do not perform well in contemporary studies of people with T2D. This suggests difficulties in applying certain risk models to current healthcare settings. Nevertheless, our literature review shows that clinical risk models are perhaps the “readiest” for implementation in clinical practice to improve risk stratification in diabetes. On external validation, newer risk scores generally achieved higher discrimination compared to UKPDS and FRS, with Fremantle Diabetes Study 2 (FDS-2) having the highest c-statistic of 0.81 (developed and validated in different populations in Australia). We found that risk models performed better when validated in cohorts similar to the derivation cohort, with c-statistics of 0.699 $\pm$ 0.015 and 0.668 $\pm$ 0.006 (95% CI) (*P* = 0.018) for concordant and discordant studies, respectively.

In an era when electronic medical record (EMR)-based prediction models are being increasingly used, our results suggest that researchers should focus on the development of population-specific risk models that are intended to be deployed in the same population from which they were developed since the goal should be to achieve the highest predictive accuracy rather than to find a generic model that performs modestly well in all settings. Despite their potential utility and low implementation costs, we found a paucity of evidence showing integration of risk engine calculators into clinical practice. We are aware of several notable exceptions. For example, the Joint Asia Diabetes Evaluation (JADE) program has incorporated several risk prediction algorithms derived from Asian patients with diabetes into a web-based e-health portal, together with a graphical interface and decision support^452^, and has been evaluated in different clinical settings, including in randomized clinical trials^453–456^. Many EMR systems offer quick calculations of CVD risk using the American College of Cardiology/American Heart Association (ACC/AHA) Pooled Cohort Equations based on inputs available in the patient’s record, and we recommend that future risk scores found to have high predictive accuracy be made easily accessible to clinicians within their EMR workflow.

Given the limitations and gaps that emerged from this review, we recommend that future studies follow several guidelines to improve the quality and impact of studies on precision prognostics in diabetes. First, studies attempting to identify a risk marker should be conducted in prospective or longitudinal cohorts or trials, to provide more robust and reliable data. Second, studies should have sufficient sample size and duration of follow-up (at least 3 years for primary CVD events and at least 1 year for secondary CVD events) to ensure adequate statistical power. Third, studies must adjust for a minimal set of established clinical cardiovascular risk factors, to ensure that known risk factors do not confound any observed associations. Finally, studies must attempt to explore the added utility of biomarkers by comparing against prediction using established risk factors or models, or available risk engines for cardiovascular events. This would include evaluation of the change in c-statistics after adding risk markers/biomarkers of interest but also consider including additional metrics such as NRI and IDI. We believe that if journals make these requirements mandatory when evaluating such studies, it will help ensure that research funders are made aware and future studies are best suited for informing advances in this area especially in resource-limited countries. As in any other research field, harmonization of protocols, methods, and analysis pipelines should be encouraged to allow comparisons across studies and for clinical translation.

There are several unique strengths of this work. To our knowledge, this represents one of the most comprehensive overviews of the current status of knowledge about risk stratification of cardiovascular outcomes in T2D. We included studies from 1990 onwards, to capture some of the older studies, as well as more contemporary studies. Our inclusion of “biomarkers” in the broadest term allowed us to provide an objective overview of the different approaches currently being explored for better risk stratification. Limiting the analyses to studies using longitudinal cohorts allowed us to focus on studies that would inform prognostication. Limiting analyses to “hard” cardiovascular endpoints, rather than also including surrogate endpoints such as carotid intima-medial thickness, allowed us to focus on endpoints that would be of greatest clinical relevance. However, while this approach allows us to maximize the translational approach of our analyses, future studies focused on identification of biomarkers associated with early disease-informative endpoints (i.e. subclinical markers of atherosclerosis or minor cardiovascular disease) might identify different novel biomarkers for early-stage cardiovascular complications.

Our study does have limitations. We had to omit a considerable number of cross-sectional studies due to the extensive scope of the systematic review and the explained focus on longitudinal studies. We included only English language publications. Our search terms, potentially more sensitive towards detecting studies on clinical risk factors and biomarkers than genetic factors, may have led to fewer genetic studies being identified. However, we managed to supplement this by reintegrating some missing articles using the identified literature and the investigators’ expertise.

In conclusion, our systematic review on prognostic markers for cardiovascular endpoints in T2D identified several findings, which to the best of our knowledge, have not been previously reported, and has revealed some important knowledge gaps. We found that NT-proBNP, TnT, TyG, and GRS-CHD had high predictive utility beyond traditional CVD risk factors, with the highest strength of evidence for NT-proBNP. Among genetic markers, there was only sufficient evidence for the polygenic risk score for CHD, and among risk scores, predictive utility was modest on external validation. Given the relatively low number of studies analyzing these novel prognostic factors using a rigorous approach, these findings support the need for future studies testing these markers with convincing demonstration of incremental predictive utility. NT-proBNP appears to be the only biomarker ready to be tested prospectively to evaluate its utility in modifying clinical practice for prediction of CVD risk.

### Supplementary information

Supplemental Material

Description of Additional Supplementary Files

Supplemental Data 1

Supplemental Data 2

Supplemental Data 3

Supplemental Data 4

Supplemental Data 5

Supplemental Data 6

Supplemental Data 7

Reporting summary

## Data Availability

The protocol for this systematic review and meta-analysis is publicly available through the International Prospective Register of Systematic Reviews (PROSPERO), with the registration number CRD42021262843. Comprehensive search strategies that can be reproduced are outlined in Supplemental Note 1. Any further details required are available from the corresponding author upon reasonable request. Complete lists of the publications where data were extracted for this study are provided as Excel files in Supplemental Data 1 (list of studies on non-genetic biomarkers), Supplemental Data 2 (list of studies on genetic biomarkers), and Supplemental Data 3 (list of studies on non-genetic risk scores). Supplemental Data 4, 6, and 7 provide source data used to generate forest plots and meta-analyses. The data presented in Fig. 3 is also available as online interactive figures (https://hugofitipaldi.shinyapps.io/T2D_prognostic/) and in a data repository (https://zenodo.org/records/10277173)^447^.
