# Supplementary material for: Precision prognostics for cardiovascular disease in Type 2 diabetes: a systematic review and meta-analysis
Source: Commun Med (Lond). 2024 Jan 22;4:11. doi: 10.1038/s43856-023-00429-z (PMC10803333; doi:10.1038/s43856-023-00429-z)
Supplement: Supplementary file 1 — Supplemental Material [file 43856_2023_429_MOESM1_ESM.pdf]

Supplemental Note 1. Search strategy for the systematic review and meta-analysis

Literature search strategy

Pubmed <1990 to 2021 Week 25>

#1. "Diabetes Mellitus, Type 2"[Mesh] OR NIDDM[Title/Abstract] OR T2DM[Title/Abstract] OR T2D[Title/Abstract] OR "non insulin depend*"[Title/Abstract] OR "noninsulin depend*"[Title/Abstract] OR noninsulin-depend*[Title/Abstract] OR non-insulindepend*[Title/Abstract] OR ((type 2[Title/Abstract] OR type II[Title/Abstract] OR

type2[Title/Abstract] OR type II[Title/Abstract] OR Ketosis-Resistant[Title/Abstract]) AND

diabet*[Title/Abstract]) OR ((late[Title/Abstract] OR adult[Title/Abstract] OR stable[Title/Abstract]) AND onset[Title/Abstract] AND diabet*[Title/Abstract])

=215774

#2. prognoses[Title/Abstract] OR prognosis[Title/Abstract] OR predict*[Title/Abstract] OR

nomogram*[Title/Abstract] OR model*[Title/Abstract] OR prognosti*,[Title/Abstract] OR "survival analys*"[Title/Abstract] OR prospective[Title/Abstract] outcome[Title/Abstract] OR progression[Title/Abstract] OR efficacy[Title/Abstract] OR effectiveness[Title/Abstract] OR longitudinal[Title/Abstract] OR "time series"[Title/Abstract]

=2441175

#3. "risk score*"[Title/Abstract] OR "risk assessment*"[Title/Abstract] OR "risk categor*"[Title/Abstract] OR "risk factor*"[Title/Abstract] OR test*[Title/Abstract] OR biomarker*[Title/Abstract] OR assay*[Title/Abstract] OR tool* [Title/Abstract] OR genetic[Title/Abstract] OR genomic[Title/Abstract] OR polymorphism*[Title/Abstract] OR variant*[Title/Abstract] OR algorithm*[Title/Abstract] OR equation*[Title/Abstract] OR precision[Title/Abstract] OR personali*[Title/Abstract]

=7187775

#4. #2 AND #3

=843991

#5. #2 OR #3

=8773559

#6. ("Atherosclerosis"[Mesh]) OR (((((((("Cardiovascular Diseases"[Mesh]) OR "Myocardial Infarction"[Mesh]) OR "Myocardial Ischemia"[Mesh]) OR "Acute Coronary Syndrome"[Mesh]) OR "Coronary Artery Disease"[Mesh]) OR "Coronary Artery Bypass"[Mesh]) OR "Angioplasty"[Mesh]) OR "Coronary Occlusion"[Mesh]) OR "Percutaneous Coronary Intervention"[Mesh])

=2521345

#7. "cardiovascular disease*"[Title/Abstract] OR "myocardial infarct*"[Title/Abstract] OR "heart infarct*"[Title/Abstract] OR "heart attack*"[Title/Abstract] OR "heart disease*"[Title/Abstract] OR "atheroscleros*"[Title/Abstract] OR "atherosclerotic"[Title/Abstract] OR "myocardial ischemia"[Title/Abstract] OR "myocardial ischaemia"[Title/Abstract] OR "myocardial revascularisation"[Title/Abstract] OR "acute coronary syndrome*"[Title/Abstract] OR "coronary heart disease*"[Title/Abstract] OR "coronary artery disease*"[Title/Abstract] OR "coronary disease*"[Title/Abstract] OR "ischemic heart disease*"[Title/Abstract] OR "ischaemic heart disease*"[Title/Abstract] OR ASCVD[Title/Abstract] OR IHD[Title/Abstract] OR AMI[Title/Abstract] OR

ACS[Title/Abstract] OR CHD[Title/Abstract] OR CAD[Title/Abstract] OR angioplast*[Title/Abstract] OR "coronary artery bypass*"[Title/Abstract] OR "chronic total occlusion*"[Title/Abstract] OR "chronic total coronary occlusion*"[Title/Abstract] OR CTO[Title/Abstract] OR "Percutaneous Coronary Intervention*"[Title/Abstract] OR "Percutaneous Coronary Revascularization"[Title/Abstract] OR "Percutaneous Coronary Revascularisation"[Title/Abstract] OR "Percutaneous coronary angioplasty"[Title/Abstract] OR "PCI"[Title/Abstract] OR "major adverse cardiovascular event"[Title/Abstract] OR "major adverse cardiac events"[Title/Abstract] OR MACE[Title/Abstract]

=851592

#8. #6 OR #7

=2767282

#9. #1 AND #4 AND #8

=4851

#10. #9 Filters, Humans, 1990-2021

=4192

#11. #1 AND #5 AND #8

=32616

#12. #11 Filters, Humans, 1990-2021

=28170

References article: 12502653

Retrieved in #12, not in #10

#13. prognoses[Title/Abstract] OR prognosis[Title/Abstract] OR predict*[Title/Abstract] OR

nomogram*[Title/Abstract] OR model*[Title/Abstract] OR prognosti*,[Title/Abstract] OR "survival analys*"[Title/Abstract] OR prospective[Title/Abstract] outcome[Title/Abstract] OR progression[Title/Abstract] OR efficacy[Title/Abstract] OR effectiveness[Title/Abstract] OR longitudinal[Title/Abstract] OR "time series"[Title/Abstract] OR predictor[Title/Abstract] OR predictors[Title/Abstract]

=2722451

#14. #3 AND #13

=972873

#15. #1 AND #8 AND #14 Filters, Humans, From 1990-2021

=5689 references

Embase <1990 to 2021 Week 26>

#1. 'non insulin dependent diabetes mellitus'/exp OR niddm:ab,ti OR t2dm:ab,ti OR t2d:ab,ti OR 'noninsulin dependent':ab,ti OR 'non insulin dependent':ab,ti OR 'type 2':ab,ti OR 'type ii':ab,ti OR type2:ab,ti OR typeii:ab,ti OR 'ketosis resistant':ab,ti OR (diabet*:ab,ti AND onset:ab,ti AND (late:ab,ti OR adult:ab,ti OR stabl*:ab,ti))

526627

#2. (biomarker*:ab,ti OR test*:ab,ti OR assay*:ab,ti OR tool*:ab,ti OR genetic:ab,ti OR genomic:ab,ti OR polymorphism*:ab,ti OR variant*:ab,ti OR 'genetic risk score*':ab,ti OR 'polygenic risk score*':ab,ti) AND (prognos*:ab,ti OR predict*:ab,ti OR model*:ab,ti OR nomogram*:ab,ti OR 'survival analysis':ab,ti OR

efficacy:ab,ti OR effectiveness:ab,ti OR prospective:ab,ti OR longitudinal:ab,ti OR 'time series':ab,ti OR statistical:ab,ti OR score*:ab,ti OR risk*:ab,ti OR 'risk score':ab,ti OR 'risk categor*':ab,ti OR 'risk factor*':ab,ti OR 'risk assessment':ab,ti OR algorithm*:ab,ti OR equation*:ab,ti OR precision:ab,ti OR personali*:ab,ti OR outcome*:ab,ti OR progression:ab,ti)

=4,101,858

#3. 'cardiovascular disease'/exp OR 'heart infarction'/exp OR 'heart disease'/exp OR

'atherosclerosis'/exp OR 'heart muscle ischemia'/exp OR 'heart muscle revascularization'/exp OR 'acute coronary syndrome'/exp OR 'ischemic heart disease'/exp OR 'angioplasty'/exp OR 'coronary artery bypass graft'/exp OR 'chronic total occlusion'/exp OR 'percutaneous coronary intervention'/exp OR 'percutaneous coronary revascularization'/exp

=4704886

#4. 'cardiovascular disease':ab,ti OR 'myocardial infarction':ab,ti OR 'heart infarction':ab,ti OR 'heart attack':ab,ti OR 'heart disease':ab,ti OR atherosclerosis:ab,ti OR atherosclerotic:ab,ti OR 'myocardial ischemia':ab,ti OR 'myocardial revasculari*':ab,ti OR 'acute coronary syndrome':ab,ti OR 'coronary heart disease':ab,ti OR 'coronary artery disease':ab,ti OR 'coronary disease':ab,ti OR 'ischemic heart disease':ab,ti OR ascvd:ab,ti OR ihd:ab,ti OR ami:ab,ti OR acs:ab,ti OR chd:ab,ti OR cad:ab,ti OR angioplasty:ab,ti OR 'coronary artery bypass':ab,ti OR 'chronic total occlusion':ab,ti OR 'chronic total coronary occlusion':ab,ti OR cto:ab,ti OR 'percutaneous coronary intervention':ab,ti OR 'percutaneous coronary revasculari*':ab,ti OR 'percutaneous coronary angioplasty':ab,ti OR pci:ab,ti OR 'major adverse cardiovascular events':ab,ti OR 'major adverse cardiac events':ab,ti OR mace:ab,ti

=1111566

#5. #3 OR #4

=4,841,054

#6. #1 AND #2 AND #5

=29,072

AND [embase]/lim NOT ([embase]/lim AND [medline]/lim)

=14567

NOT 'conference abstract':it

=3783

NOT (animal* NOT human*)

=3650

Publication date limitation 1990-present

3643 references

Supplemental Note 2. Criteria used for data analyses from the included studies.

The objective of this study was to identify prognostic factors that may refine CVD risk prediction beyond already known risk factors. Therefore, we excluded from the analysis those studies that evaluated biomarkers already established as CVD risk factors (Supplemental Table 4), such as smoking, hypertension, microalbuminuria, BMI, and dyslipidemia, as defined in the 2021 European Society of Cardiology (ESC) guidelines on cardiovascular disease prevention in clinical practice.[1] However, we considered including biomarkers that evaluated a novel variation of an established CVD risk factor, such as HbA1c variability compared to a single time-point measurement.

The strength and quality of evidence in observational prognostic studies depend on how well any known or suspected confounding variables between the prognostic factor and the outcome are accounted for. To be considered a novel risk marker, a biomarker must improve risk prediction beyond traditional markers. Thus, we began by excluding all studies that did not adjust for any CVD risk factors.

Supplemental Table 1. Participant Intervention Comparison Outcomes and Setting (PICOS) framework.

Items	Details
Participants	Patients with Type 2 Diabetes
Intervention/Exposure	Non-Genetic Biomarkers, or Genetic-Biomarkers, or Risk Scores/Engine
Comparison	Not applicable
Outcomes	Coronary heart disease (CHD) and/or cardiovascular (CV) mortality
Study Design	Longitudinal studies

Supplemental Table 2. Risk of Bias Assessment using Modified Newcastle-Ottawa Scale.

**Items**	**Quality Points**	**Risk of bias**
Representativeness of the exposed cohort		
1. Truly representative	3	Low
2. Somewhat representative	2	Medium
3. Selected group	1	High
4. No description of the derivation of the cohort	0	High
Selection of the non-exposed cohort		
1. Drawn from the same community as the exposed cohort	2	Low
2. Drawn from a different source	1	Medium
3. No description of the derivation of the non-exposed cohort	0	High
Ascertainment of exposure		
1. Measured by investigators or from electronic medical record review	2	Low
2. Patient self-reported	1	Medium
3. No description	0	High
Assessment of outcome		
1. Clearly defined outcome (e.g., ICD-10 codes, clinical documentation) or adjudication	3	Low
2. Record linkage (registry)	2	Medium
3. Other (e.g., reported by patients)	1	High
4. No description	0	High
Was follow-up long enough for outcomes to occur?		
1. Yes (3 years for naive CVD event; 1 year for recurrent CVD event after intervention)	3	Low
2. No	1	Medium
Adequacy of follow-up of cohorts		
1. Complete follow-up - all subjects accounted for	3	Low
2. Subjects' loss to follow up unlikely to introduce bias - number lost less than or equal to 20% or description of those lost suggested no different from those followed.	2	Medium
3. Follow up rate less than 80% and no description of those lost	1	Medium
4. No statement	0	High
Number of covariates being included in the models		
1. Highest/fourth quartile of distribution	4	Stratified by tertiles of distribution
2. Third quartile of distribution	3	
3. Second quartile of distribution	2	
4. Lowest/first quartile of distribution	1	
Number of established CVD risk factors being accounted for		
1. N > 75 percentile	6	Stratified by tertiles of distribution
2. 50 < N ≤ 75 percentile	4	
3. 25 < N ≤ 50 percentile	2	
4. N ≤ 25 percentile	0	

Supplemental Table 3. Established CVD risk factors according to 2021 ESC Guidelines[1].

Established CVD Risk Factors
• Age
• Sex/gender
• Hypertension (systolic/diastolic blood pressure, treated hypertension)
• Established CVD (cardiovascular disease, cerebrovascular, PVD, heart failure)
• Antithrombotic treatment (aspirin)
• Dyslipidemia or hyperlipidemia (LDL, HDL, total cholesterol)
• Lipid-lowering treatment (statin, PCSK-9 inhibitor, ezetimibe)
• A1C
• Insulin therapy
• Duration of diabetes
• Atrial fibrillation
• Overweight/obesity/adiposity (body weight, BMI)
• Cigarette smoking
• Nephropathy or chronic kidney disease
• eGFR
• Albumin/creatinine ratio (albuminuria, both micro + macro) or proteinuria
• Retinopathy
• Neuropathy
• Any Microvascular disease
• Geographic region
• Race/ethnicity

CVD, cardiovascular disease; PVD, peripheral vascular disease; LDL, low-density lipoprotein; HDL, high-density lipoprotein; PCSK-9, proprotein convertase subtilisin/kexin-type 9; A1C, hemoglobin A1C; BMI, body mass index; eGFR, estimated glomerular filtration rate.

Supplemental Table 4. Evaluation of the clinical utility of new biomarkers included in the present meta-analysis.

Step	Evaluation	Description
1	Association	Has a statistically significant association been reported with cardiovascular disease for the new biomarker with and without adjustment for traditional risk factors?
2	Discrimination	Does addition of the new biomarker to a model with traditional risk factors lead to significant improvement in discrimination (assessed by the C-statistic)?
3	Net reclassification improvement	Does addition of the new biomarker to a model with traditional risk factors lead to appropriate reclassification of people to either high- or low-risk status?
3	Integrated discrimination index	Quantification of predicted probabilities of cardiovascular events and non-cardiovascular events based on inclusion of the biomarker in a model with traditional risk factors

Modified from[1-3]. Calibration, which is an additional parameter used for evaluation of clinical utility by Hlatky et al., was not used in the present meta-analysis. Calibration is defined as *"Does addition of the new factor to a traditional risk factor model result in improved calibration, defined as agreement between the predicted and observed rates of end points?"*; very few articles provided sufficient information to allow evaluation of this parameter.

Supplemental Figure 1 : Variations in the definitions of cardiovascular outcomes among the included studies

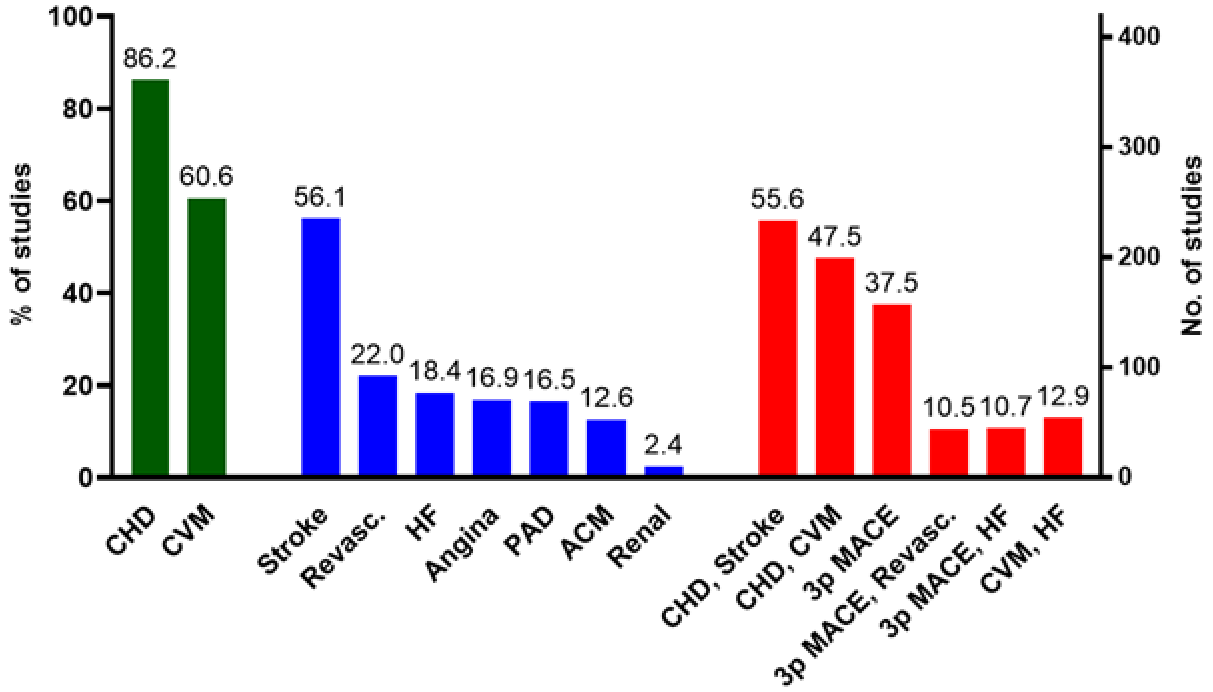

Legend: Variations in the definitions of cardiovascular outcomes among the included studies. Green columns represent the "required" endpoints (Coronary Heart Disease [CHD] and/or cardiovascular mortality [CVM]). Blue columns represent additional endpoints (e.g. Stroke, Revasc. [Revascularization], Heart Failure [HF], peripheral artery disease [PAD] or all-cause mortality [ACM]) being evaluated in the studies. Red columns represent the combination of specific outcomes in groups of general interest (e.g. 3-point Major Adverse Cardiovascular Events [MACE]). Note: End-points (and groups of end-points) are not mutually exclusive; therefore, each study can be counted multiple times in this graph (e.g. one study can have both CHD and CVM; or a study evaluating a composite outcome of CHD, CVM, Stroke and Revascularization can be included both in "3p MACE" and "3p MACE, Revasc." columns).

Supplemental Figure 2: Meta-analysis of N-terminal pro-B-type natriuretic peptide (NTpro-BNP)

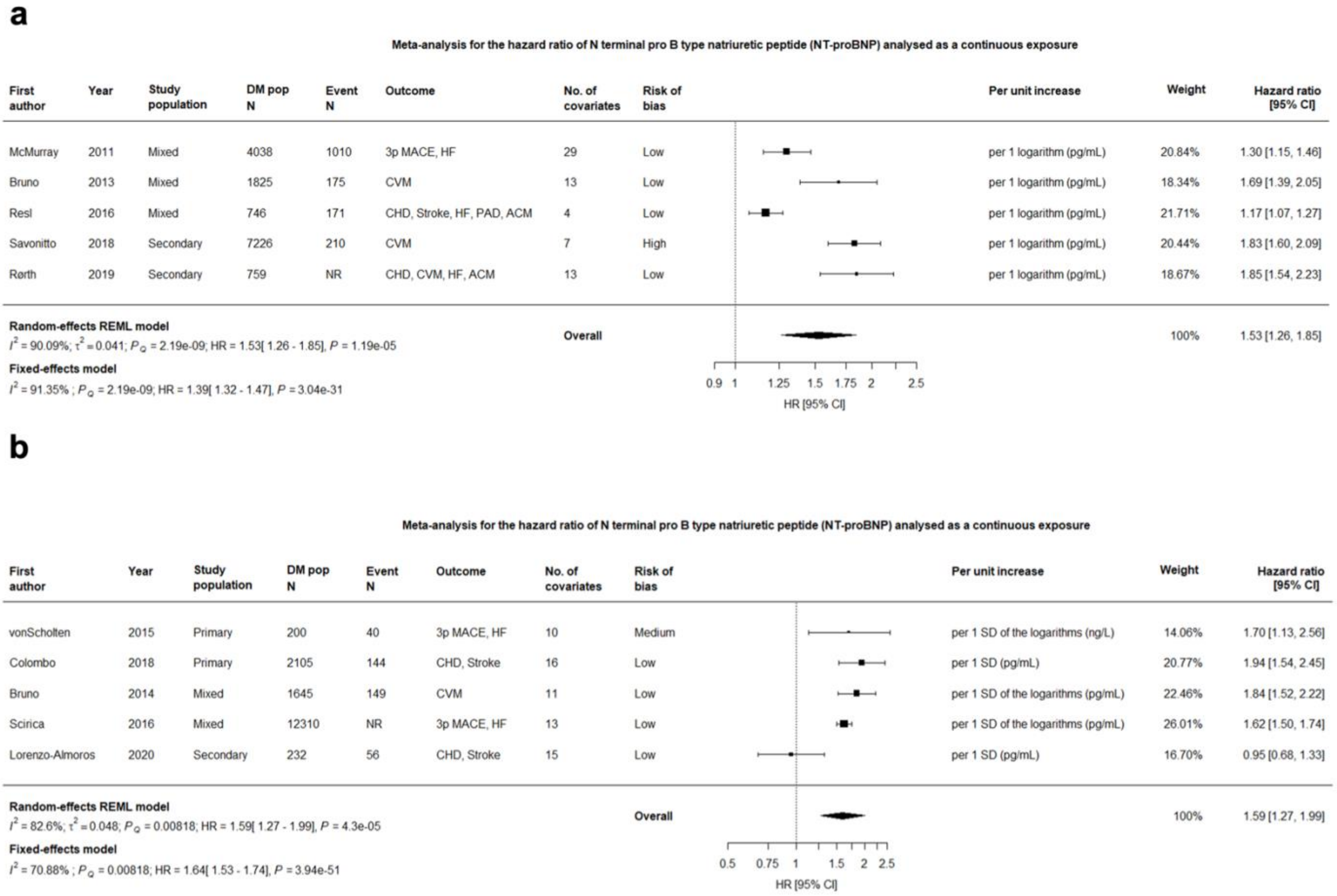

Legend: studies analyzed as a continuous exposure with a logarithm transformation (**Panel a**) and as a continuous exposure with a z-score transformation (**Panel b**). HR, hazard ratio; CI, confidence interval; DM pop N, sample size for diabetes population; Event N, number of individuals developed who CVD outcomes; 3p MACE, 3-point major adverse cardiovascular events; HF, heart failure; CHD, coronary heart disease; PAD, peripheral artery disease; CVM, cardiovascular mortality; ACM, all-cause mortality. NR, not reported.

Supplemental Figure 3: Meta-analysis of Troponin T (TnT) studies

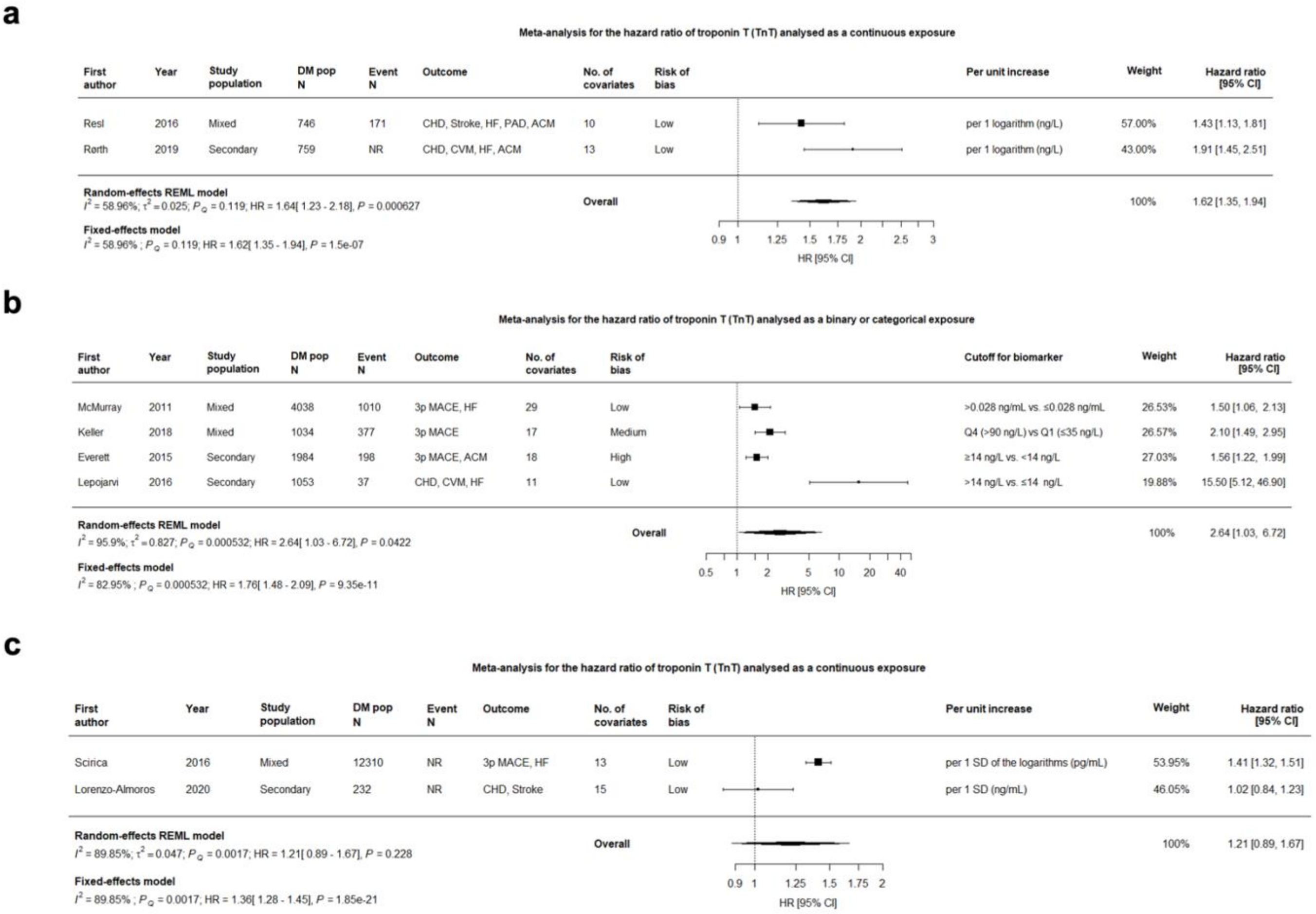

Legend: Meta-analysis of Troponin T (TnT) studies presented in three formats: logarithmic transformation of continuous exposure (**Panel a**), binary or categorical exposure (**Panel b**), and z-score transformation of continuous exposure (**Panel c**). The analysis includes hazard ratio (HR), confidence interval (CI), sample size for diabetes population (DM pop N), number of individuals who developed cardiovascular disease (CVD) outcomes (Event N), heart failure (HF), coronary heart disease (CHD), cardiovascular mortality (CVM), peripheral arterial disease (PAD), all-cause mortality (ACM), and instances where data was not reported (NR).

Supplemental Figure 4: Forest plots of Coronary artery calcium score (CACS), carotid plaque, and c-reactive protein (CRP).

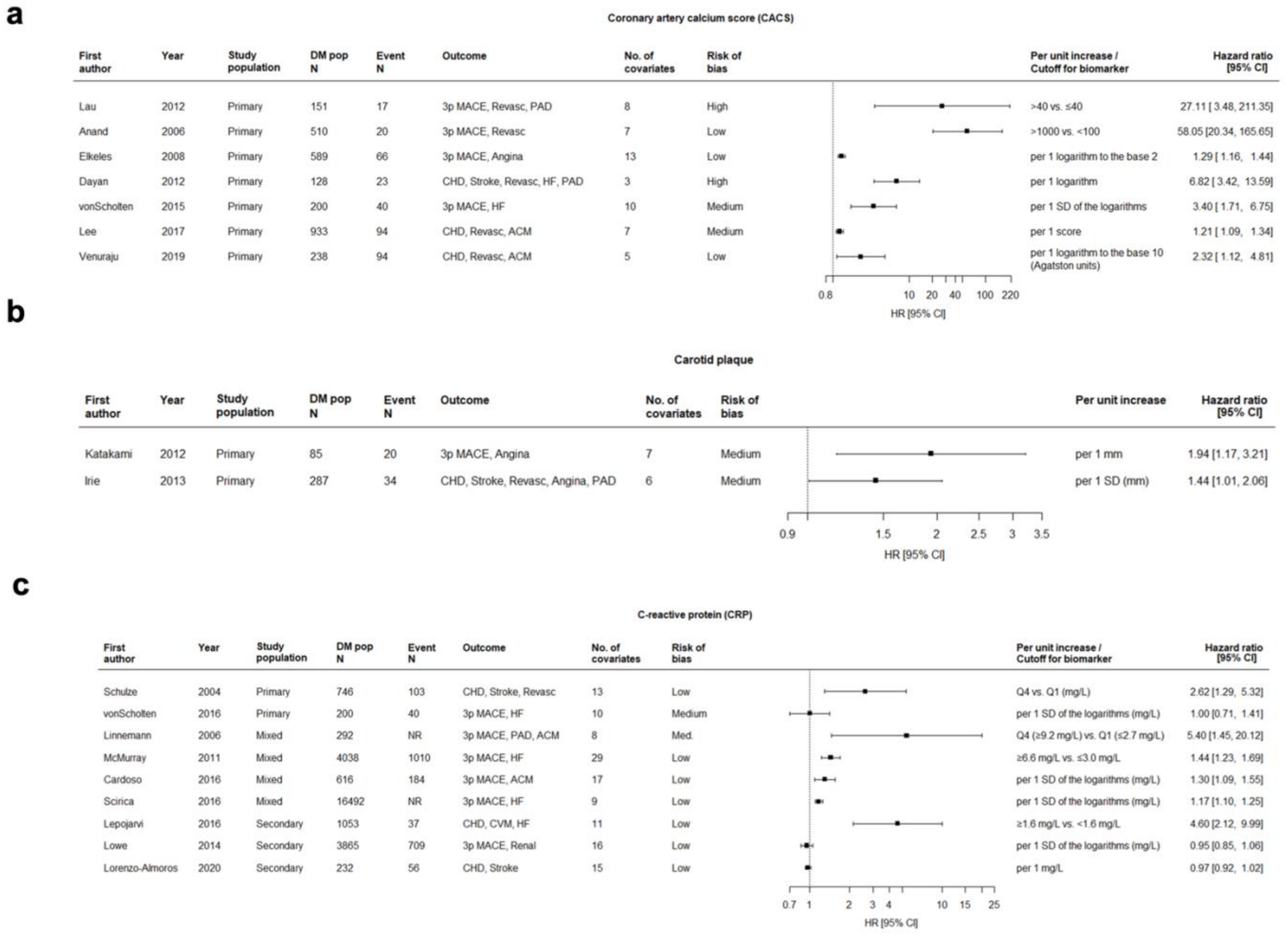

Legend: Forest plots of Coronary artery calcium score (CACS) as binary and continuous exposure (**Panel a**), carotid plaque as a continuous exposure (**Panel b**), and c-reactive protein (CRP) as binary and continuous exposure (**Panel c**). The analysis includes hazard ratio (HR), confidence interval (CI), sample size for diabetes population (DM pop N), number of individuals who developed cardiovascular disease (CVD) outcomes (Event N), 3-point major adverse cardiovascular events (3p MACE), revascularization (Revasc), heart failure (HF), coronary heart disease (CHD), peripheral artery disease (PAD), cardiovascular mortality (CVM), all-cause mortality (ACM), and standard deviation (SD).

Supplemental Figure 5: Forest plots of galectin-3, growth differentiation factor (GDF-15), pulse wave velocity (PWV), and single-photon emission computed tomography (SPECT) scintigraphy.

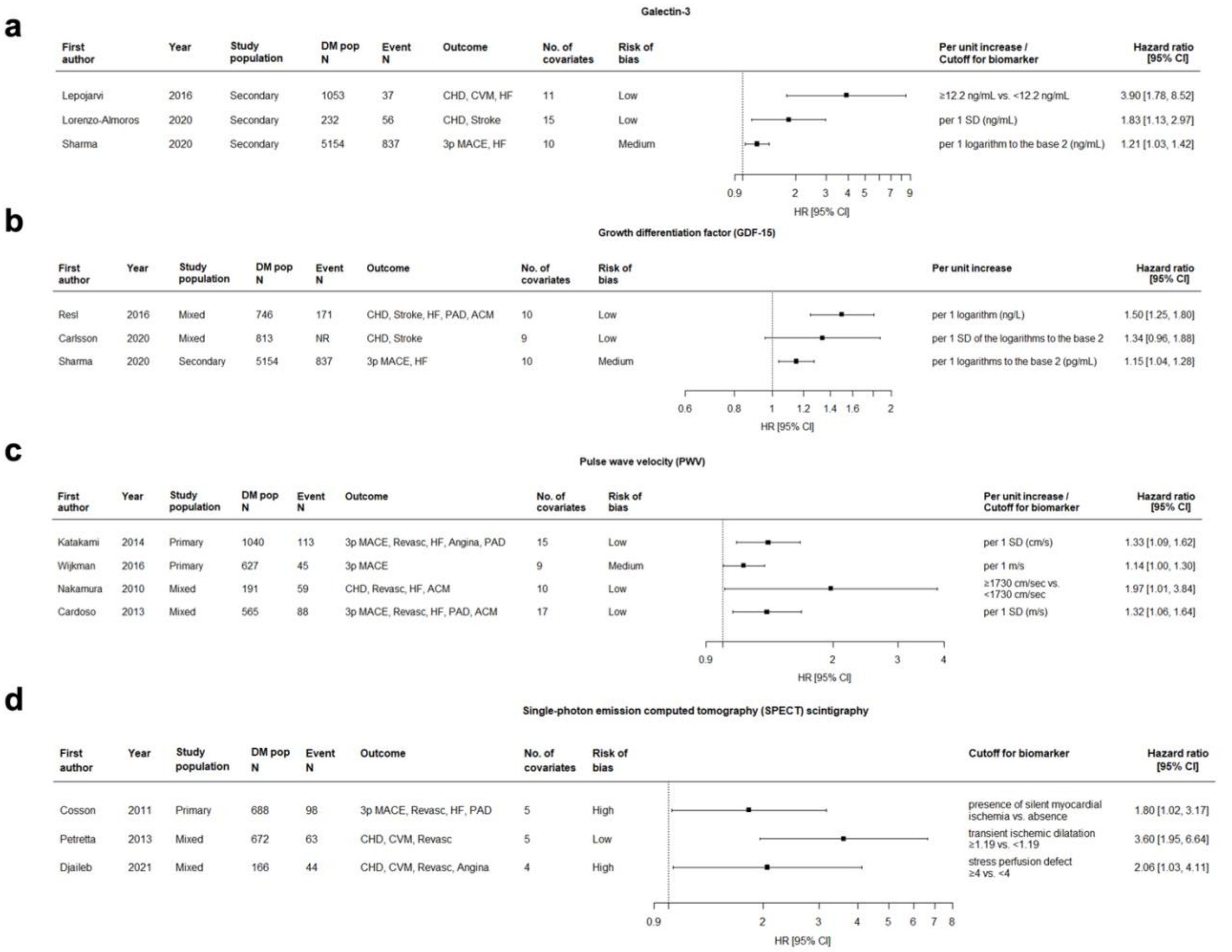

Legend: Forest plots of galectin-3 as binary and continuous exposure (**Panel a**), growth differentiation factor (GDF-15) as continuous exposure (**Panel b**), pulse wave velocity (PWV) as binary and continuous exposure (**Panel c**), and single-photon emission computed tomography (SPECT) scintigraphy as binary exposure (**Panel d**). The analysis includes hazard ratio (HR), confidence interval (CI), sample size for diabetes population (DM pop N), number of individuals who developed cardiovascular disease (CVD) outcomes (Event N), 3-point major adverse cardiovascular events (3p MACE), revascularization (Revasc), heart failure (HF), coronary heart disease (CHD), peripheral artery disease (PAD), cardiovascular mortality (CVM), all-cause mortality (ACM), and standard deviation (SD). Instances where data was not reported (NR) are also noted.

Supplemental Figure 6: Forest plots of troponin I (TnI), and triglyceride glucose (TyG) index.

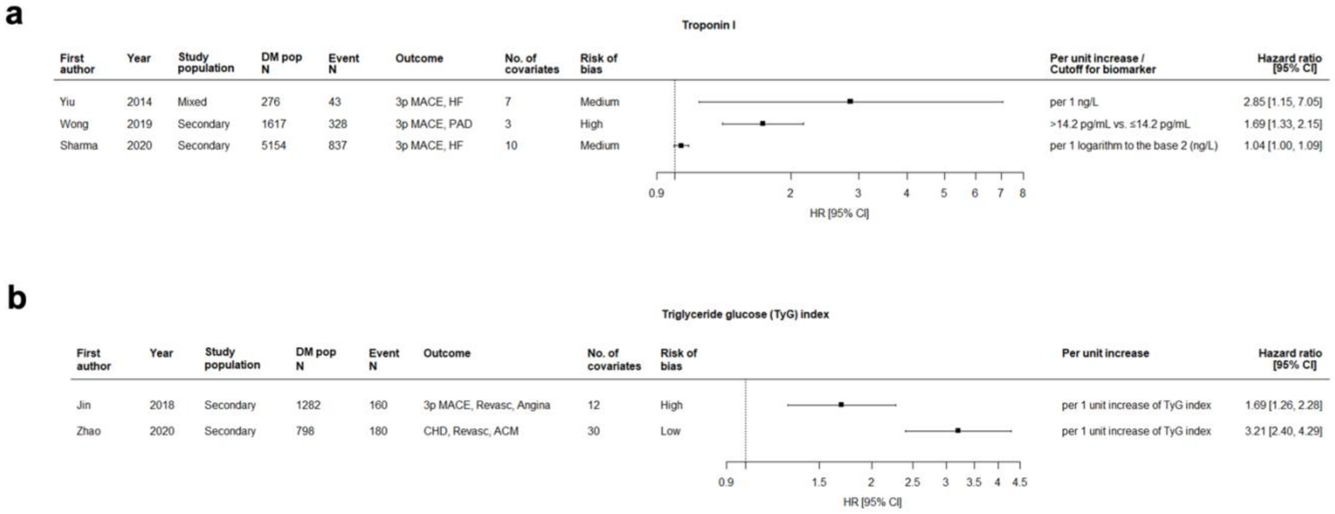

Legend: Forest plots of troponin I (TnI) as both continuous and binary exposure (**Panel a**), and triglyceride glucose (TyG) index as a continuous exposure (**Panel b**). The analysis includes hazard ratio (HR), confidence interval (CI), sample size for diabetes population (DM pop N), number of individuals who developed cardiovascular disease (CVD) outcomes (Event N), 3-point major adverse cardiovascular events (3p MACE), heart failure (HF), peripheral artery disease (PAD), revascularization (Revasc), coronary heart disease (CHD), and all-cause mortality (ACM).

Supplemental Figure 7: Meta-analysis of studies on C-reactive protein (CRP), pulse wave velocity (PWV), and triglyceride glucose (TyG).

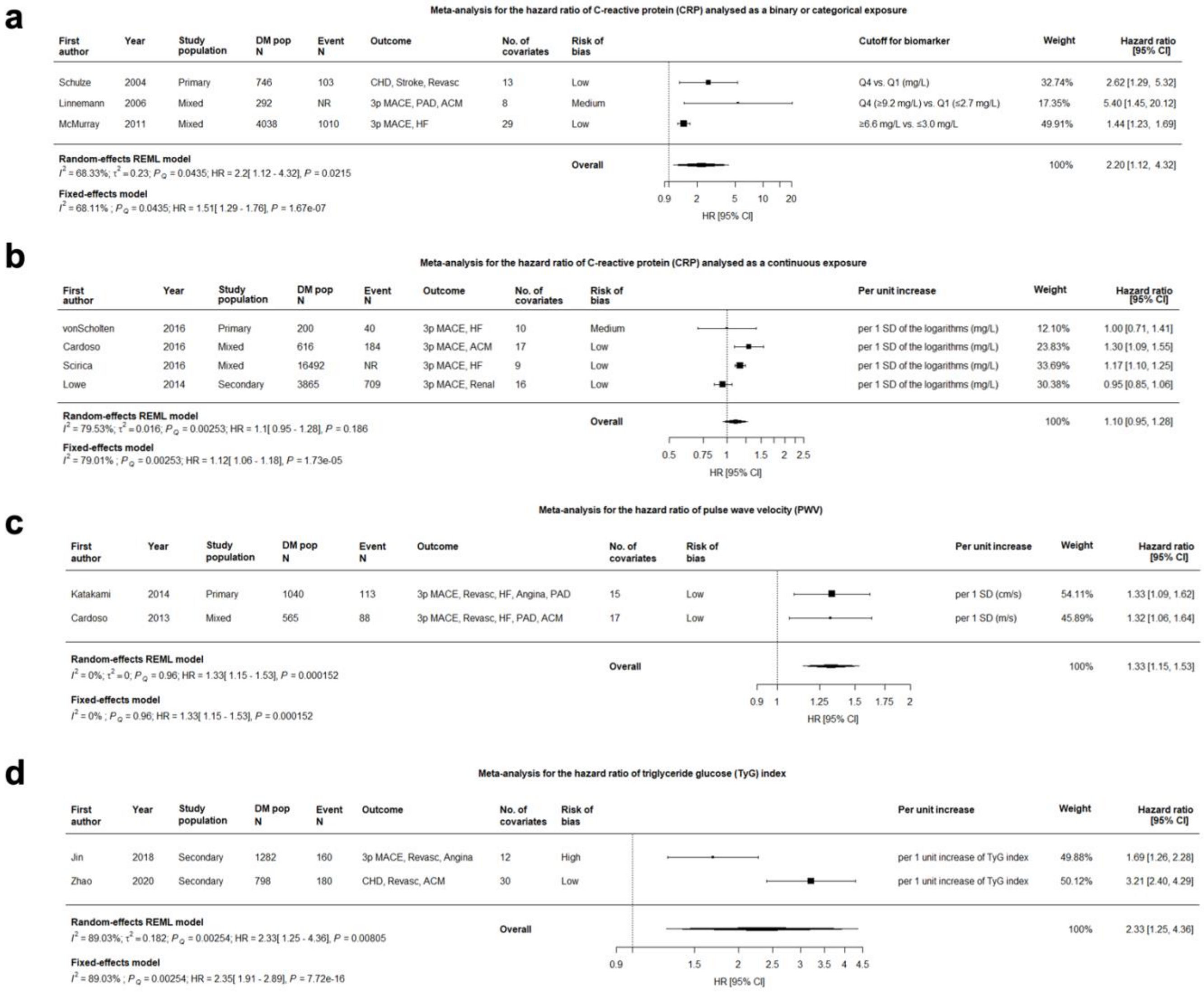

Legend: Meta-analysis of studies on C-reactive protein (CRP) analyzed as both binary or categorical exposure (**Panel a**) and as a continuous exposure with a z-score of logarithm transformation (**Panel b**), along with pulse wave velocity (PWV) analyzed as a continuous exposure with a z-score transformation (**Panel c**), and triglyceride glucose (TyG) index analyzed as a continuous exposure (**Panel d**). For the overall pooled estimate, a random-effects model was applied only if the heterogeneity test was statistically significant (Cochran's Q test p-value <0.1 or the I^2 statistic > 75%). The analysis includes hazard ratio (HR), confidence interval (CI), sample size for diabetes population (DM pop N), number of individuals who developed cardiovascular disease (CVD) outcomes (Event N), 3-point major adverse cardiovascular events (3p MACE), revascularization (Revasc), heart failure (HF), coronary heart disease (CHD), peripheral artery disease (PAD), all-cause mortality (ACM), quartile (Q), standard deviation (SD), and instances where data was not reported (NR).

Supplemental Figure 8: Meta-analysis of studies on Genetic Risk Scores (GRS) for Coronary Heart Disease and *GLUL* (rs10911021).

a

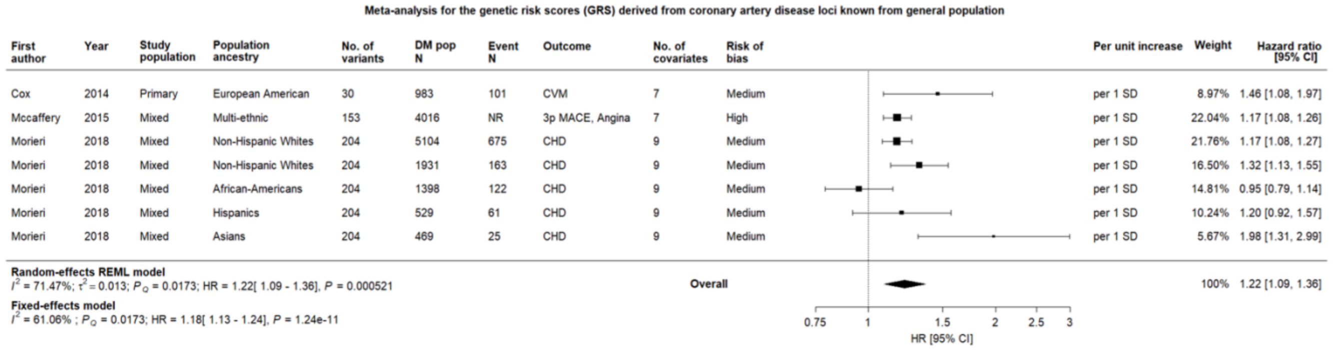

b

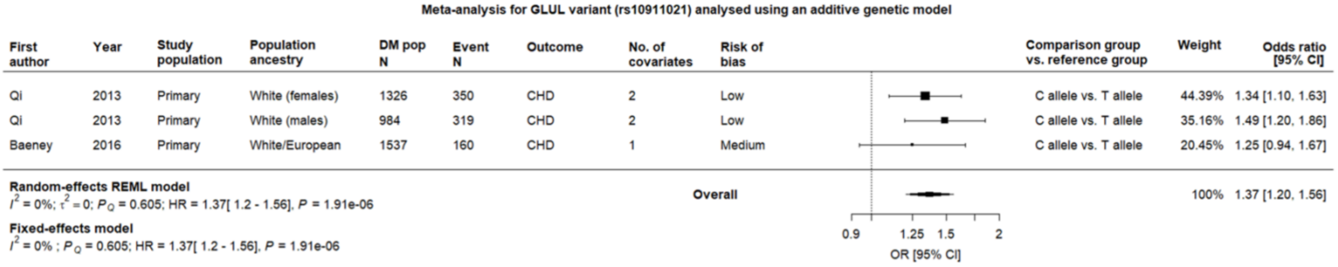

Legend: Meta-analysis of studies on Genetic Risk Scores (GRS) for Coronary Heart Disease analyzed as a continuous exposure with a z-score transformation (**Panel a**) and for the *GLUL* variant rs10911021 (**Panel b**). For the overall pooled estimate, a random-effects model was applied only if the heterogeneity test was statistically significant (Cochran's Q test p-value <0.1 or the I^2 statistic > 75%). The analysis includes hazard ratio (HR), odds ratio (OR), confidence interval (CI), sample size for diabetes population (DM pop N), number of individuals who developed cardiovascular disease (CVD) outcomes (Event N), 3-point major adverse cardiovascular events (3p MACE), cardiovascular mortality (CVM), coronary heart disease (CHD), and standard deviation (SD).

Supplemental Figure 9: Summary of c-statistics of risk scores for predicting cardiovascular outcomes in internal validation cohorts.

C-statistics of risk scores (internal validation cohorts)

First author	Year	Risk score	Derivation country(ies)	Risk of bias	C-statistic [95% CI]
Woodward	2016	AD-ON	United States	High	0.66 [0.65, 0.68]
Kengne	2011	ADVANCE	Europe/Asia	High	0.70 [0.68, 0.73]
Woodward	2016	ADVANCE	Europe/Asia	High	0.66 [0.64, 0.67]
Piniés	2014	BASCORE	Spain	Low	0.63 [0.57, 0.69]
Shao	2018	BRAVO	Shao	Low	0.69 [0.67, 0.71]
Cox	2014	Cambridgeshire	United Kingdom	Medium	0.71 [0.70, 0.72]
Lagani	2015	CVD-DCCT	United States	High	0.68 [0.49, 0.87]
Lagani	2015	CVD-EDIC	United States	High	0.66 [0.51, 0.81]
Davis	2020	FDS-2	Australia	High	0.82 [0.79, 0.85]
Quan	2019	HKU-SG	China/Singapore	High	0.70 [0.70, 0.70]
Wan	2018	Hong Kong Hospital Authority	China	Low	0.72 [0.71, 0.73]
Wan	2018	Hong Kong Hospital Authority	China/Singapore	Low	0.70 [0.69, 0.72]
Tanaka	2013	JJ Risk Engine	Japan	Medium	0.72 [0.66, 0.79]
Ramírez-Prado	2015	Ramirez-Prado 2015	Spain	Medium	0.73 [0.66, 0.80]
Basu	2017	RECODe	United States	High	0.69 [0.67, 0.71]
Venäläinen	2020	SPRINT	United States	Medium	0.70 [0.66, 0.74]
Piniés	2014	UKPDS-RE	Spain	Low	0.62 [0.57, 0.67]
Yu	2019	Zhengzhou University	China	Low	0.74 [0.73, 0.75]

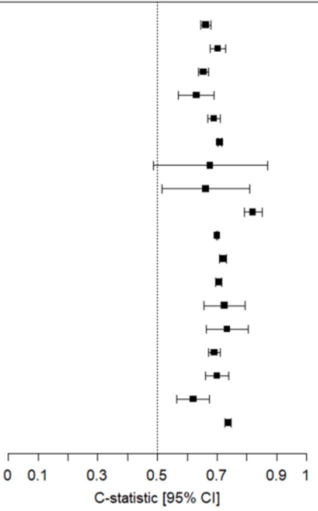

Legend: CI, confidence interval.

Supplemental Figure 10: Summary of c-statistics of risk scores for predicting cardiovascular outcomes in external validation cohorts.

C-statistics of risk scores (external validation cohorts)

First author	Year	Risk score	Year original risk score published	Derivation country(ies)	Validation cohort	Country(ies) of validation cohort	Risk of bias	C-statistic [95% CI]
Basu	2017	ACC/AHA PCEs	2013	United States	LOOK AHEAD	United States	High	0.66 [0.64, 0.68]
Kengne	2011	ADVANCE	2011	Europe/Asia	DIABHYCAR	Multinational	High	0.68 [0.65, 0.72]
vanderLeeuw	2015	ADVANCE	2011	Europe/Asia	EPIC-NL	Netherlands	Medium	0.62 [0.54, 0.70]
vanderLeeuw	2015	ADVANCE	2011	Europe/Asia	EPIC-Potsdam	Germany	Medium	0.67 [0.59, 0.75]
vanderLeeuw	2015	ADVANCE	2011	Europe/Asia	SMART	Netherlands	Medium	0.68 [0.59, 0.77]
Wan	2018	ADVANCE	2011	Europe/Asia	Hong Kong Hospital Authority (men)	China	Low	0.69 [0.68, 0.70]
Wan	2018	ADVANCE	2011	Europe/Asia	Hong Kong Hospital Authority (women)	China	Low	0.71 [0.70, 0.72]
Read	2018	ADVANCE	2011	Europe/Asia	National Scottish Diabetes Register	Scotland	Medium	0.67 [0.66, 0.67]
Cox	2014	Cambridgeshire	2018	United Kingdom	RCT of Peer Support in Type 2 Diabetes trial	United Kingdom	Medium	0.71 [0.70, 0.72]
vanderLeeuw	2015	CHS	2013	United States	EPIC-NL	Netherlands	Medium	0.54 [0.46, 0.62]
vanderLeeuw	2015	CHS	2013	United States	EPIC-Potsdam	Germany	Medium	0.61 [0.52, 0.70]
vanderLeeuw	2015	CHS	2013	United States	SMART	Netherlands	Medium	0.68 [0.59, 0.77]
vanderLeeuw	2015	CHS	2013	United States	National Scottish Diabetes Register	Scotland	Medium	0.67 [0.67, 0.68]
Lagani	2015	CVD-DCCT	2015	United States	Chorleywood Medical Center	United Kingdom	High	0.71 [0.62, 0.81]
Lagani	2015	CVD-EDIC	2015	United States	Chorleywood Medical Center	United Kingdom	High	0.66 [0.51, 0.81]
vanderLeeuw	2015	CVD-EDIC	2015	United States	EPIC-NL	Netherlands	Medium	0.58 [0.50, 0.66]
vanderLeeuw	2015	CVD-EDIC	2015	United States	EPIC-Potsdam	Germany	Medium	0.68 [0.60, 0.76]
vanderLeeuw	2015	CVD-EDIC	2015	United States	SMART	Netherlands	Medium	0.69 [0.59, 0.79]
Read	2018	CVD-EDIC	2015	United States	National Scottish Diabetes Register	Scotland	Medium	0.67 [0.67, 0.67]
Davis	2020	FDS-2	2020	Australia	Busselton Health Study	Australia	High	0.81 [0.74, 0.88]
vanderHeijden	2009	FRS	1998	United States	Hoorn Study	Netherlands	Low	0.63 [0.50, 0.76]
Piniés	2014	FRS-RS	2014	Spain	BASCORE	Spain	Low	0.58 [0.51, 0.65]
Quan	2019	JADE	2009	Asia/Pacific	Hong Kong Dataset	China	High	0.68 [0.68, 0.69]
vanderLeeuw	2015	NDR	2008	Sweden	EPIC-NL	Netherlands	Medium	0.64 [0.56, 0.72]
vanderLeeuw	2015	NDR	2008	Sweden	EPIC-Potsdam	Germany	Medium	0.67 [0.60, 0.74]
vanderLeeuw	2015	NDR	2008	Sweden	SMART	Netherlands	Medium	0.64 [0.54, 0.74]
Wan	2018	NDR	2008	Sweden	Hong Kong Hospital Authority (men)	China	Low	0.69 [0.68, 0.70]
Wan	2018	NDR	2008	Sweden	Hong Kong Hospital Authority (women)	China	Low	0.71 [0.70, 0.72]
Read	2018	NDR	2008	Sweden	National Scottish Diabetes Register	Scotland	Medium	0.66 [0.66, 0.67]
Elley	2010	NZ DCS	2010	New Zealand	New Zealand (South)	New Zealand	Medium	0.67 [0.66, 0.68]
vanderLeeuw	2015	NZ DCS	2010	New Zealand	EPIC-NL	Netherlands	Medium	0.63 [0.55, 0.71]
vanderLeeuw	2015	NZ DCS	2010	New Zealand	EPIC-Potsdam	Germany	Medium	0.66 [0.59, 0.73]
vanderLeeuw	2015	NZ DCS	2010	New Zealand	SMART	Netherlands	Medium	0.67 [0.57, 0.77]
Wan	2018	NZ DCS	2010	New Zealand	Hong Kong Hospital Authority (men)	China	Low	0.69 [0.68, 0.70]
Wan	2018	NZ DCS	2010	New Zealand	Hong Kong Hospital Authority (women)	China	Low	0.71 [0.70, 0.72]
Read	2018	QRISK2	2008	England/Wales	National Scottish Diabetes Register	Scotland	Medium	0.68 [0.67, 0.68]
Basu	2017	RECODe	2017	United States	LOOK AHEAD	United States	High	0.73 [0.71, 0.75]
vanderHeijden	2009	SCORE	2003	Europe	Hoorn Study	Netherlands	Low	0.66 [0.54, 0.78]
Venäläinen	2020	SPRINT	2017	United States	ACCORD	United States	Medium	0.69 [0.67, 0.71]
Simmons	2009	UKPDS	1998	United Kingdom	Epic Norfolk	United Kingdom	Medium	0.72 [0.66, 0.78]
Davis	2009	UKPDS	1998	United Kingdom	FDS	Australia	Medium	0.68 [0.60, 0.76]
Kengne	2010	UKPDS	1998	United Kingdom	ADVANCE	Europe/Asia	Low	0.68 [0.65, 0.72]
vanDieren	2011	UKPDS	1998	United Kingdom	Epic-NL; Epic-Potsdam	Netherlands/Germany	Low	0.66 [0.51, 0.81]
Tanaka	2013	UKPDS	1998	United Kingdom	JDCS/J-EDIT	Japan	Medium	0.69 [0.63, 0.76]
Piniés	2014	UKPDS	1998	United Kingdom	BASCORE	Spain	Low	0.62 [0.57, 0.67]
Basu	2017	UKPDS	1998	United Kingdom	LOOK AHEAD	United States	High	0.67 [0.65, 0.69]
Quan	2019	UKPDS	1998	United Kingdom	Hong Kong Dataset	China	High	0.68 [0.67, 0.68]
Quan	2019	UKPDS	1998	United Kingdom	Hong Kong Dataset	China	High	0.65 [0.65, 0.66]
Venäläinen	2020	UKPDS	1998	United Kingdom	Hoorn Study	Netherlands	Medium	0.66 [0.55, 0.77]
Yu	2019	Zhengzhou University	2019	China	Zhengzhou University	China	Low	0.75 [0.74, 0.76]

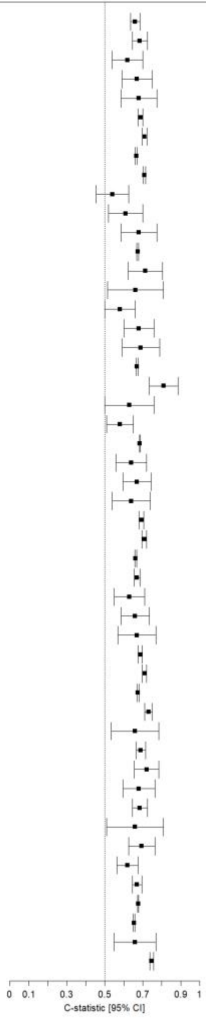

Legend: CI, confidence interval.

Supplemental Figure 11: Concordance of c-statistics between the countries of origin (development cohort) and external validation cohort in non-genetic risk scores.

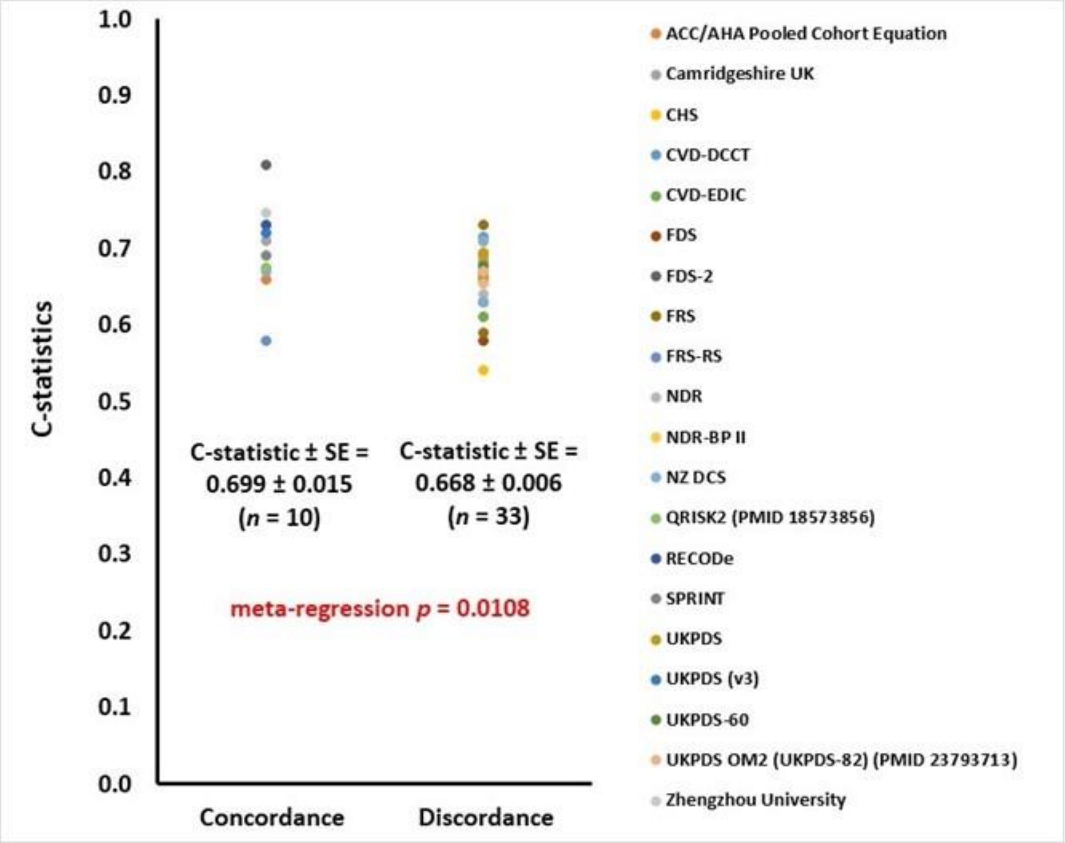

Legend: Concordance of c-statistics between the countries of origin (development cohort) and external validation cohort in non-genetic risk scores. The pooled c-statistics and the corresponding standard error (SE) were computed using a random-effects model. The p-value was obtained from the meta-regression analysis using a mixed-effects model; n, number of studies.

Supplemental Figure 12: Meta-analysis of the ADVANCE risk score, CHS risk score, and CVD-EDIC risk score on external validation.

a

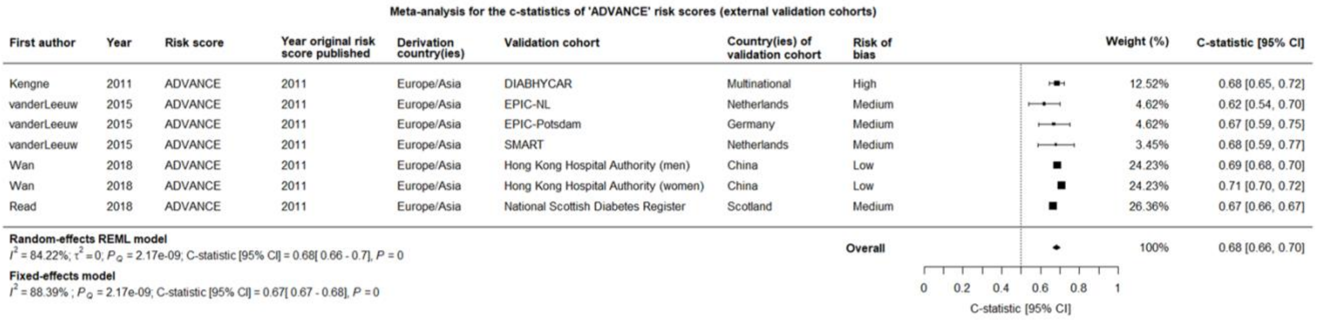

b

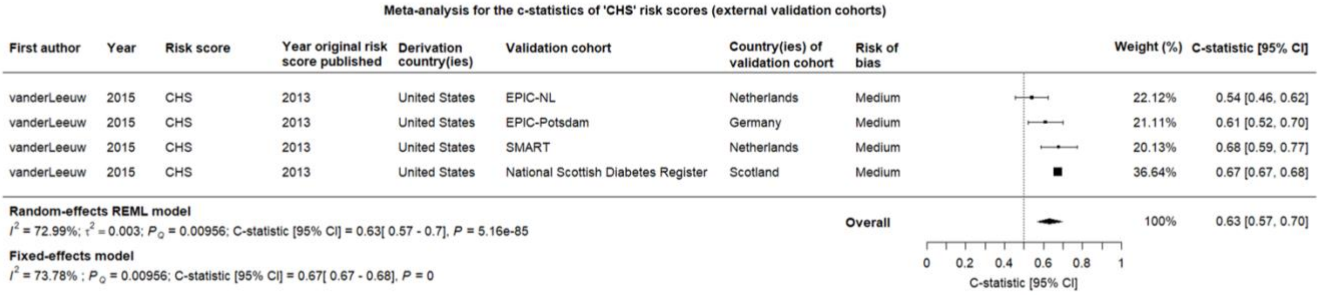

c

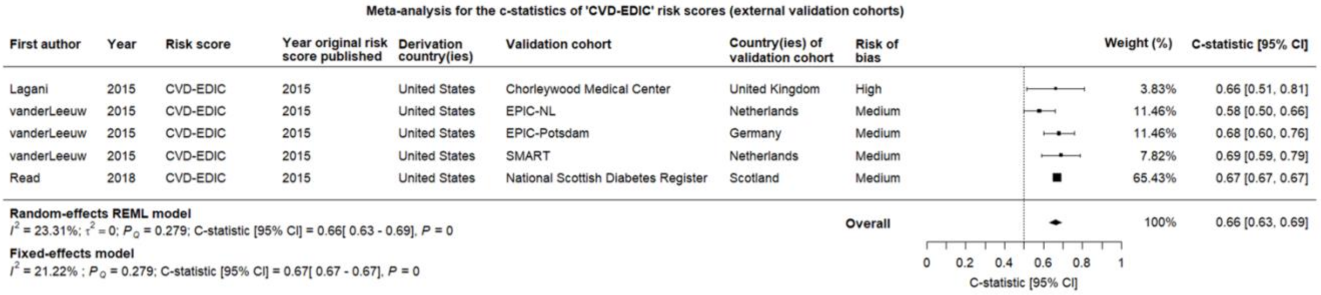

Legend: Meta-analysis of the ADVANCE risk score (**Panel a**), CHS risk score (**Panel b**), and CVD-EDIC risk score (**Panel c**) on external validation. For the overall pooled estimate, a random-effects model was applied only if the heterogeneity test was statistically significant, as indicated by a Cochran's Q test p-value <0.1 or an I^2 statistic > 75%. The analysis includes confidence intervals (CI).

Supplemental Figure 13: Meta-analysis of the NDR risk score, NZ DCS risk score, and UKPDS risk score (Panel c) on external validation

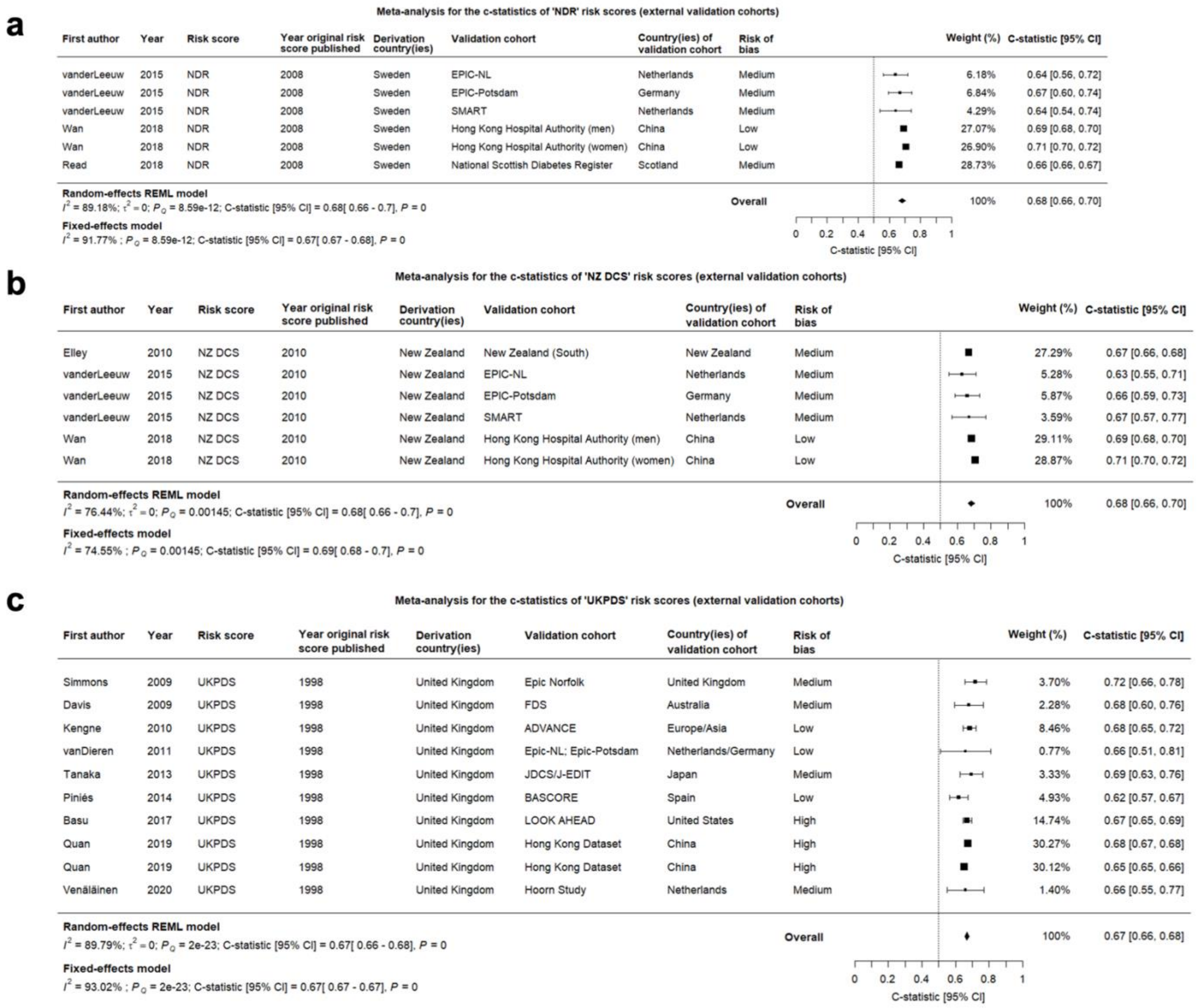

Legend: Meta-analysis of the NDR risk score (**Panel a**), NZ DCS risk score (**Panel b**), and UKPDS risk score (**Panel c**) on external validation. A random-effects model was employed for the overall pooled estimate only when the heterogeneity test was statistically significant, indicated by a Cochran's Q test p-value <0.1 or an I^2 statistic > 75%.

Supplemental Figure 14: Histograms illustrating the distribution of studies based on the number of total adjusted covariates (Panel a) and the distribution based on the number of adjusted traditional cardiovascular disease risk factors (Panel b).

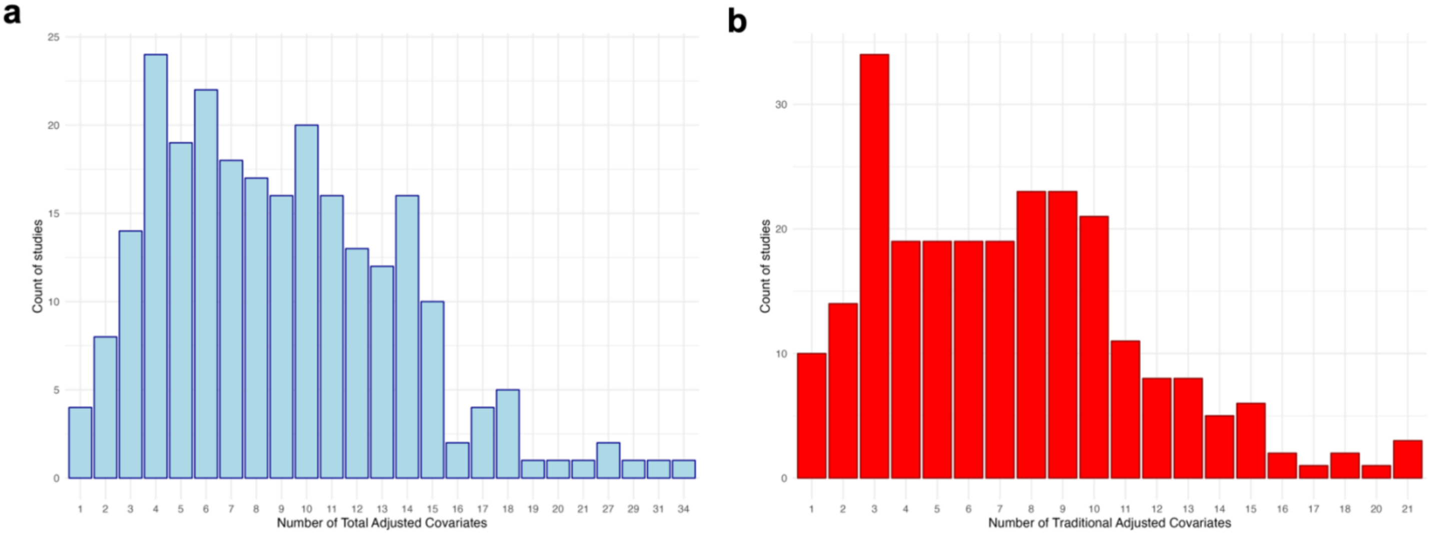

Supplemental Figure 15: The network figure represents the connections of adjusted covariates in 416 studies.

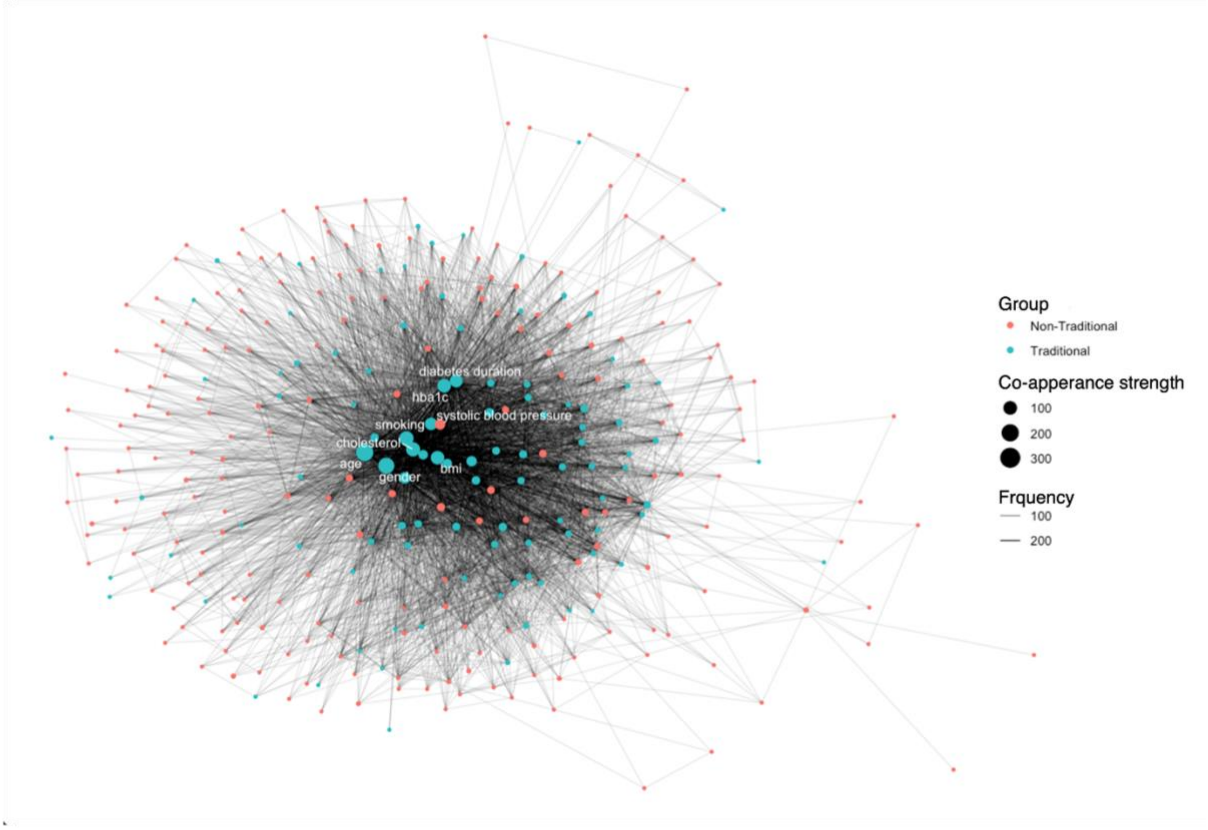

Legend: The nodes in the figure correspond to covariates, and the size of the node represents the frequency of the covariate appearing in the groups of studies. The more centrally located a node, the more important its role as a factor in the network. Nodes are color-coded based on whether they are traditional or non-traditional covariates. The edges (lines connecting co-variates) vary in clarity, indicating the frequency of connections between covariates. An interactive version of this figure can be found here: https://hugofitipaldi.shinyapps.io/T2D_prognostic/

Supplemental Figure 16: Sensitivity analysis of N-terminal pro-B-type natriuretic peptide (NTpro-BNP) and Troponin T (TnT), excluding studies with a high risk of bias.

a

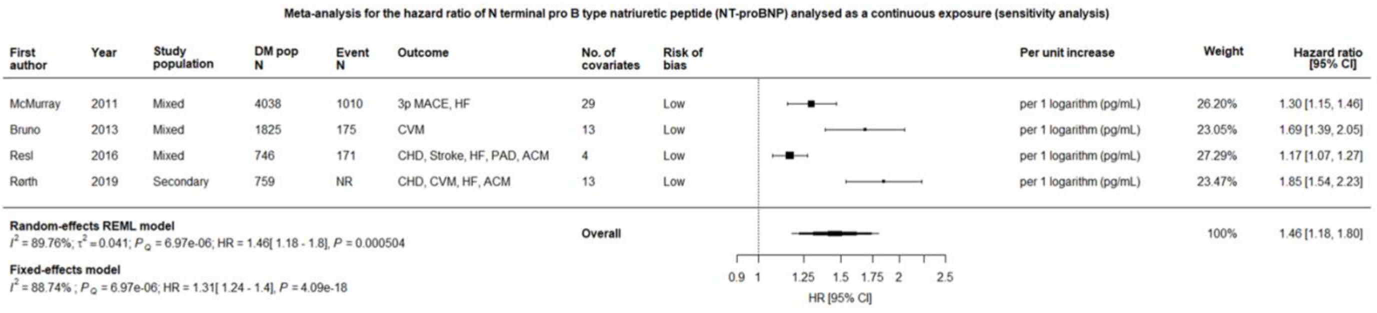

b

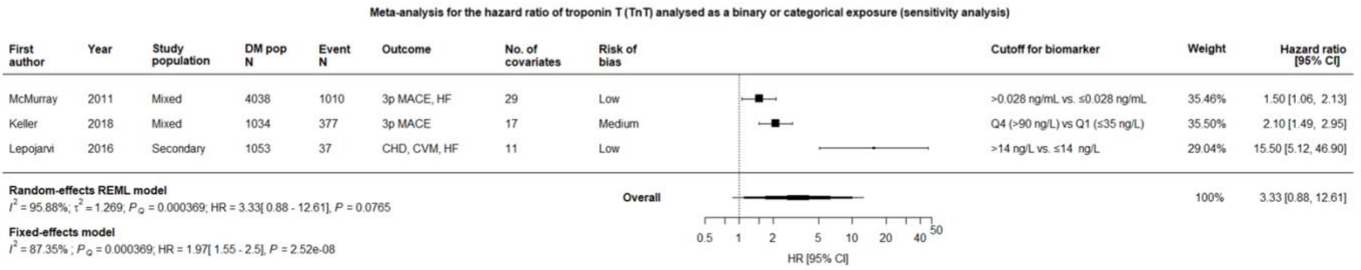

Legend: Sensitivity analysis of N-terminal pro-B-type natriuretic peptide (NTpro-BNP) as a continuous measure (**Panel a**) and Troponin T (TnT) as a binary/categorical measure (**Panel b**), excluding studies with a high risk of bias. For the overall pooled estimate, a random-effects model was employed only if the heterogeneity test was statistically significant (Cochran's Q test p-value <0.1 or the I^2 statistic > 75%). The analysis includes hazard ratio (HR), confidence interval (CI), sample size for diabetes population (DM pop N), number of individuals who developed cardiovascular disease (CVD) outcomes (Event N), 3-point major adverse cardiovascular event (3p MACE), heart failure (HF), coronary heart disease (CHD), cardiovascular mortality (CVM), peripheral arterial disease (PAD), all-cause mortality (ACM), quartile (Q), and instances where data was not reported (NR).

Supplemental Figure 17: Sensitivity Analysis Excluding High Risk of Bias Studies in Genetic Risk Score (GRS) studies.

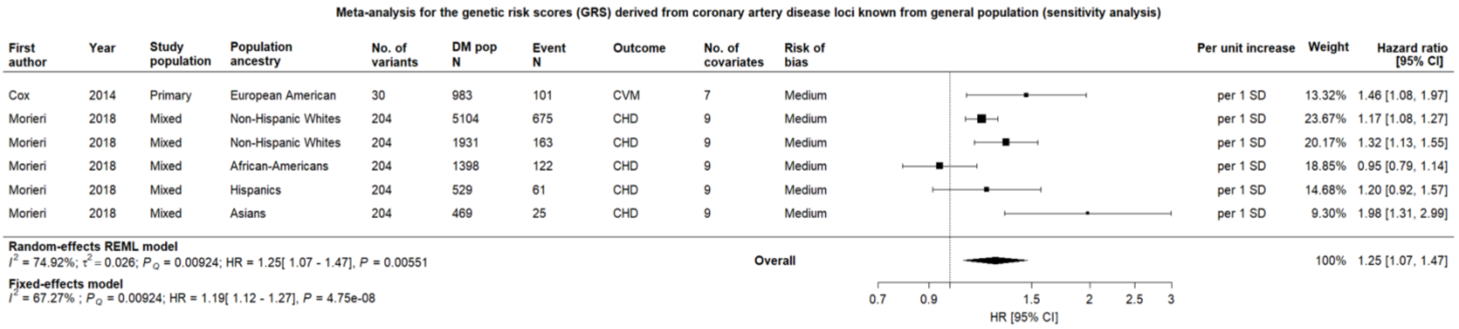

Legend: For the overall pooled estimate, a random-effects model was used only if the heterogeneity test was statistically significant (Cochran's Q test p-value <0.1 or the I^2 statistic > 75%). HR, hazard ratio; CI, confidence interval; DM pop N, sample size for diabetes population; Event N, number of individuals who developed CVD outcomes; CVM, cardiovascular mortality; CHD, coronary heart disease; SD, standard deviation.

Supplemental Figure 18: Sensitivity Analysis Excluding High Risk of Bias Studies for ADVANCE Risk Score, CVD-EDIC Risk Score, and UKPDS Risk Score on external validation.

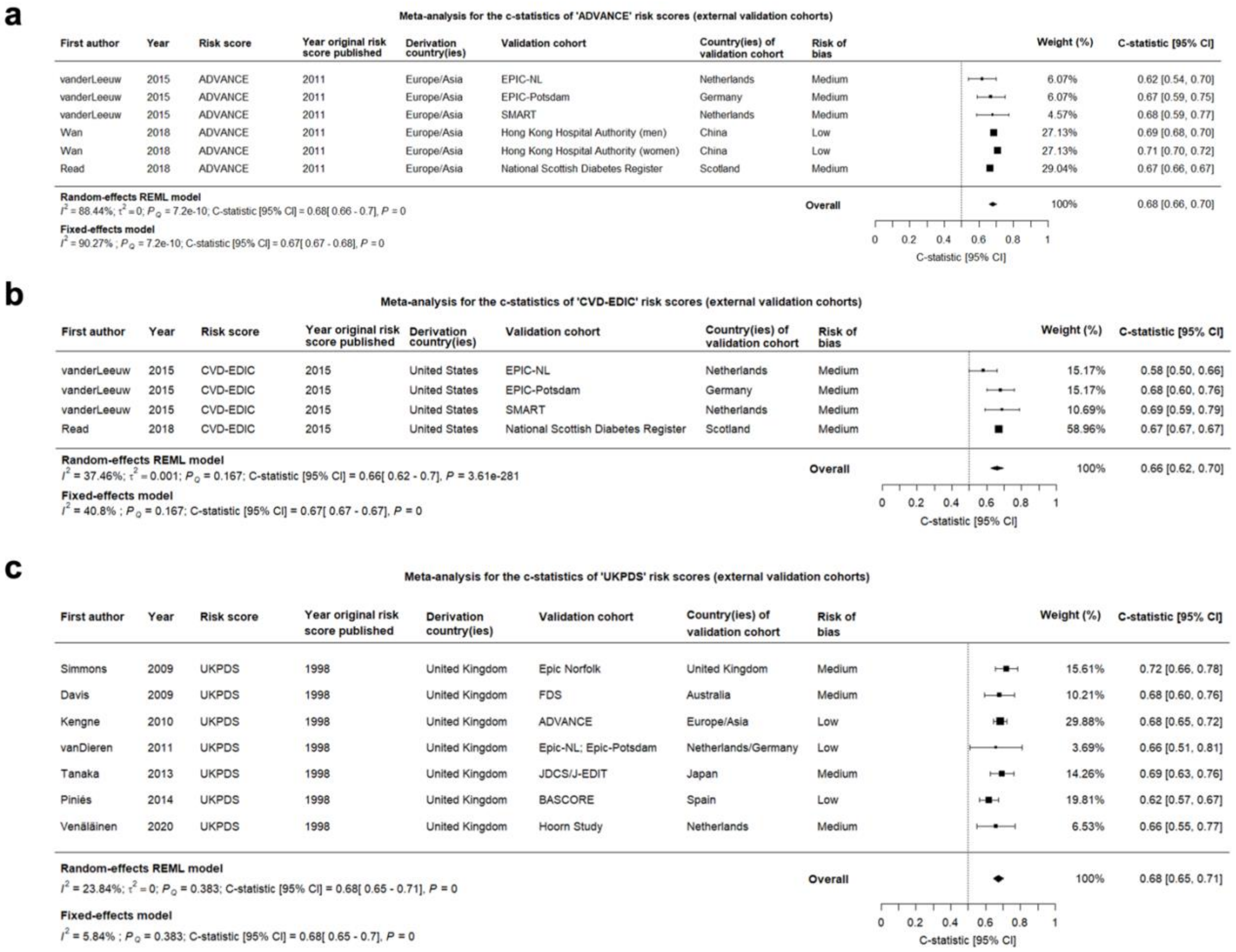

a

Meta-analysis for the c-statistics of 'ADVANCE' risk scores (external validation cohorts)

First author	Year	Risk score	Year original risk score published	Derivation country(ies)	Validation cohort	Country(ies) of validation cohort	Risk of bias	Weight (%)	C-statistic [95% CI]
vanderLeeuw	2015	ADVANCE	2011	Europe/Asia	EPIC-NL	Netherlands	Medium	6.07%	0.62 [0.54, 0.70]
vanderLeeuw	2015	ADVANCE	2011	Europe/Asia	EPIC-Potsdam	Germany	Medium	6.07%	0.67 [0.59, 0.75]
vanderLeeuw	2015	ADVANCE	2011	Europe/Asia	SMART	Netherlands	Medium	4.57%	0.68 [0.59, 0.77]
Wan	2018	ADVANCE	2011	Europe/Asia	Hong Kong Hospital Authority (men)	China	Low	27.13%	0.69 [0.68, 0.70]
Wan	2018	ADVANCE	2011	Europe/Asia	Hong Kong Hospital Authority (women)	China	Low	27.13%	0.71 [0.70, 0.72]
Read	2018	ADVANCE	2011	Europe/Asia	National Scottish Diabetes Register	Scotland	Medium	29.04%	0.67 [0.66, 0.67]
Overall								100%	0.68 [0.66, 0.70]

Random-effects REML model
I^2 = 88.44%; τ^2 = 0; P_Q = 7.2e-10; C-statistic [95% CI] = 0.68[0.66 - 0.7], P = 0

Fixed-effects model
I^2 = 90.27% ; P_Q = 7.2e-10; C-statistic [95% CI] = 0.67[0.67 - 0.68], P = 0

b

Meta-analysis for the c-statistics of 'CVD-EDIC' risk scores (external validation cohorts)

First author	Year	Risk score	Year original risk score published	Derivation country(ies)	Validation cohort	Country(ies) of validation cohort	Risk of bias	Weight (%)	C-statistic [95% CI]
vanderLeeuw	2015	CVD-EDIC	2015	United States	EPIC-NL	Netherlands	Medium	15.17%	0.58 [0.50, 0.66]
vanderLeeuw	2015	CVD-EDIC	2015	United States	EPIC-Potsdam	Germany	Medium	15.17%	0.68 [0.60, 0.76]
vanderLeeuw	2015	CVD-EDIC	2015	United States	SMART	Netherlands	Medium	10.69%	0.69 [0.59, 0.79]
Read	2018	CVD-EDIC	2015	United States	National Scottish Diabetes Register	Scotland	Medium	58.96%	0.67 [0.67, 0.67]
Overall								100%	0.66 [0.62, 0.70]

Random-effects REML model
I^2 = 37.46%; τ^2 = 0.001; P_Q = 0.167; C-statistic [95% CI] = 0.66[0.62 - 0.7], P = 3.61e-281

Fixed-effects model
I^2 = 40.8% ; P_Q = 0.167; C-statistic [95% CI] = 0.67[0.67 - 0.67], P = 0

c

Meta-analysis for the c-statistics of 'UKPDS' risk scores (external validation cohorts)

First author	Year	Risk score	Year original risk score published	Derivation country(ies)	Validation cohort	Country(ies) of validation cohort	Risk of bias	Weight (%)	C-statistic [95% CI]
Simmons	2009	UKPDS	1998	United Kingdom	Epic Norfolk	United Kingdom	Medium	15.61%	0.72 [0.66, 0.78]
Davis	2009	UKPDS	1998	United Kingdom	FDS	Australia	Medium	10.21%	0.68 [0.60, 0.76]
Kengne	2010	UKPDS	1998	United Kingdom	ADVANCE	Europe/Asia	Low	29.88%	0.68 [0.65, 0.72]
vanDieren	2011	UKPDS	1998	United Kingdom	Epic-NL; Epic-Potsdam	Netherlands/Germany	Low	3.69%	0.66 [0.51, 0.81]
Tanaka	2013	UKPDS	1998	United Kingdom	JDCS/J-EDIT	Japan	Medium	14.26%	0.69 [0.63, 0.76]
Piniés	2014	UKPDS	1998	United Kingdom	BASCORE	Spain	Low	19.81%	0.62 [0.57, 0.67]
Venäläinen	2020	UKPDS	1998	United Kingdom	Hoorn Study	Netherlands	Medium	6.53%	0.66 [0.55, 0.77]
Overall								100%	0.68 [0.65, 0.71]

Random-effects REML model
I^2 = 23.84%; τ^2 = 0; P_Q = 0.383; C-statistic [95% CI] = 0.68[0.65 - 0.71], P = 0

Fixed-effects model
I^2 = 5.84% ; P_Q = 0.383; C-statistic [95% CI] = 0.68[0.65 - 0.7], P = 0

Legend: Sensitivity Analysis Excluding High Risk of Bias Studies for ADVANCE Risk Score (**Panel a**), CVD-EDIC Risk Score (**Panel b**), and UKPDS Risk Score (**Panel c**) on external validation. A random-effects model was applied for the overall pooled estimate only when the heterogeneity test was statistically significant (Cochran’s Q test p-value <0.1 or the I^2 statistic > 75%). The analysis includes confidence intervals (CI).

Supplemental Figure 19. Quality Assessment of Biomarker Studies using modified Newcastle-Ottawa Scale

Study	D1	D2	D3	D4	D5	D6	Overall
Basu 2017	Medium	Medium	High	Low	Low	Low	High
Basu 2018	Low	Medium	Low	Medium	Low	High	High
Cederholm 2008	Low	Medium	Low	Low	Low	Low	Low
Clarke 2004	Low	Medium	Low	Low	Low	Low	Low
Cox 2014	Low	Medium	Low	Low	Low	High	High
Cox 2014a	Low	Medium	Low	Low	Low	Medium	Medium
Davis 2009	Medium	Medium	Low	Low	Low	Low	Medium
Davis 2010	Low	Medium	Low	Low	Low	High	High
Davis 2020	Low	Medium	Low	Low	Low	High	High
Donnan 2006	Low	Medium	Low	Low	Low	High	High
Elley 2010	Low	Medium	Low	Low	Low	Medium	Medium
Folsom 2003	Low	Medium	Low	Medium	Low	Low	Medium
Guzder 2005	Low	Medium	High	Low	Low	High	High
Hamada 2018	Low	Medium	Low	Low	Low	Low	Low
Hayes 2013	Low	Medium	Low	Low	Low	Low	Low
Kengne 2010	Low	Medium	Low	Low	Low	Low	Low
Kengne 2011	Low	Medium	Low	Low	Low	High	High
Lagani 2015	Medium	Medium	Low	High	Low	High	High
Li 2018	Medium	Medium	Low	Low	Low	High	High
McEwan 2015	Low	Medium	Low	Low	Low	High	High
Mentz 2018	Low	Medium	Low	Low	Low	High	High
Mukamal 2013	Low	Medium	Low	Low	Low	Low	Low
Pinias 2014	Low	Medium	Low	Low	Low	Low	Low
Quan 2019	Low	Medium	Low	Low	Low	High	High

Study	D1	D2	D3	D4	D5	D6	Overall
Ramírez-Prado 2015	Medium	Medium	Low	Low	Low	High	Medium
Read 2018	Low	Medium	Low	Medium	Low	Low	Medium
Rossi 2011	Low	Medium	Low	Low	Low	Low	Low
Shao 2018	Low	Medium	Low	Low	Low	Low	Low
Shao 2020	Medium	Medium	Low	Low	Low	Medium	Medium
Simmons 2009	Low	Medium	Low	Medium	Low	Low	Medium
Stevens 2001	Low	Medium	Low	Low	Low	Low	Low
Tanaka 2013	Low	Medium	High	Low	Low	Low	Medium
Venäläinen 2020	Medium	Medium	Low	Low	Low	Medium	Medium
Wan 2018	Low	Medium	Low	Low	Low	Low	Low
Wells 2013	Low	Medium	Low	Low	Low	Low	Low
Woodward 2016	Medium	Medium	Low	Low	Low	High	High
Yang 2008	High	Medium	Low	Medium	Low	Low	High
Yang 2013	Medium	Medium	Low	Low	Low	High	Medium
Yeboah 2014	Medium	Medium	Low	Low	Low	Low	Medium
Yoshida 2012	Medium	Medium	Low	Low	Low	Low	Medium
Young 2018	Low	Medium	Low	Low	Low	Low	Low
Yu 2019	Low	Medium	Low	Low	Low	Medium	Low
Zethelius 2011	Low	Medium	Low	Low	Low	Low	Low
Zhang 2020	Low	Medium	Low	Low	Low	Low	Low
vanDieren 2011	Low	Medium	Low	Low	Low	Low	Low
vanderHeijden 2009	Low	Medium	Low	Low	Low	Low	Low
vanderLeeuw 2015	Low	Medium	Low	Medium	Low	Low	Medium

D1 - Representativeness Bias
D2 - Selection Bias
D3 - Exposure Bias
D4 - Outcome Bias
D5 - Duration of follow-up Bias
D6 - Lost to follow-up Bias

Risk of bias: Low, Medium, High

Legend: Quality Assessment of Biomarker Studies using modified Newcastle-Ottawa Scale (Author Last Name: A-Cou). D= domain.

Supplemental Figure 20. Quality Assessment of Biomarker Studies using modified Newcastle-Ottawa Scale

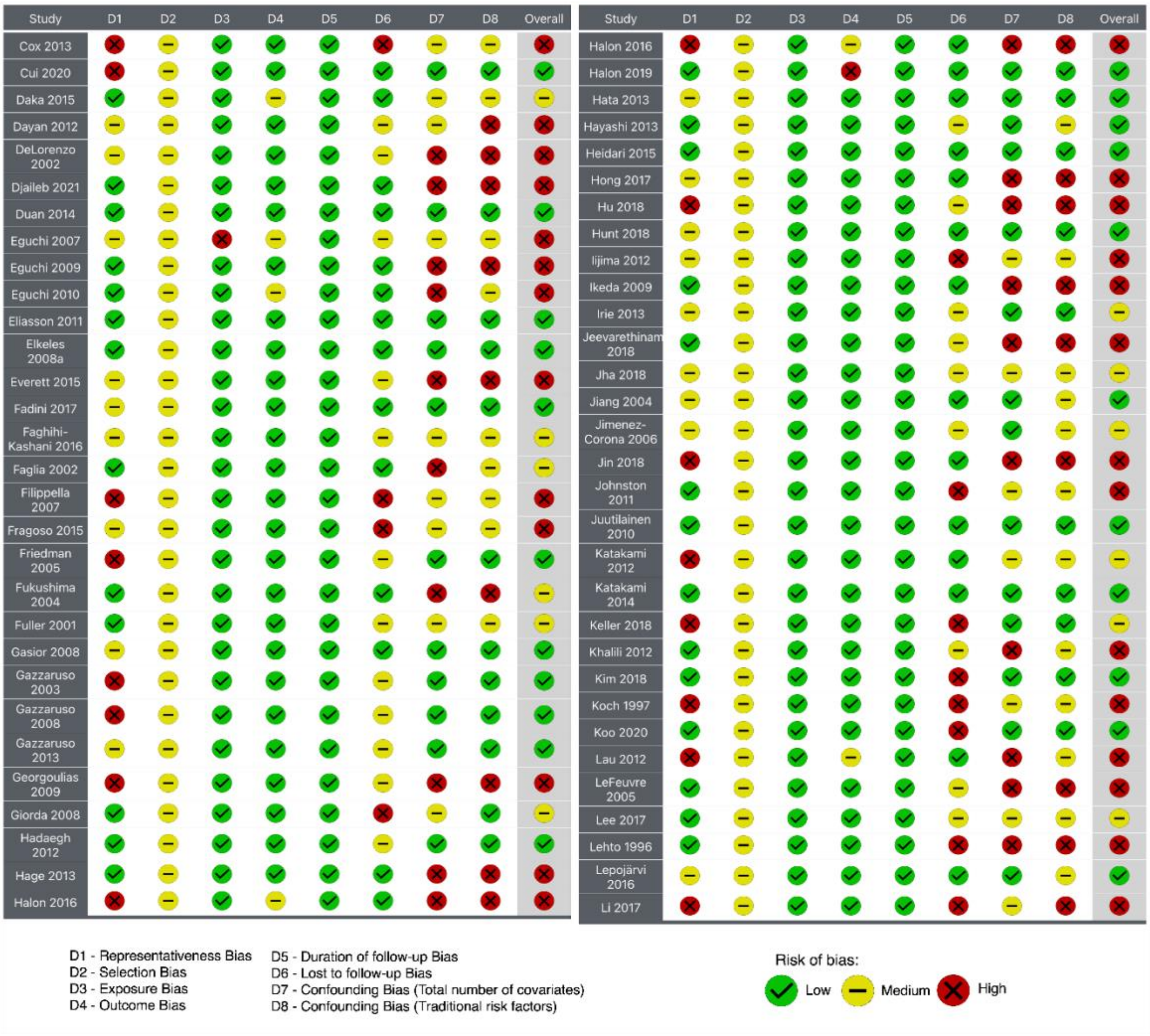

Legend: Quality Assessment of Biomarker Studies using modified Newcastle-Ottawa Scale (Author Last Name: Cox-Li). D= domain.

Supplemental Figure 21. Quality Assessment of Biomarker Studies using modified Newcastle-Ottawa Scale

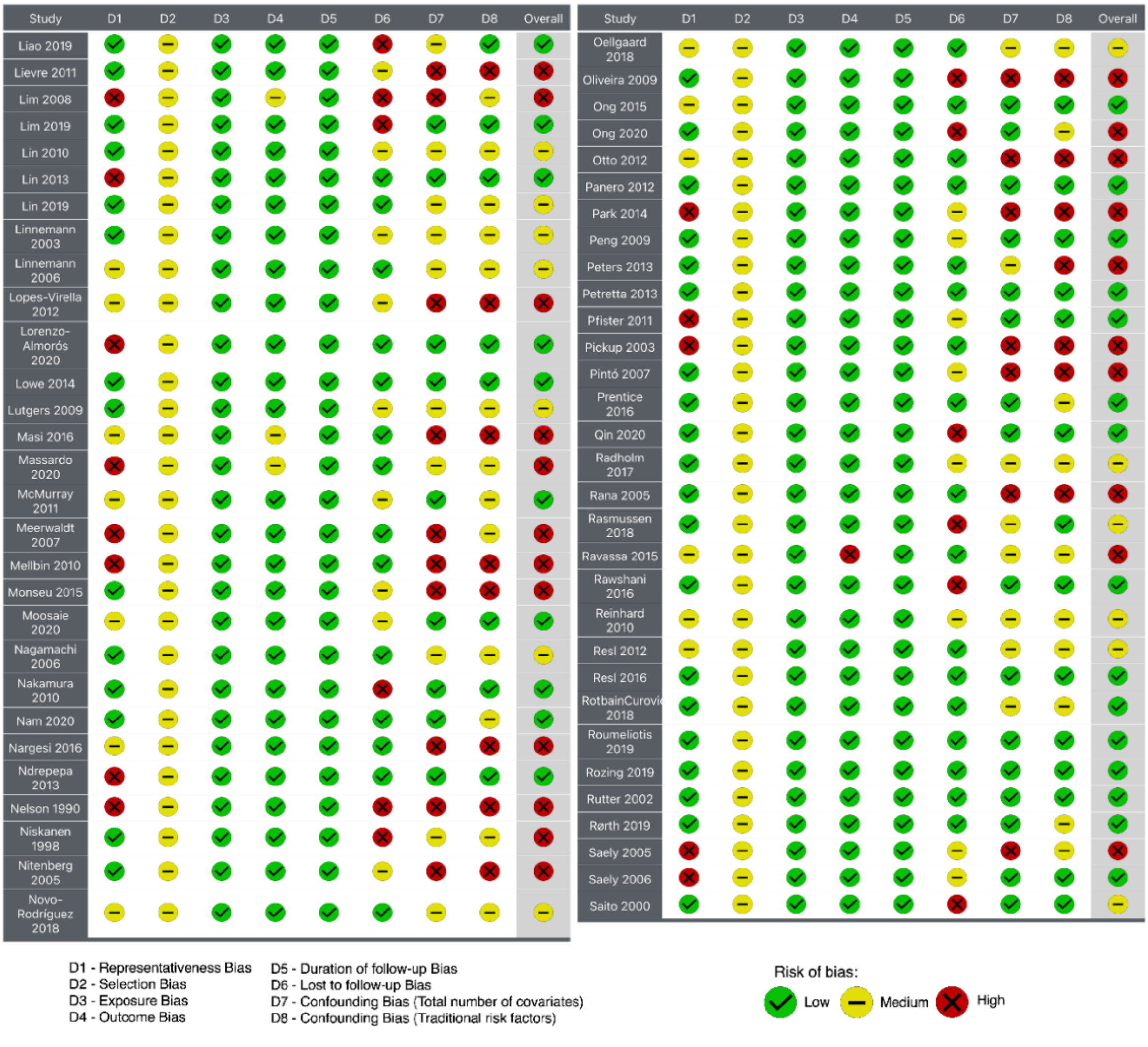

Legend: Quality Assessment of Biomarker Studies using modified Newcastle-Ottawa Scale (Author Last Name: Lia-Sai). D= domain.

Supplemental Figure 22. Quality Assessment of Biomarker Studies using modified Newcastle-Ottawa Scale

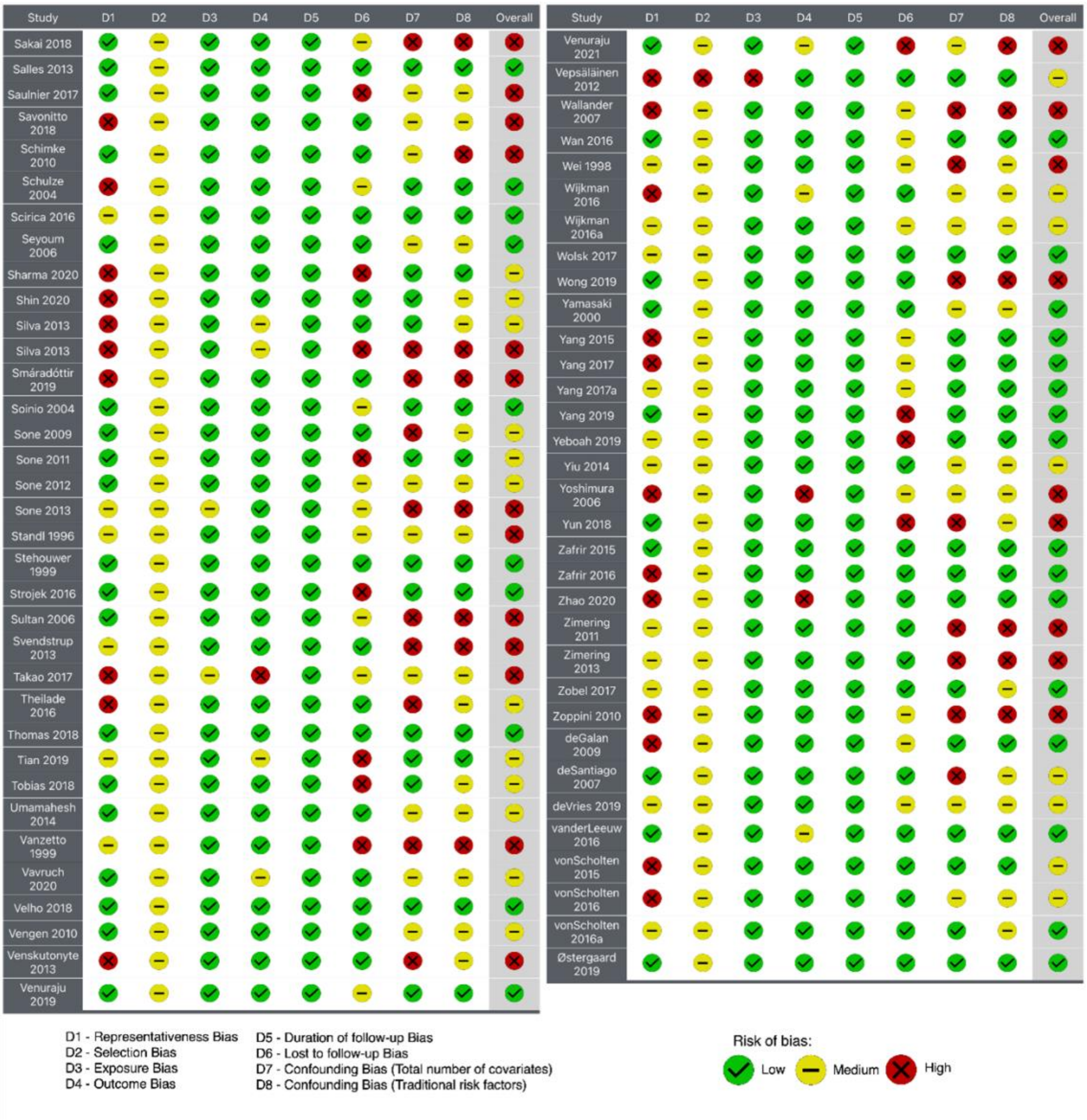

Legend: Quality Assessment of Biomarker Studies using modified Newcastle-Ottawa Scale (Author Last Name: Sak-Ost). D= domain.

Supplemental Figure 23. Quality assessment of Genetics Studies using modified Newcastle-Ottawa Scale.

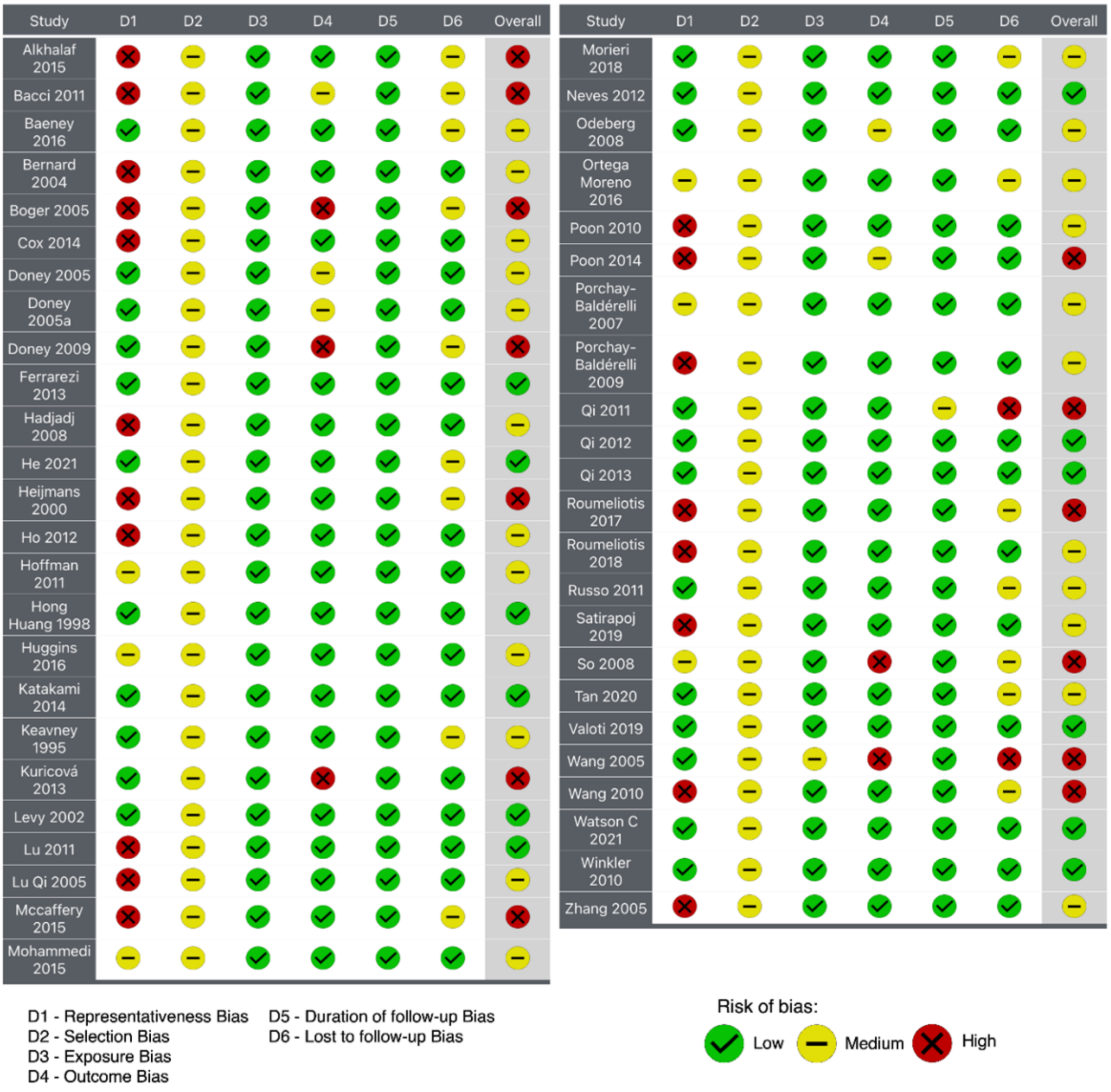

Legend: Quality assessment of Genetics Studies using modified Newcastle-Ottawa Scale. D= domain.

Supplemental Figure 24. Quality assessment of Risk Score Studies using modified Newcastle-Ottawa Scale.

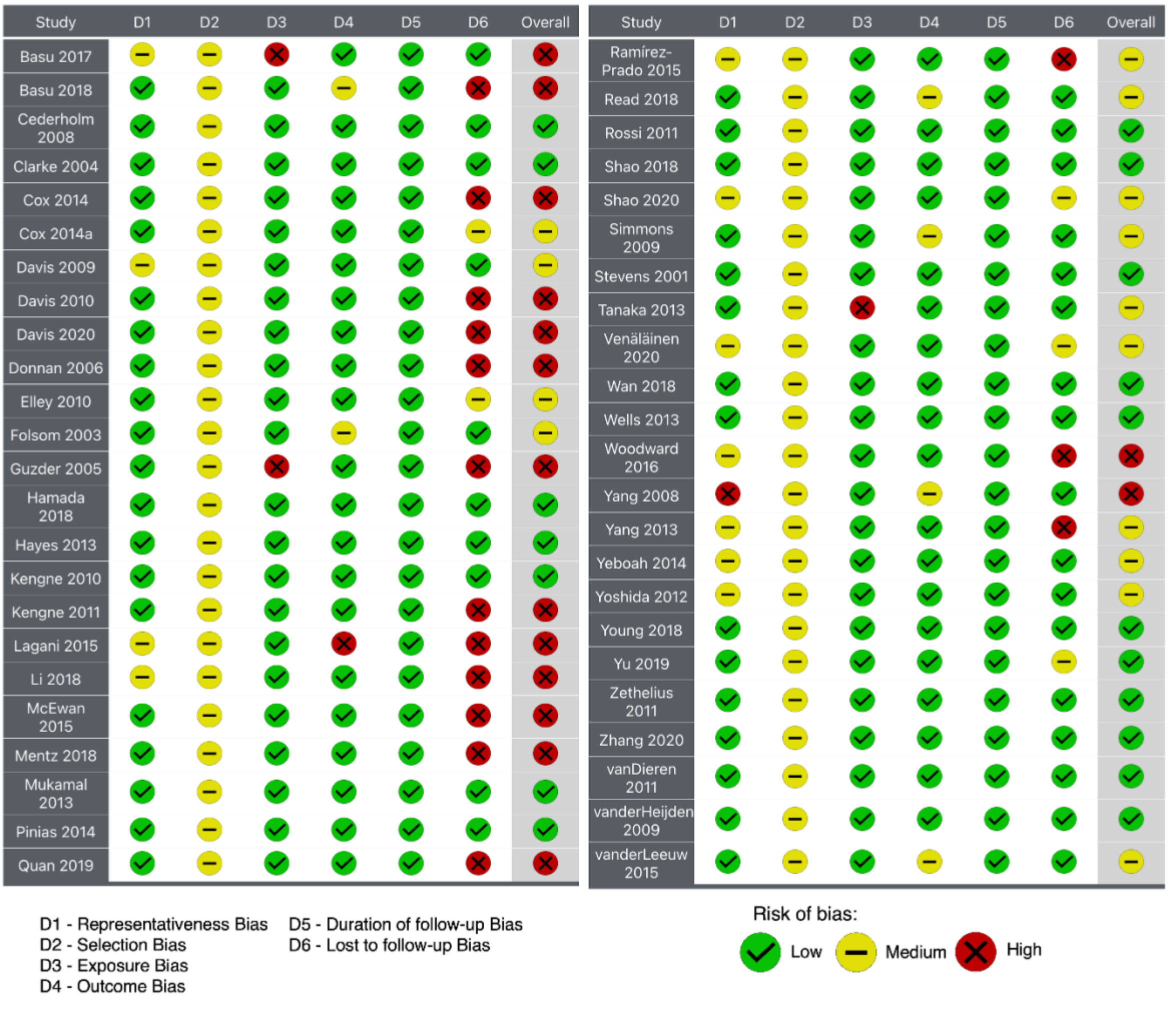

Legend: Quality assessment of Risk Score Studies using modified Newcastle-Ottawa Scale. D= domain.

Supplemental references

1. Visseren, F.L.J., *et al.* 2021 ESC Guidelines on cardiovascular disease prevention in clinical practice. *Eur Heart J* **42**, 3227-3337 (2021).
2. Hlatky, M.A., *et al.* Criteria for evaluation of novel markers of cardiovascular risk: a scientific statement from the American Heart Association. *Circulation* **119**, 2408-2416 (2009).
3. Goff, D.C., *et al.* 2013 ACC/AHA guideline on the assessment of cardiovascular risk: a report of the American College of Cardiology/American Heart Association Task Force on Practice Guidelines. *Circulation* **129**, S49-73 (2014).
