# Supplementary material for: Precision prognostics for cardiovascular disease in Type 2 diabetes: a systematic review and meta-analysis
Source: Commun Med (Lond). 2024 Jan 22;4:11. doi: 10.1038/s43856-023-00429-z (PMC10803333; doi:10.1038/s43856-023-00429-z)
Supplement: Supplementary file 2 — Description of Additional Supplementary Files [file 43856_2023_429_MOESM2_ESM.docx]

**Description of Additional Supplementary Files**

**File Name:** Supplementary Data 1

**Description:** Included 321 biomarker studies

**File Name:** Supplementary Data 2

**Description:** Included 48 genetic markers studies

**File Name:** Supplementary Data 3

**Description:** Included 47 risk score studies

**File Name:** Supplementary Data 4

**Description:** Summary of results on the associations between biomarkers and CV outcomes (248 studies on overall 195 different biomarkers)

**File Name:** Supplementary Data 5

**Description:**  Degree of variation in measurement methods used for each of these biomarkers obtained prior to CVD events in the ambulatory non-acute setting.

**File Name:** Supplementary Data 6

**Description:** Summary of the results on the association between 79 genetic biomarkers and CVD outcomes

**File Name:** Supplementary Data 7

**Description:** Summary of results from risk scores studies on internal and external validation
