# Supplementary material for: Precision prognostics for cardiovascular disease in Type 2 diabetes: a systematic review and meta-analysis
Source: Commun Med (Lond). 2024 Jan 22;4:11. doi: 10.1038/s43856-023-00429-z (PMC10803333; doi:10.1038/s43856-023-00429-z)
Supplement: Supplementary file 10 — Reporting summary [file 43856_2023_429_MOESM10_ESM.pdf]

nature portfolio

Corresponding author(s): Nestoras Mathioudakis

Last updated by author(s): 11/29/2023

Reporting Summary

Nature Portfolio wishes to improve the reproducibility of the work that we publish. This form provides structure for consistency and transparency in reporting. For further information on Nature Portfolio policies, see our Editorial Policies and the Editorial Policy Checklist.

Statistics

For all statistical analyses, confirm that the following items are present in the figure legend, table legend, main text, or Methods section.

n/a	Confirmed	
☒	☐	The exact sample size (*n*) for each experimental group/condition, given as a discrete number and unit of measurement
☒	☐	A statement on whether measurements were taken from distinct samples or whether the same sample was measured repeatedly
☐	☒	The statistical test(s) used AND whether they are one- or two-sided<br>*Only common tests should be described solely by name; describe more complex techniques in the Methods section.*
☐	☒	A description of all covariates tested
☒	☐	A description of any assumptions or corrections, such as tests of normality and adjustment for multiple comparisons
☐	☒	A full description of the statistical parameters including central tendency (e.g. means) or other basic estimates (e.g. regression coefficient) AND variation (e.g. standard deviation) or associated estimates of uncertainty (e.g. confidence intervals)
☐	☒	For null hypothesis testing, the test statistic (e.g. *F*, *t*, *r*) with confidence intervals, effect sizes, degrees of freedom and *P* value noted<br>*Give P values as exact values whenever suitable.*
☒	☐	For Bayesian analysis, information on the choice of priors and Markov chain Monte Carlo settings
☒	☐	For hierarchical and complex designs, identification of the appropriate level for tests and full reporting of outcomes
☒	☐	Estimates of effect sizes (e.g. Cohen's *d*, Pearson's *r*), indicating how they were calculated

Our web collection on statistics for biologists contains articles on many of the points above.

Software and code

Policy information about availability of computer code

Data collection	N/A. This is a systematic review and meta-analysis. All data were collected manually from review of original articles.
Data analysis	The meta-analyses were conducted in R.

For manuscripts utilizing custom algorithms or software that are central to the research but not yet described in published literature, software must be made available to editors and reviewers. We strongly encourage code deposition in a community repository (e.g. GitHub). See the Nature Portfolio guidelines for submitting code & software for further information.

Data

Policy information about availability of data

All manuscripts must include a data availability statement. This statement should provide the following information, where applicable:

- Accession codes, unique identifiers, or web links for publicly available datasets
- A description of any restrictions on data availability
- For clinical datasets or third party data, please ensure that the statement adheres to our policy

The protocol for this systematic review and meta-analysis is publicly available through the International Prospective Register of Systematic Reviews (PROSPERO), with the registration number CRD42021262843. Comprehensive search strategies that can be reproduced are outlined in Supplemental Text 1. All data generated or analyzed during this study are reported in this article and its supplemental materials. Lists of included studies are available in Supplemental table 5, 6, and 7. Supplemental Tables 8, 10, and 11 provide source data used to generate forest plots and meta-analyses. Any further details required are available from the

corresponding author upon reasonable request.
The data presented on the website https://hugofitipaldi.shinyapps.io/T2D_prognostic/ have been also uploaded in a DOI minting repository (linked to a GitHub repository): DOI Repository: https://zenodo.org/records/10277173 (https://github.com/hugofitipaldi/T2DCVD_Prognostics).

Human research participants

Policy information about studies involving human research participants and Sex and Gender in Research.

Reporting on sex and gender	We did not systematically report findings with respect to race and gender given the scope of this systematic review and met-analysis.
Population characteristics	We provide basic information about the sample sizes, underlying ASCVD risk, follow-up duration, and country of origin from each of the studies in this review.
Recruitment	Not applicable- this was a systematic review
Ethics oversight	The study protocol was registered with PROSPERO.

Note that full information on the approval of the study protocol must also be provided in the manuscript.

Field-specific reporting

Please select the one below that is the best fit for your research. If you are not sure, read the appropriate sections before making your selection.

☒ Life sciences ☐ Behavioural & social sciences ☐ Ecological, evolutionary & environmental sciences

For a reference copy of the document with all sections, see nature.com/documents/nr-reporting-summary-flat.pdf

Life sciences study design

All studies must disclose on these points even when the disclosure is negative.

Sample size	N/A
Data exclusions	N/A
Replication	N/A
Randomization	N/A
Blinding	N/A

Reporting for specific materials, systems and methods

We require information from authors about some types of materials, experimental systems and methods used in many studies. Here, indicate whether each material, system or method listed is relevant to your study. If you are not sure if a list item applies to your research, read the appropriate section before selecting a response.

Materials & experimental systems

n/a	Involved in the study	
☒	☐	Antibodies
☒	☐	Eukaryotic cell lines
☒	☐	Palaeontology and archaeology
☒	☐	Animals and other organisms
☐	☒	Clinical data
☒	☐	Dual use research of concern

Methods

n/a	Involved in the study	
☒	☐	ChIP-seq
☒	☐	Flow cytometry
☒	☐	MRI-based neuroimaging

Clinical data

Policy information about clinical studies

All manuscripts should comply with the ICMJE guidelines for publication of clinical research and a completed CONSORT checklist must be included with all submissions.

Clinical trial registration	N/A; this was a systematic review and meta-analysis.
Study protocol	The systematic review and meta-analysis protocol was registered on PROSPERO (Registration number: CRD42021262843).
Data collection	See manuscript for detailed description of data extraction for this study.
Outcomes	See manuscript for detailed description of outcome definitions for this study.
